# Synthesis of Sucrose-Mimicking Disaccharide by Intramolecular Aglycone Delivery

**DOI:** 10.3390/molecules29081771

**Published:** 2024-04-13

**Authors:** Kanae Sano, Akihiro Ishiwata, Hiroto Takamori, Takashi Kikuma, Katsunori Tanaka, Yukishige Ito, Yoichi Takeda

**Affiliations:** 1 Department of Biotechnology, College of Life Sciences, Ritsumeikan University, Kusatsu 525-8577, Japan; ksano@gst.ritsumei.ac.jp (K.S.); tkikuma@fc.ritsumei.ac.jp (T.K.); 2 RIKEN Cluster for Pioneering Research, Wako 351-0198, Japan; kotzenori@riken.jp (K.T.); yukito@chem.sci.osaka-u.ac.jp (Y.I.); 3 Department of Chemical Science and Engineering, Tokyo Institute of Technology, Tokyo 152-8552, Japan; 4 Graduate School of Science, Osaka University, Toyonaka 560-0043, Japan

**Keywords:** rare sugar, psicose, allose, intramolecular aglycone delivery, glycosylation

## Abstract

Rare sugars are known for their ability to suppress postprandial blood glucose levels. Therefore, oligosaccharides and disaccharides derived from rare sugars could potentially serve as functional sweeteners. A disaccharide [α-d-allopyranosyl-(1→2)-β-d-psicofuranoside] mimicking sucrose was synthesized from rare monosaccharides D-allose and D-psicose. Glycosylation using the intermolecular aglycon delivery (IAD) method was employed to selectively form 1,2-*cis* α-glycosidic linkages of the allopyranose residues. Moreover, β-selective psicofuranosylation was performed using a psicofuranosyl acceptor with 1,3,4,6-tetra-*O*-benzoyl groups. This is the first report on the synthesis of non-reducing disaccharides comprising only rare d-sugars by IAD using protected ketose as a unique acceptor; additionally, this approach is expected to be applicable to the synthesis of functional sweeteners.

## 1. Introduction

**Figure 1:**
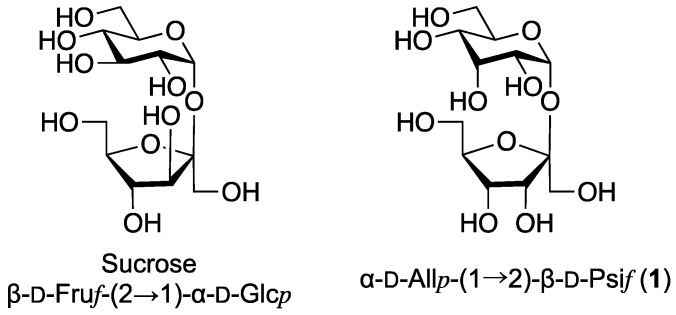
Structure of sucrose and the targeted α-D-allopyranosyl-(1→2)-β-D-psicofuranoside (**1**).

Rare sugars are saccharides that exist in small quantities in nature and have attracted attention for their functions such as their ability to suppress postprandial blood glucose elevation [1,2]. Recently, enzymatic syntheses of monosaccharide rare sugars such as psicose and allose have been established [3,4,5], enabling their mass production and availability; it has also been reported that several rare sugars can be chemically synthesized via site-selective epimerization [6,7]. With this progress, novel oligosaccharides and glycosides derived from these monosaccharides and rare sugars by chemical synthesis could result in the creation of new functional food products. Therefore, we considered that synthesizing a novel pseudo sucrose [α-D-allopyranosyl-(1→2)-β-D-psicofuranose] (**1**) using rare monosaccharides could serve as a functional sweetener to replace sucrose [8,9], the most common sweetener. Excessive intake of sucrose has been known to cause diabetes and has been a global concern for many years. The disaccharide structure of sucrose [β-D-fructofuranosyl-(2→1)-α-D-glucopyranoside] (Figure 1) contains α- and β-glycosidic linkages between the glucopyranose and fructofuranose anomers at both anomeric positions. Therefore, to form the desired isomer of the pseudo sucrose disaccharide, it is necessary to control the stereoselectivity of glycosylation between the anomeric positions of the two rare sugars. This is because four stereoisomers of the pseudo sucrose disaccharide can be generated from glycosylation.

**Figure 2:**
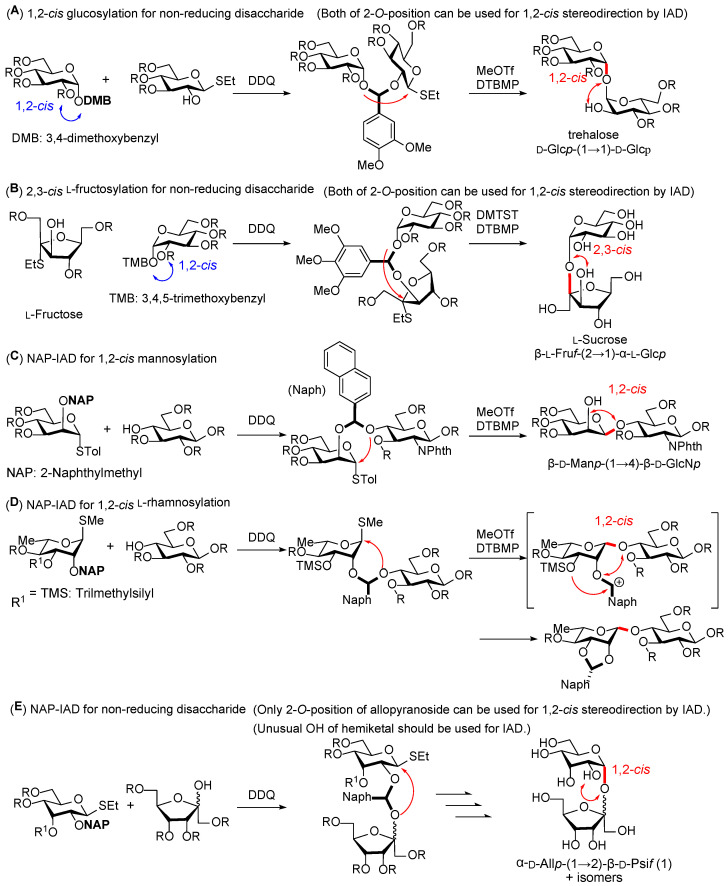
Stereoselective glycosylation using IAD. (**A**) 1,1-α,α-Glycoside formation between aldose derivatives [12,13]. (**B**) 2,3-*cis* β-L-Fructosylation by IAD with trimethoxybenzyl ether [14]. (**C**) β-Mannosylation by the NAP-IAD method [15]. (**D**) β-L-Rhamnosylation by the NAP-IAD method [16]. (**E**) α-Allosylation with psicose derivatives by the NAP-IAD method (this study). Red arrow indicates the intramolecular transfer of the aglycon. Blue and red double arrows indicate *cis* configurations introduced in the substrates and formed as the products, respectively. The newly formed cis-glycosides were colored in red.

One of the difficulties in synthesizing the desired α-allosidic linkage is the selective formation of 1,2-*cis*-glycosidic linkages. For this, stereoselective methods with various enhancements of the anomeric effect [10,11] and intramolecular aglycon delivery (IAD) reactions [12,13,14,15,16] have been developed. Bertozzi et al. employed stereoselective 1,2-*cis*-glycosylation to synthesize trehalose derivatives in high yields using dimethoxybenzyl ether-mediated IAD for the stereoselective formation of 1,1-α,α-glycosidic linkages between aldose derivatives [17,18] (Figure 2A). Although the IAD method has been used to synthesize non-reducing disaccharides like L-sucrose through trimethoxybenzyl ether-mediated IAD for 2,3-*cis* β-L-fructosylation (Figure 2B), it cannot be applied to synthesize the desired β-D-psicofuranoside because of its configuration [19]. In previous studies, 2-naphthylmethyl ether (NAP)-mediated IAD was used for 1,2-*cis*-mannosylation and 1,2-*cis*-rhamnosylation (Figure 2C,D) [20,21]. Therefore, we chose to apply the versatile NAP-mediated IAD for 1,2-*cis*-glycosylation to synthesize the target non-reducing pseudo sucrose disaccharide (Figure 2E).

Because of the reducing sugar structure of our target compound (**1**), retrosynthetic disconnection for glycosylation can be designed in two ways: by forming β-D-psicofuranoside or α-D-allopyranoside. In previous studies on the selective β-psicofuranosylation, Ueda et al. and Yamanoi et al. reported the synthesis of psicose derivatives as donors with leaving groups (benzyl phthalate and acetate, respectively) and optimized the glycosylation conditions by investigating protecting groups in the presence of TMSOTf and Sc(OTf)_3_, respectively [22,23,24]. To date, only a few studies have used psicose derivatives with hydroxy groups as the required acceptors for allopyranosylation to synthesize pseudo sucrose disaccharides. We decided to use 1,3,4,6-tetra-*O*-benzoyl psicose as the acceptor because of the simplicity of its one-step derivation from psicose according to a reported method [25,26]. 

In this study, we synthesized a pseudo sucrose disaccharide (**1**) by α-D-allosylation using NAP-IAD between the rare sugars D-allopyranose and D-psicofuranose.

## 2. Results and Discussion

**Scheme 1:**
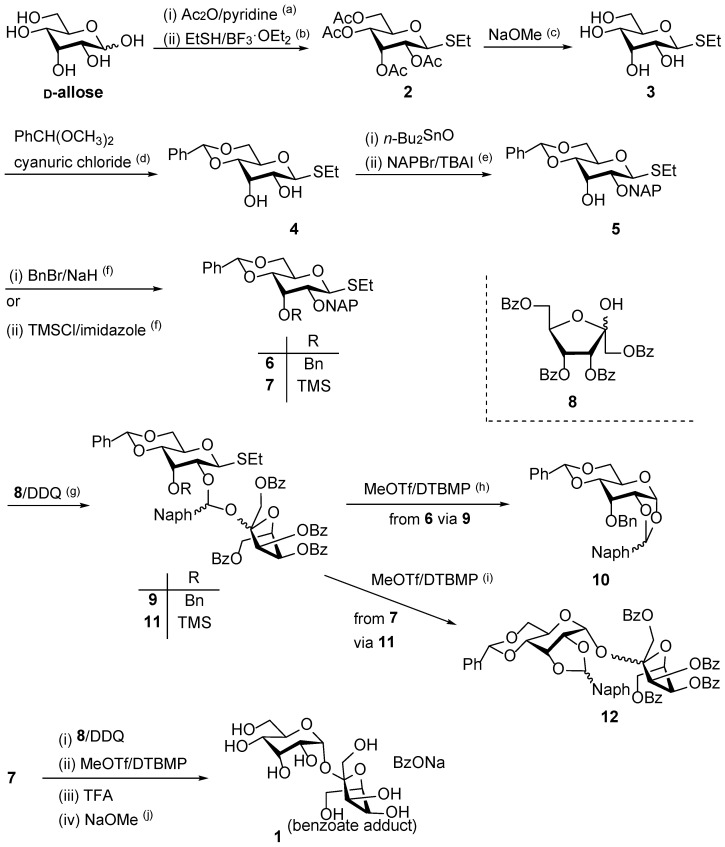
Synthesis of the allose donor and the allosylation reaction thorough NAP-IAD. (a) Ac_2_O, pyridine, 10 h, 40 °C, quant. (b) Ethanethiol, boron trifluoride diethylether complex, 1,2-dichloroethane, 12 h, r.t., 66% (α/β ratio of 23:77). (c) Sodium methoxide, THF, MeOH, 10 h, r.t., quant. (d) PhCH(OCH_3_)_2_, cyanuric chloride, DMF, 2.5 h, r.t., 29%. (e) (i) di-*n*-butyltin oxide, toluene, 2 h, 120 °C; (ii) NAPBr, TBAI, CsF, 3 h, r.t., 52%. (f) (i) BnBr, NaH, DMF, 1.5 h, r.t., 99% for **6**; (ii) TMSCl, imidazole, DMF, 3 h, r.t., 99% for **7**. (g) **8**, DDQ, MS4Å, CH_2_Cl_2_, 1 d, r.t. for **9**; **8**, DDQ, MS4Å, 1,2-dichloroethane, 19 h, r.t. for **11**. (h) MeOTf, 2,6-di-*tert*-butyl-4-methylpyridine, MS4Å, 1,2-dichloroethane, 1 d, 40 °C, 49%. (i) MeOTf, 2,6-di-*tert*-butyl-4-methylpyridine, MS4Å, 1,2-dichloroethane, 20 h, 40 °C, 19%. (j) (i) **8**, DDQ, MS4Å, 1,2-dichloroethane, 1 d, r.t.; (ii) MeOTf, 2,6-di-*tert*-butyl-4-methylpyridine, MS4Å, 1,2-dichloroethane, 1 d, 40 °C; (iii) TFA, CH_2_Cl_2_, 1 min, 0 °C, 4% from **7**; (iv) sodium methoxide, MeOH, 5 h, r.t., quant.

We first attempted the stereoselective α-D-allosylation using the NAP-IAD glycosylation method. The D-allosyl donor with an NAP group at the C-2 position was synthesized from D-allose in six steps. As shown in Scheme 1, the first step involved the acetylation of the hydroxy group of D-allose with Ac_2_O in pyridine. The peracetate was then converted to the thioglycoside as an α/β glycoside mixture (α/β ratio of 23:77) at a yield of 66%, resulting in the recovery of the required β-glycoside-rich fraction. Deacetylation of **2**, followed by the regioselective formation of 4,6-*O*-benzylidene acetal on the resultant tetraol **3**, selectively yielded the desired compound **4**. In this step, the α-thioglycoside was completely removed as this anomeric functionality was required for the subsequent general activation with MeOTf in the IAD reaction. However, this process resulted in a yield of 29% over two steps, with 2,3-*O*-benzylidene acetal and 2,3,4,6-di-*O*-benzylidene obtained as byproducts at 6% and 7% yields, respectively. The remaining hydroxy groups of β-thioglycoside **4** at the C-2 and C-3 positions were protected regioselectively. Initially, the hydroxy group at the C-2 position was protected as an NAP ether, affording the resultant compound **5** at a 52% yield. The hydroxy group at the C-3 position was protected as a benzyl ether to afford **6** at a 99% yield. Following this, the D-allosyl donor **6** was oxidized using 2,3-dichloro-5,6-dicyanobenzoquinone (DDQ) in the presence of 1,3,4,6-tetra-*O*-benzoyl-D-psicofuranose (**8**) as the acceptor. This reaction produced mixed acetal isomers **8**, forming a simple diastereomeric mixture related to naphthylidene acetal. The major acetal isomer was then isolated, and its subsequent intramolecular glycosylation was performed using MeOTf and 2,6-di-*tert*-butyl-4-methylpyridine (DTBMP) in 1,2-dichloroethane. The major isolated compound was an unexpected 1,2-*O*-naphthylidene acetal of the donor moiety (**10**) at a 49% yield. The 1,2-*O*-benzylidene-type cyclization from 1,2-*cis* glycoside was possible owing to the formation of 1,2-*cis*-glycoside in the IAD [27]. The configuration of allose may support the formation of 1,2-*O*-benzylidene acetal when the 3-OH of 1,2-*cis*-allopyranoside was protected as a Bn ether, which created a steric hinderance to the naphthylmethyl group at the C-2 position. As previously reported, the activation of ketosidic bonds is largely influenced by the stereochemistry in the sugar ring, which is not clearly understood [28]. This may have occurred because of the initial nucleophilic attack of the oxygen atom to the resultant naphthylmethyl cation at the 2-position of D-psicofuranoside in the 1,2-*O*-naphthylidene acetal formation, leading to the undesired cleavage of the C–O glycosidic bond of 1,2-*cis*-D-psicofuranoside. This cleavage likely occurred concomitantly with the enhancement of neighboring group participation by one of the carbonyl oxygens of the benzoyl groups within the D-psicofuranoside moiety. To produce the desired disaccharide derivative, it is necessary to trap the cation species initially on the naphthylmethyl group after glycosidic bond formation. The enhancement effect on the cleavage of the glycosidic bond has been also found in the case of the super arming effect of 2-*O*-benzoylated glycosyl donor reported by Demchemko [29,30], although we speculate that the electron withdrawing group as the protective group on the psicose stabilizes the psicosidic linkage simply by the disarming effect. Previous studies utilized (TMS)_3_SiH for the in situ reductive trapping of the benzylic cation of the NAP ether [20,21]. However, the side reaction could not be suppressed, suggesting that the cation species resulting from the fragmentation of D-psicofuranoside is more stable than the benzylic cation.

**Figure 3:**
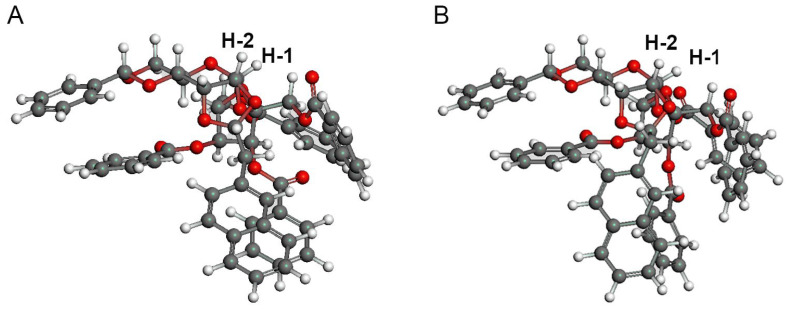
Optimized geometries of derivative **12** (absolute configurations of naphthylidene acetals (**A**,**B**) are *R* and *S*, respectively). (**A**): Dihedral angle (H-2–C-2–C-1–H-1) = 37.6° (293.4 kcal/mol, population 12.0%). (**B**): Dihedral angle (H-2–C-2–C-1–H-1) = 40.2° (291.3 kcal/mol, population 12.6%). To obtain all conformational isomers of derivative **12**, a conformational search for compounds was performed in the CONFLEX 9 software using molecular mechanics force field (MMFF94s) calculations with a search limit of 1.0 kcal/mol.

To prevent the unfavorable formation of 1,2-*O*-naphthylidene acetal, we attempted IAD using the D-allosyl donor **7**, which contained a trimethyl silyl (TMS) group at the C-3 position to trap the naphthylmethyl cation in situ by immediate acetal formation [21]. The trimethylsilylation of derivative **5** proceeded with a yield of 99%. The desired mixed acetal **11** was generated by oxidizing derivative **7** with DDQ, which was confirmed using thin layer chromatography (TLC), MALDI-TOF MS, and NMR spectroscopy. Meanwhile, the actual glycosylation reaction was carried out using MeOTf and DTBMP in 1,2-dichloroethane without purifying the mixed acetals **11**. This reaction afforded the 1,2-*cis*- D-allosyl-(1→2)-β-D-psicofuranose derivative **12**, with a 2,3-*O*-naphthylidene acetal group on the D-allosyl moiety (19%). In this reaction, 1,2-*O*-naphthylidene formation was not detected by MALDI-TOF MS. This result indicates the importance of introducing the nucleophilic 3-*O*-TMS ether to intramolecularly trap benzylic cation species, rather than intermolecularly trapping them using (TMS)_3_SiH. In this reaction, 35% of the psicose acceptor was recovered. Based on the TLC analysis, it is possible that the tethered intermediate does not form, that the acetal is cleaved even in the presence of DTBMP as a proton trap, or that the product is cleaved during formation. The coupling constant between the protons at the C-1 and C-2 positions of the allose in derivative **12** is relatively large. However, in the structures obtained using conformational analysis, the dihedral angles of the protons between the C-1 and C-2 protons in the α-alloside were approximately 37°–41°, regardless of the conformers derived from the naphthylidene group, suggesting that the product was an α-anomer (Figure 3). 

The final target compound **1** was synthesized from compound **7** in four steps without purification. The protecting groups of the coupling products were removed by a short treatment (<1 min) with trifluoroacetic acid (TFA) to prevent glycosidic bond cleavage; nevertheless, the yield of the target compound was very low (4%). Based on the TLC analysis, it is possible that the disaccharide formed is cleaved into monosaccharides. Subsequent removal of benzoyl groups was achieved by treatment with sodium methoxide in methanol, quantitatively affording the target disaccharide as a benzoate adduct. The formation of this adduct was confirmed by ^1^H and ^13^C NMR spectroscopy as well as mass spectrometry. High resolution MS indicated the formation of the desired disaccharide (calcd. for C_12_H_22_NaO_11_ [M + Na]^+^ 365.1054; found 365.1046). It has been suggested that the disaccharide, which has a *cis*-oriented oxygen functionality around the glycosides, exhibits a strong ionophore-like ability to uptake metal ions such as Na^+^ ions along with counter anions such as BzO^−^. However, the NMR spectra of the final compound indicates that it does not release the salt during purification.

The glycosidic linkages of the allosyl residue in the products were determined based on the ^1^*J*_C–H_ coupling constant (170 Hz for β or >170 Hz for α) [31,32]. The ^1^*J*_C–H_ coupling of the deprotected disaccharide was measured at 180 Hz, indicating the formation of an α-allosidic linkage. The anomeric configurations of the psicosyl residue were confirmed with reference to a previous study by Morimoto et al. [33]. The ^13^C chemical shift of the C-2 carbon of β-psicoside was recorded as 109.2 ppm (Table 1). In a previous study, a value of 111.9 Hz was reported when the chemical shift was referenced to the signal corresponding to the methyl group of sodium 3-(trimethylsilyl)[2,2,3,3-^2^H_4_] propionate (TSP-*d*_4_). The overall chemical shift values shifted downfield by approximately 2 ppm compared to those observed in D_2_O. Additionally, the stereoisomer α-D-allosyl-(1→2)-α-D-psicofuranoside was synthesized using the conventional glycosylation method (NIS/TfOH) with ethyl 2,3,4,6-tetra-*O*-benzyl-1-thio-β-D-allopyranoside, and the NMR spectra of the two stereoisomers were compared (Supporting Information Schemes S1 and S2, and Table S1). The ^13^C chemical shift of the C-2 carbon in the deprotected products obtained via NAP-IAD glycosylation was recorded as approximately 106 ppm, which did not match the reference data. The ^13^C chemical shifts of the C1 peaks of methyl α-D-glucopyranoside and trehalose (α-D-glucopyranosyl-(1→1)-α-D-glucopyranoside) were recorded as 101 ppm [34] and 94 ppm [35], respectively. This difference in the chemical shift value may have been caused by the reduced shielding effect of oxygen between the two sugar moieties in trehalose compared to that of a simple aglycone. Hence, the conformation of the psicofuranoside bond was determined to be β, and the NAP-IAD reaction was confirmed to result in β-psicofuranosylation.

## 3. Materials and Methods

### 3.1. General Methods

^1^H NMR and ^13^C NMR spectra were recorded on a JEOL (Akishima, Japan) ECS-400 spectrometer at 400 and 100 MHz, respectively. The ^1^H NMR chemical shifts were referenced to the signals of Me_4_Si as the internal standard (0.00 ppm in CDCl_3_ and HDO, and 4.80 ppm in D_2_O). The ^13^C NMR chemical shifts were referenced to the signals of the solvent [δ_C_ (CDCl_3_) 77.0] and native scale (D_2_O). Assignments were aided by COSY, TOCSY, and ^1^H–^13^C correlation experiments. All reactions were monitored by TLC using a glass plate coated with silica gel 60F254 (0.2 mm thickness, Merck KGaA, Darmstadt, Germany). Silica gel column chromatography was performed using 60N silica gel (Kanto Chemical, Tokyo, Japan). Anhydrous solvents (superdehydrated grade) were purchased from FUJIFILM Wako Pure Chemical Corp (Osaka, Japan). Optical rotations were measured with a JASCO (Hachioji, Japan) P2200 polarimeter. High resolution mass spectra (HRMS) were recorded on Bruker (Billerica, MA, USA) micrOTOF II and Thermo Scientific (Waltham, MA, USA) Exactive Plus spectrometers using electrospray ionization in acetonitrile or methanol. MALDI-TOF MS was carried out using an Autoflex Speed mass spectrometer (Billerica, MA, USA).

### 3.2. Synthesis of Compound ***2***

To a solution of D-allose (10.0 g, 55.5 mmol) in pyridine (60.0 mL), acetic anhydride (52.0 mL, 555 mmol) was added at 0 °C. The reaction mixture was stirred at 40 °C for 10 h and then quenched with MeOH at 0 °C. The reaction mixture was azeotropically dried three times with dry toluene; the resulting residue was diluted with ethyl acetate and washed successively with 1 M HCl, brine, saturated aq. NaHCO_3_, and brine. The organic layer was dried with Na_2_SO_4_, filtered, and evaporated in vacuo. The resulting residue was purified by flash silica gel column chromatography using a hexane–ethyl acetate (3/2, *v/v*) mixture to afford **1** (22.9 g, quant.). To a solution of **1** (22.9 g) and ethanethiol (5.1 mL, 70.4 mmol) in 1,2-dichloroethane (100 mL), boron trifluoride diethyl ether complex (11.0 mL, 88.1 mmol) was added at 0 °C. The reaction mixture was stirred at room temperature for 12 h and then quenched with saturated aq. NaHCO_3_ at 0 °C. The reaction mixture was diluted with chloroform and washed successively with saturated aq. NaHCO_3_ and brine. The organic layer was dried with MgSO_4_, filtered, and evaporated in vacuo. The resulting residue was purified by flash silica gel column chromatography using a hexane–ethyl acetate (1/1, *v/v*) mixture to afford compound **2** (14.5 g, 36.9 mmol, 66%, α/β ratio of 23:77). (**2α**) *R_f_* = 0.51 (hexane/ethyl acetate = 1/1, *v*/*v*); [α]^23^_D_ +159.36 (*c* 1.00, CHCl_3_); ^1^H-NMR (400 MHz, CDCl_3_) δ 5.60 (t, 1H, *J* = 3.2 Hz, H-3), 5.53 (d, 1H, *J* = 5.6 Hz, H-1), 5.11 (dd, 1H, *J* = 3.2, 6.4 Hz, H-2), 4.95 (dd, 1H, *J* = 2.4, 10.4 Hz, H-4), 4.59 (ddd, 1H, *J* = 1.6, 4.0, 10.4 Hz, H-5), 4.33 (dd, 1H, *J* = 4.8, 12.4 Hz, H-6), 4.16 (dd, 1H, *J* = 2.4, 12.4 Hz, H-6), 2.63–2.57 (m, 2H, SC*H*_2_CH_3_), 2.19 (s, 3H, COC*H*_3_), 2.09 (s, 3H, COC*H*_3_), 2.07 (s, 3H, COC*H*_3_), 2.02 (s, 3H, COC*H*_3_), 1.28 (t, 3H, *J* = 8.4 Hz, SCH_2_C*H*_3_). ^13^C-NMR (100 MHz, CDCl_3_) δ 170.7 (*C*OCH_3_), 170.0 (*C*OCH_3_), 169.6 (*C*OCH_3_), 169.3 (*C*OCH_3_), 82.9 (C-1), 67.7 (C-2), 67.5 (C-3), 65.7 (C-4), 64.3 (C-5), 62.1 (C-6), 26.7 (S*C*H_2_CH_3_), 20.9 (CO*C*H_3_), 20.7 (CO*C*H_3_), 20.6 (CO*C*H_3_), 15.3 (SCH_2_*C*H_3_). (**2β**) *R_f_* = 0.56 (hexane/ethyl acetate = 1/1, *v*/*v*); [α]^24^_D_ + 0.42 (*c* 1.00, CHCl_3_); ^1^H-NMR (400 MHz, CDCl_3_) δ 5.67 (t, 1H, *J* = 2.8 Hz, H-3), 5.01–4.95 (m, 2H, H-2, H-4), 4.83 (d, 1H, *J* = 10.4 Hz, H-1), 4.21 (m, 2H, H-6), 4.05 (td, 1H, *J* = 3.6, 10.4 Hz, H-5), 2.98–2.64 (m, 2H, SC*H*_2_CH_3_), 2.18 (s, 3H, COC*H*_3_), 2.08 (s, 3H, COC*H*_3_), 2.05 (s, 3H, COC*H*_3_), 2.01 (s, 3H, COC*H*_3_), 1.29 (t, 3H, *J* = 7.2 Hz, SCH_2_C*H*_3_). ^13^C-NMR (100 MHz, CDCl_3_) δ 170.8 (*C*OCH_3_), 169.8 (*C*OCH_3_), 169.1 (*C*OCH_3_), 169.1 (*C*OCH_3_), 80.4 (C-1), 72.5 (C-5), 68.3 (C-3), 67.6 (C-2), 66.3 (C-4), 62.5 (C-6), 23.9 (S*C*H_2_CH_3_), 20.8 (CO*C*H_3_), 20.7 (CO*C*H_3_), 20.5 (CO*C*H_3_), 14.9 (SCH_2_*C*H_3_). HRMS ESI-TOF: calcd. for C_16_H_24_NaO_9_S [M + Na]^+^ 415.1051; found 415.1049.

### 3.3. Synthesis of Compound ***4***

To allose derivative **2** (3.0 g, 7.65 mmol), a 1 M solution of sodium methoxide in methanol (0.77 mmol, 770 μL) in a mixture of tetrahydrofuran (15.0 mL) and methanol (15.0 mL) was added at 0 °C and stirred at room temperature for 10 h. The reaction solution was neutralized using an Amberlyst at 0 °C. After diluting the reaction solution with methanol, filtration with a cotton plug was carried out, and the solvent was removed to afford the white powder **3** (3.2 g), which was used without further purification. Benzaldehyde dimethyl acetal (0.96 mL, 6.43 mmol) and 2,4,6-trichloro [1,3,5]triazine (0.30 g, 1.61 mmol) were added to the obtained powder **3** (1.2 g, 5.36 mmol) in DMF (30.0 mL) at 0 °C and stirred at room temperature for 2.5 h. The reaction solution was diluted with ethyl acetate and washed with saturated aq. NaHCO_3_ and brine. The organic layer was dried with Na_2_SO_4_ and concentrated under reduced pressure. The resulting reaction mixture was purified by silica gel column chromatography using a hexane–ethyl acetate (2/3, *v*/*v*) mixture to afford derivative **4** (483 mg, 1.55 mmol, 29%). *R_f_* = 0.49 (hexane/ethyl acetate = 2/3, *v*/*v*); [α]^24^_D_ − 25.35 (*c* 1.00, CHCl_3_); ^1^H-NMR (400 MHz, CDCl_3_) δ 7.52–7.34 (m, 5H, aromatic *H*), 5.57 (s, 1H, PhC*H*), 4.78 (d, 1H, *J* = 10.4 Hz, H-1), 4.43–4.37 (m, 2H, H-3, H-6), 4.02 (dt, 1H, *J* = 5.2, 10.0 Hz, H-5), 3.74 (t, 1H, *J* = 10.0 Hz, H-6), 3.61 (dd, 1H, *J* = 2.8, 9.6 Hz, H-4), 3.57–3.53 (m, 1H, H-2), 2.81–2.72 (m, 2H, SC*H*_2_CH_3_), 2.70 (d, 1H, *J* = 6.0 Hz, OH), 2.59 (s, 1H, O*H*), 1.33 (t, 3H, *J* = 8.0 Hz, SCH_2_C*H*_3_). ^13^C-NMR (100 MHz, CDCl_3_) δ 136.9–126.2 (aromatic *C*), 101.9 (Ph*C*H), 83.8 (C-1), 78.7 (C-4), 70.0 (C-2), 69.0 (C-6), 68.2 (C-3), 66.1 (C-5), 24.7 (S*C*H_2_CH_3_), 15.3 (SCH_2_*C*H_3_). HRMS ESI-TOF: calcd. for C_15_H_20_NaO_5_S [M + Na]^+^ 335.0924; found 335.0928.

### 3.4. Synthesis of Compound ***5***

To the allose derivative **4** (483 mg, 1.55 mmol), dibutyltin(IV) oxide (426 mg, 1.71 mmol) in toluene (30.0 mL) was added and stirred at 120 °C for 2 h. The reaction solution was cooled to room temperature and the solvent was removed. To a solution of the resulting reaction mixture in toluene (30.0 mL), 2-bromomethylnaphthalene (483 mg, 2.44 mmol), tetrabutylammonium iodide (860 mg, 2.33 mmol), and cesium fluoride (354 mg, 2.33 mmol) were added at room temperature for 3 h. The reaction solution was cooled to room temperature and diluted with ethyl acetate. The reaction mixture was filtered through Celite and washed with saturated aq. NaHCO_3_ and brine. The organic layer was dried with MgSO_4_, filtered, and evaporated in vacuo. The resulting residue was purified by silica gel column chromatography using a toluene–ethyl acetate (3/2, *v*/*v*) mixture to afford compound **5** (365 mg, 0.81 mmol, 52%). *R_f_* = 0.54 (hexane/ethyl acetate = 3/2, *v*/*v*); [α]^24^_D_ − 50.21 (*c* 0.50, CHCl_3_); ^1^H-NMR (400 MHz, CDCl_3_) δ 7.87–7.32 (m, 12H, aromatic *H*), 5.49 (s, 1H, PhC*H*), 4.97–4.94 (m, 2H, H-1, NAPC*H*_2_), 4.88 (d, 1H, *J* = 12.4 Hz, NAPC*H*_2_), 4.37 (dd, 1H, *J* = 5.2, 10.8 Hz, H-6), 4.33 (brdd, 1H, *J* = 2.4, 5.2 Hz, H-3), 4.04 (dt, 1H, *J* = 5.6, 10.0 Hz, H-5), 3.69 (t, 1H, *J* = 10.4 Hz, H-6), 3.49 (dd, 1H, *J* = 2.0, 9.6 Hz, H-4), 3.43 (dd, 1H, *J* = 2.8, 9.6 Hz, H-2), 2.85–2.70 (m, 2H, SC*H*_2_CH_3_), 2.50 (s, 1H, O*H*), 1.34 (t, 3H, *J* = 8.0 Hz, SCH_2_C*H*_3_). ^13^C-NMR (100 MHz CDCl_3_) δ 137.0–126.0 (aromatic *C*), 101.9 (Ph*C*H), 81.9 (C-1), 78.5 (C-4), 76.8 (C-2), 72.7 (NAP*C*H_2_), 69.1 (C-6), 67.1 (C-3), 65.5 (C-5), 25.1 (S*C*H_2_CH_3_), 15.1 (SCH_2_*C*H_3_). HRMS ESI-TOF: calcd. for C_26_H_28_NaO_5_S [M + Na]^+^ 475.1550; found 475.1560.

### 3.5. Synthesis of Compound ***6***

Allose derivative **5** (780 mg, 7.653 mmol) was reacted with benzyl bromide (410 μL, 3.46 mmol) and sodium hydride (60%, 62 mg, 2.60 mmol) in DMF (8.0 mL) at 0 °C and stirred at room temperature for 1.5 h. The reaction was quenched by adding methanol (210 μL, 5.19 mmol) to the reaction solution at 0 °C. After diluting the reaction solution with ethyl acetate, it was washed successively with 1 M HCl, brine, saturated aq. NaHCO_3_, and brine. The organic layer was dried with Na_2_SO_4_ and the solvent was removed. The resulting residue was purified by silica gel column chromatography using a hexane–ethyl acetate (100/0 to 70/30, *v*/*v*) solution to afford allose donor **6** (928 mg, 1.71 mmol, 99%). *R_f_* = 0.51 (hexane/ethyl acetate = 3/1, *v*/*v*); [α]^24^_D_ − 82.09 (*c* 0.50, CHCl_3_); ^1^H-NMR (400 MHz, CDCl_3_) δ 7.84–7.29 (m, 17H, aromatic *H*), 5.43 (s, 1H, PhC*H*), 5.08 (d, 1H, *J* = 10.0 Hz, H-1), 4.93 (d, 1H, *J* = 11.6 Hz, PhC*H*_2_), 4.84–4.72 (m, 3H, NAPC*H*_2_, PhC*H*_2_), 4.35 (dd, 1H, *J* = 5.6, 10.4 Hz, H-6), 4.17 (brt, 1H, *J* = 2,4, 4.8 Hz, H-3), 4.12 (td, 1H, *J* = 5.2, 10.0 Hz, H-5), 3.68 (t, 1H, *J* = 10.4 Hz, H-6), 3.50 (dd, 1H, *J* = 1.6, 8.8 Hz, H-4), 3.40 (dd, 1H, *J* = 2.4, 9.6 Hz, H-2), 2.82–2.69 (m, 2H, SC*H*_2_CH_3_), 1.32 (t, 3H, *J* = 8.0 Hz, SCH_2_C*H*_3_). ^13^C-NMR (100 MHz, CDCl_3_) δ 138.6–126.0 (aromatic *C*), 101.9 (Ph*C*H), 82.5 (C-1), 79.8 (C-4), 77.6 (C-2), 74.0 (Ph*C*H_2_), 73.7 (C-3), 72.2 (NAP*C*H_2_), 69.3 (C-6), 66.1 (C-5), 24.9 (S*C*H_2_CH_3_), 15.0 (SCH_2_*C*H_3_). HRMS ESI-TOF: calcd. for C_33_H_34_NaO_5_S [M + Na]^+^ 565.2019; found 565.2019.

### 3.6. Synthesis of Compound ***7***

Allose derivative **5** (365 mg, 0.81 mmol) was reacted with imidazole (247 mg, 3.63 mmol) and chlorotrimethylsilane (203 μL, 1.61 mmol) in DMF (4.0 mL) at 0 °C and stirred at room temperature for 3 h. The reaction was quenched by adding methanol to the reaction solution at 0 °C. After diluting the reaction solution with ethyl acetate, it was washed successively with 1 M HCl, brine, saturated aq. NaHCO_3_, and brine. The organic layer was dried with Na_2_SO_4_ and the solvent was removed. The resulting residue was purified by silica gel column chromatography using a hexane–ethyl acetate (3/1, *v*/*v*) mixture to afford allose donor **7** (420 mg, 0.80 mmol, 99%). *R_f_* = 0.60 (hexane/ethyl acetate = 3/1, *v*/*v*); [α]^24^_D_ − 84.78 (*c* 1.00, CHCl_3_); ^1^H-NMR (400 MHz, CDCl_3_) δ 7.84–7.34 (m, 12H, aromatic *H*), 5.45 (s, 1H, PhC*H*), 5.02 (d, 1H, *J* = 9.6 Hz, H-1), 4.88–4.82 (m, 2H, NAPC*H*_2_), 4.40 (brt, 1H, *J* = 2.0 Hz, H-3), 4.32 (dd, 1H, *J* = 5.2, 10.0 Hz, H-6), 4.01 (dt, 1H, *J* = 5.2, 10.4 Hz, H-5), 3.67 (t, 1H, *J* = 10.4 Hz, H-6), 3.39 (dd, 1H, *J* = 2.0, 9.2 Hz, H-4), 3.34 (dd, 1H, *J* = 2.8, 10.0 Hz, H-2), 2.82–2.68 (m, 2H, SC*H*_2_CH_3_), 1.32 (t, 3H, *J* = 8.0 Hz, SCH_2_C*H*_3_), 0.14 (s, 9H, Si(C*H*_3_)_3_). ^13^C-NMR (100 MHz, CDCl_3_) δ 137.4–126.0 (aromatic *C*), 101.7 (Ph*C*H), 82.1 (C-1), 79.2 (C-4), 77.4 (C-2), 72.1 (NAPC*H*_2_), 69.2 (C-6), 68.3 (C-3), 65.5 (C-5), 24.9 (S*C*H_2_CH_3_), 15.0 (SCH_2_*C*H_3_), 0.654 (Si(*C*H_3_)_3_). HRMS ESI-TOF: calcd. for C_30_H_40_NaO_5_SSi [M + Na]^+^ 563.2258; found 563.2257.

### 3.7. IAD Reaction between ***6*** and ***8***

To a solution of allose derivative **6** (60 mg, 0.11 mmol) and 1,3,4,6-tetra-*O*-benzoyl-psicofuranoside acceptor **8** (44 mg, 0.07 mmol) in dichloromethane (3.0 mL) in the presence of 4 Å molecular sieves (300 mg), DDQ (30 mg, 0.13 mmol) was added at 0 °C and stirred at room temperature for 1 day. The ascorbic acid/citric acid/sodium hydroxide solution (0.7/1.3/0.9 wt%) was added at 0 °C to quench the reaction. The reaction mixture of tethering intermediate **9** obtained after post-treatment was dissolved in 1,2-dichloroethane (3.0 mL), and in the presence of 4 Å molecular sieves (300 mg), 1 M MeOTf solution (1.33 mL, 1.33 mmol) was added at 0 °C. After starting the reaction for 1 day, triethylamine (24 μL, 0.22 mmol) was added at 0 °C to quench the reaction. The reaction solution was diluted with ethyl acetate, filtered through celite, and washed with saturated aq. NaHCO_3_ and brine. The organic layer was dried over Na_2_SO_4_ and the solvent was removed under reduced pressure. The resulting residue was purified by gel filtration chromatography with chloroform to give allose derivative **10** (27 mg, 49%). *R_f_* = 0.50 (hexane/ethyl acetate = 3/2, *v*/*v*); [α]^24^_D_ 161.59 (*c* 0.30, CHCl_3_); ^1^H-NMR (400 MHz, CDCl_3_) δ 8.01–7.03 (m, 15H), 6.11 (s, 1H, PhC*H*), 5.63 (d, 1H, *J* = 4.8 Hz, H-1), 5.57 (s, 1H, NAPC*H*), 4.76 (dt, 1H, *J* = 5.6, 10.0 Hz, H-5), 4.56 (d, 1H, *J* = 12.4 Hz, PhC*H*_2_), 4.52 (dd, 1H, *J* = 5.2, 10.8 Hz, H-6), 4.44 (d, 1H, *J* = 12.4 Hz, PhC*H*_2_), 4.26 (t, 1H, *J* = 5.6 Hz, H-2), 4.07 (dd, 1H, *J* = 2.0, 4.8 Hz, H-3), 3.76 (t, 1H, *J* = 10.4 Hz, H-6), 3.66 (dd, *J* = 2.0, 9.6 Hz, H-4). ^13^C-NMR (100 MHz, CDCl_3_) δ 138.3–124.7 (aromatic C), 102.0 (Naph*C*H), 100.8 (Ph*C*H), 98.5 (C-1), 77.3–77.2 (C-4), 74.6 (Ph*C*H_2_), 72.5 (C-3), 71.1 (C-2), 69.4 (C-6), 59.4 (C-5). HRMS ESI-TOF: calcd. for C_31_H_28_NaO_6_ [M + Na]^+^ 519.1778; found 519.1782.

### 3.8. IAD Reaction between ***7*** and ***8***

To a solution of compound **7** (50 mg, 0.095 mmol), 1,3,4,6-tetra-*O*-benzoyl-psicofuranoside acceptor **8** (38 mg, 0.063 mmol), and dried MS (4 Å, 250 mg) in 1,2-dichloroethane (5.0 mL), DDQ (26 mg, 0.013 mmol) was added at 0 °C. The reaction mixture was stirred at room temperature for 19 h and then quenched with an ascorbate buffer [L-ascorbic acid (70 mg), citric acid monohydrate (120 mg), and NaOH (92 mg) in H_2_O (10.0 mL)] at 0 °C. The reaction mixture was diluted with ethyl acetate and filtered with celite. The filtrate was washed successively with saturated aq. NaHCO_3_ and brine. The organic layer was dried with MgSO_4_, filtered, and evaporated in vacuo to obtain intermediate **11**. To a solution of mixed acetal (**11**) and dried MS (4 Å, 500 mg) in 1,2-dichloroethane (5.0 mL), a solution of DTBMP (129 mg, 0.63 mmol) in 1,2-dichloroethane (5 mL) and 1 M MeOTf (1.3 mL, 1.33 mmol) was added under an argon atmosphere at 0 °C. The reaction mixture was stirred at 40 °C for 20 h and then quenched with triethylamine (185 μL, 2.00 mmol) at 0 °C. The reaction mixture was diluted with ethyl acetate and filtered through celite. The filtrate was washed with saturated aq. NaHCO_3_ and brine. The organic layer was dried over anhydrous MgSO_4_, filtered, and concentrated in vacuo. The residue was purified by preparative TLC using a toluene–ethyl acetate (8/1, *v*/*v*) mixture and preparative liquid chromatography (hexane/ethyl acetate = 85/15 to 77/23, *v*/*v*) to afford **12-1** (6.5 mg, 6.6 μmol, 10%) and **12-2** (5.8 mg, 5.9 μmol, 9%), respectively. Compound **12-1**: [α]^23^_D_ +45.95 (*c* 0.60, CHCl_3_); ^1^H-NMR (400 MHz, CDCl_3_) δ 8.03–7.19 (aromatic *H*), 6.08 (s, 1H, PhC*H*), 5.87 (dd, 1H, *J*_3,4_ = 5.6 Hz, *J*_4,5_ = 11.6 Hz, H-4^I^), 5.85 (d, 1H, *J*_3,4_ = 5.2 Hz, H-3^I^), 5.70 (d, 1H, *J*_1,2_ = 5.2 Hz, H-1^II^), 5.63 (s, 1H, NaphC*H*), 4.71–4.59 (m, 6H, H-5^I^, H-6^I^, H-6^I^, H-3^II^, H-5^II^, H-6^II^), 4.49 (t, 1H, *J*_1,2_ = 4.8 Hz, *J*_2,3_ = 5.6 Hz, H-2^II^), 4.41 (d, 1H, *J*_1,1_ = 12.4 Hz, H-1^I^), 4.11 (d, 1H, *J*_1,1_ = 12.4 Hz, H-1^I^), 3.96 (dd, 1H, *J*_3,4_ = *J*_4,5_ = 9.2 Hz, H-4^II^), 3.77 (q, 1H, *J*_5,6_ = 4.0 Hz, *J*_6,6_ = 12.4 Hz, H-6^II^). ^13^C-NMR (100 MHz, CDCl_3_) δ 166.1 (*C*O), 165.7 (*C*O), 165.0 (*C*O), 164.4 (*C*O), 137.0–124.2 (aromatic C), 107.1 (C-2^I^), 106.0 (Ph*C*H), 102.7 (Naph*C*H), 89.6 (C-1^II^), 80.0 (C-5^I^), 76.2 (C-4^II^), 75.7 (C-3^I^), 74.1 (C-3^II^), 73.6 (C-2^II^), 71.8 (C-4^I^), 69.3 (C-6^II^), 64.9 (C-6^I^), 63.3 (C-1^I^), 58.7 (C-5^II^). HRMS ESI-TOF: calcd. for C_58_H_48_NaO_15_ [M + Na]^+^ 1007.2885; found 1007.2886. Compound **12-2**: [α]^24^_D_ +144.51 (*c* 0.60, CHCl_3_); ^1^H-NMR (400 MHz, CDCl_3_) δ 8.25–6.98 (aromatic *H*), 6.11 (PhC*H*), 5.95 (dd, 1H, *J*_3,4_ = 2.4 Hz, *J*_4,5_ = 9.6 Hz, H-4^I^), 5.60 (s, 1H, PhC*H*), 5.49 (d, 1H, *J*_3,4_ = 2.4 Hz, H-3^I^), 5.42 (d, 1H, *J*_1,2_ = 5.26 Hz, H-1^II^), 5.03 (d, 1H, *J*_1,1_ = 17.6 Hz, H-1^I^), 4.81 (dd, 1H, *J*_5,6_ = 2.8 Hz, *J*_6,6_ = 12.4 Hz, H-6^I^), 4.67–4.65 (m, 2H, H-1^I^, H-3^II^), 4.61–4.51 (m, 2H, H-2^II^, H-5^II^), 4.44 (dd, 1H, *J*_5,6_ = 2.8 Hz, *J*_6,6_ = 12.4 Hz, H-6^I^), 4.41–4.35 (m, 2H, H-5^I^, H-6^II^), 3.98 (dd, 1H, *J*_3,4_ = 3.6 Hz, *J*_4,5_ = 9.6 Hz, H-4^II^), 3.69 (t, 1H, *J*_5,6_ = *J*_6,6_ = 10.4 Hz, H-6^II^). ^13^C-NMR (100 MHz, CDCl_3_) δ 166.4 (*C*O), 165.5 (*C*O), 164.7 (*C*O), 164.6 (*C*O), 136.9–126.4 (aromatic C), 106.7 (Ph*C*H), 102.7 (Naph*C*H), 98.8 (C-1^II^), 77.2 (C-5^I^), 76.2 (C-3^I^, C-4^II^), 74.7 (C-3^II^), 73.1 (C-2^II^), 70.3 (C-4^I^), 69.1 (C-6^II^), 67.7 (C-1^I^), 63.7 (C-6^I^), 57.5 (C-5^II^). HRMS ESI-TOF: calcd. for C_58_H_48_NaO_15_ [M + Na]^+^ 1007.2885; found 1007.2886.

### 3.9. Synthesis of Compound ***1***

To a solution of allose derivative **7** (300 mg, 0.57 mmol) and 1,3,4,6-tetra-*O*-benzoyl-psicofuranoside acceptor (**8**) (316 mg, 0.530 mmol) in 1,2-dichloroethane (20.0 mL) in the presence of 4 Å molecular sieves (1.58 g), DDQ (155 mg, 0.684 mmol) was added at 0 °C and stirred at room temperature for one day. The ascorbic acid/citric acid/sodium hydroxide solution (0.7/1.3/0.9 wt%) was added at 0 °C to quench the reaction. The reaction mixture of tethering intermediate **11** obtained after post-treatment was dissolved in 1,2-dichloroethane (30.0 mL), and in the presence of 4 Å molecular sieves (1.58 g), 1 M MeOTf solution (1.33 mL, 1.33 mmol) was added at 0 °C. After the reaction proceeded for one day at 40 °C, triethylamine (185 μL, 2.00 mmol) was added at 0 °C to quench the reaction. The reaction solution was diluted with ethyl acetate, filtered through celite, and washed with saturated aq. NaHCO_3_ and brine. The organic layer was dried over Na_2_SO_4_ and the solvent was removed under reduced pressure to obtain the mixture **12** (259 mg). To a solution of the mixture (234 mg) in dry dichloromethane (2.0 mL), TFA (200 μL) was added at 0 °C, and the entire mixture was stirred for 1 min. The reaction mixture was dried with N_2_ gas, and pyridine (200 μL) was added at 0 °C. The reaction mixture was azeotropically dried with toluene, diluted with chloroform, and washed successively with saturated aq. NaHCO_3_ and brine. The organic layer was dried over anhydrous MgSO_4_, filtered, and concentrated in vacuo. The resulting residue was purified by gel filtration chromatography using chloroform and by flash silica gel column chromatography using a chloroform–MeOH (15/1, *v/v*) mixture to give an intermediate compound (18.7 mg, 4%). To a solution of the intermediate compound (18.7 mg, 0.025 mmol) in MeOH (1.5 mL), 1 M NaOMe in MeOH (20 µL) was added at 0 °C. The reaction mixture was stirred at room temperature for 4 h, neutralized with Amberlyst, filtered, and concentrated in vacuo. The resulting residue was purified by reversed-phase column chromatography (H_2_O) to give compound **1** (8.6 mg, 0.025 mmol, quant.). [α]^23^_D_ +120.24 (*c* 0.10, H_2_O); ^1^H-NMR (400 MHz, D_2_O) δ 7.70–7.27 (m, 5H, aromatic *H* of benzoate adduct), 5.32 (d, 1H, *J* = 4.0 Hz, H-1^II^), 4.25 (dd, 1H, *J* = 4.4, 8.8 Hz), 4.20 (d, 1H, *J* = 4.8 Hz), 4.09 (t, 1H, *J* = 4.8 Hz), 4.04 (td, 1H, *J* = 5.2, 8.4 Hz), 3.87 (m, 1H), 3.81–3.42 (m, 8H). ^13^C-NMR (100 MHz, D_2_O) δ 175.8 (*C*=O of benzoate adduct), 136.1–128.3 (aromatic *C* of benzoate adduct), 109.2 (C-2^I^), 91.6 (C-1^II^), 83.5 (C-5^I^), 73.5 (C-3^I^), 71.0 (C-3^II^), 70.4 (C-4^I^), 67.5 (C-5^II^), 66.7 (C-2^II^), 65.6 (C-4^II^), 62.4 (C-6^I^), 60.2 (C-1^I^), 59.8 (C-6^II^). HRMS ESI-TOF: calcd. for C_12_H_22_NaO_11_ [M + Na]^+^ 365.1054; found 365.1046.

## 4. Conclusions

In this study, we synthesized a sucrose-type disaccharide composed of the rare sugars D-allose and D-psicose. We confirmed that the α-stereoselective allosylation at the anomeric position of 1,3,4,6-tetra-*O*-benzoyl psicofuranose proceeded by NAP-IAD glycosylation. The desired disaccharide was obtained after deprotection and is expected to be a novel functional sweetener. However, the glycosyl linkage between allose and psicose is sensitive to acid, and judicious choice of the protecting group is required, which might be challenging. Structural determination of the ionic adduct will be the subject of future work. The development of alkaline isomerization methods and practical isomerization enzymes has enabled the mass production of rare sugars. As a result, syrups containing D-psicose are now available in the market. Several studies have reported the chemical derivatization of rare sugars, whereas few oligomerizations and multifunctionalizations of rare sugars have been investigated. The chemical synthesis of rare sugar-containing molecules will allow the screening of potential artificial sweeteners and provide insights into targets for enzymatic synthesis.

## Figures and Tables

**Table 1:** ^1^H and ^13^C chemical shifts of α-D-allopyranosyl-(1→2)-β (or α)-D-psicofuranoside.

	α-D-All*p*-(1→2)-β-D-Psi*f* (1)		α-D-All*p*-(1→2)-α-D-Psi*f*	
	^1^H/ppm	^13^C/ppm	^1^H/ppm	^13^C/ppm
All				
1	5.32 (d, *J* = 4.0 Hz)	91.6	5.36 (d, *J* = 4.0 Hz)	90.4
2	3.79–3.76	66.7	3.77 (t, *J* = 3.6 Hz)	66.6
3	4.09 (t, *J* = 6.4 Hz)	71.0	4.09 (t, *J* = 6.8 Hz)	70.8
4	3.65–3.61	65.6	3.58 (dd, *J* = 2.8, 10.8 Hz)	66.0
5	3.87 (m)	67.5	3.88 (td, *J* = 2.5, 5.6, 10.4 Hz)	67.5
6	3.81–3.76, 3.65–3.61	59.8	3.83–3.82, 3.73–3.63	60.2
Psi				
1	3.81–3.76, 3.65–3.61	60.2	3.91–3.86, 3.73–3.63	60.7
2	-	109.2	-	106.2
3	4.20 (d, *J* = 4.8 Hz)	73.5	4.48 (d, *J* = 6.4 Hz)	70.8
4	4.25 (dd, *J* = 4.4, 8.8 Hz)	70.4	4.05 (dd, *J* = 1.2, 6.0 Hz)	70.7
5	4.04 (td, *J* = 5.2, 8.4 Hz)	83.5	4.21–4.19	85.4
6	3.81–3.74	62.4	4.21–4.19, 3.73–3.63	61.6

Chemical shifts are referenced to the internal HOD (δ 4.80 ppm) for ^1^H NMR and a native scale for ^13^C NMR.

## Data Availability

Data are contained within the article and Supplementary Materials.

## Supplementary Materials

The following supporting information can be downloaded at: https://www.mdpi.com/article/10.3390/molecules29081771/s1, Scheme S1: Synthesis of the stereoisomers of D-allopyranosyl-D-psicofuranoside derivatives S2 by the conventional glycosylation method); Scheme S2: Deprotection of α-D-allopyranosyl-(1→2)-α-D-psicofuranoside derivative; Table S1: ^13^C-chemical shifts (ppm) and ^1^*J*_C1–H1_ coupling constants (Hz) for stereoisomers of α/β-D-allopyranosyl-(1→2)-α/β-D-psicofuranoside derivatives (CDCl_3_).
