# Supplementary material for: Synthesis of Sucrose-Mimicking Disaccharide by Intramolecular Aglycone Delivery
Source: Molecules. 2024 Apr 13;29(8):1771. doi: 10.3390/molecules29081771 (PMC11051705; doi:10.3390/molecules29081771)
Supplement: Supplementary file 1 [file molecules-29-01771-s001.zip › molecules-2887917-supplementary.pdf]

Supporting Information

Synthesis of Sucrose-Mimicking Disaccharide by Intramolecular Aglycone Delivery

Kanae Sano [1], Akihiro Ishiwata [2,*], Hiroto Takamori [1], Takashi Kikuma [1], Katsunori Tanaka [2,3], Yukishige Ito [2,4] and Yoichi Takeda [1,*]

[1] Department of Biotechnology, College of Life Sciences, Ritsumeikan University, Kusatsu 525-8577, Japan; ksano@gst.ritsumei.ac.jp (K.S.); tkikuma@fc.ritsumei.ac.jp (T.K.)

[2] RIKEN Cluster for Pioneering Research, Wako 351-0198, Japan; kotzenori@riken.jp (K.T.); yukito@chem.sci.osaka-u.ac.jp (Y.I.)

[3] Department of Chemical Science and Engineering, Tokyo Institute of Technology, Tokyo 152-8552, Japan

[4] Graduate School of Science, Osaka University, Toyonaka 560-0043, Japan

* Correspondence: aishiwa@riken.jp (A.I.); yotakeda@fc.ritsumei.ac.jp (Y.T.)

Contents

1. Synthesis of allose donor **S1**…........…........…........…........…..........…………................... 2

2. Synthesis of the stereoisomer of D-allosyl-D-psicofuranoside derivatives………………... 3

3. ^{1}H NMR and ^{13}C NMR spectra of compounds... 7

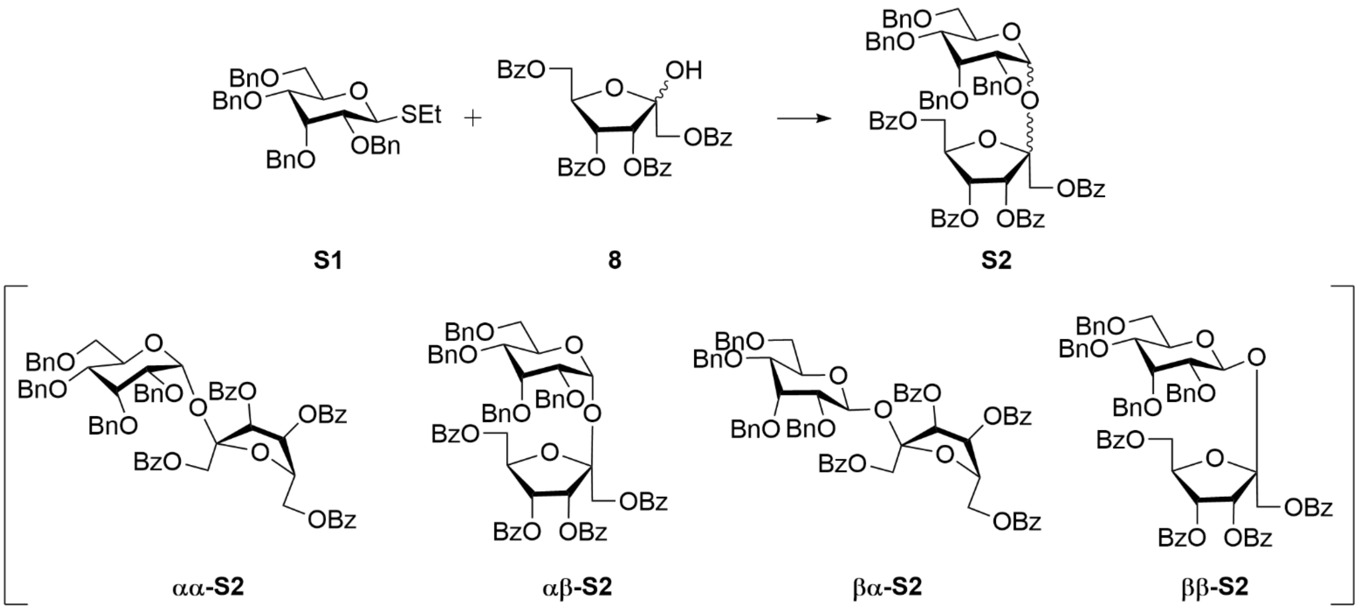

Scheme S1. Synthesis of the stereoisomers of D-allosyl-D-psicofuranoside derivatives **S2** by the conventional glycosylation method.

Synthesis of allose donor S1

To a solution of compound **3** (1.7 g, 7.65 mmol) and benzyl bromide (7.3 mL, 61.2 mmol) in DMF (40 mL), 60% NaH (1.8 g, 45.9 mmol) was added at 0 °C. The reaction mixture was stirred at room temperature for 12 hours. The reaction was quenched by adding MeOH (excess) at 0 °C. After diluting the reaction mixture with ethyl acetate, it was washed with 1M HCl, brine, saturated aq. $NaHCO_3$, and brine, successively. The organic layer was dried with Na_2SO_4, filtered, and the solvent was removed. The resulting residue was purified by flash silica gel column chromatography using hexane–ethyl acetate (5/1, *v/v*) to afford compound **S1** (1.56 g, 21.4 mmol, 35%). R_f = 0.53 (hexane/ethyl acetate = 3/1, *v/v*); $[\alpha]^{24}_{D}$ –6.20 (*c* 1.00, $CHCl_3$),^{1}H-NMR (400 MHz, $CDCl_3$) δ 7.38–7.25 (m, 20H, aromatic *H*), 5.02 (d, 1H, *J* = 10.4 Hz, H-1), 4.84 (d, 1H, *J* = 12.4 Hz, CH_2), 4.78 (d, 1H, *J* = 12.0 Hz, CH_2), 4.57 (m, 6H, CH_2), 4.40 (d, 1H, *J* = 12.0 Hz, CH_2), 4.11 (t, 1H, *J* = 2.4 Hz, H-3), 4.02 (ddd, 1H, *J* = 1.6 Hz, *J* = 4.4 Hz, *J* = 6.4 Hz, *J* = 10.0 Hz, H-5), 3.75 (dd, 1H, *J* = 2.0 Hz, *J* = 11.2 Hz, H-6), 3.67 (dd, 1H, *J* = 4.4 Hz, *J* = 11.2 Hz, H-6), 3.45 (dd, 1H, *J* = 2.4 Hz, *J* = 9.6 Hz, H-4), 3.27 (dd, 1H, *J* = 2.8 Hz, *J* =10.0 Hz, H-2), 2.73 (m, 2H, SCH_2CH_3), 1.31 (t, 3H, *J* = 7.6 Hz, SCH_2CH_3).^{13}C-NMR (100 MHz, $CDCl_3$) δ 138.9–127.7 (aromatic *C*), 81.5 (C-1), 78.7 (C-2), 75.6 (C-4), 74.9 (C-5), 74.2 (CH_2), 73.3 (C-3, CH_2), 72.3 (CH_2), 71.5 (CH_2), 69.4 (C-6), 24.7 (SCH_2CH_3), 15.1 (SCH_2CH_3). HRMS ESI-TOF: calcd. for $C_{36}H_{40}NaO_5S$ $[M+Na]^+$ 607.2489; found 607.2490.

Synthesis of the stereoisomer of D-allosyl-D-psicofuranoside derivatives

To a solution of glycosyl donor **S1** (53 mg, 0.091 mmol), glycosyl acceptor (50 mg, 0.083 mmol), in CH_2Cl_2 (4.0 mL) in the presence of NIS (28 mg, 0.125 mmol) and MS4Å (300 mg) was added TfOH (1.5 µL, 0.017 mmol) and stirred at –40 °C for 1.5 hours. The triethylamine (12 µL, 0.083 mmol) was added at –40 °C. The reaction mixture was diluted with ethyl acetate, it was washed with 1 M HCl, brine, saturated aq. $NaHCO_3$ and brine, in that order. The organic layer was dried with $MgSO_4$, and the solvent was removed. The resulting residue was purified by gel filtration chromatography with chloroform. The di-saccharide fraction was purified by flash silica gel column chromatography with toluene–ethyl acetate (9/1, *v/v*) to afford compounds αα-**S2** (αAll-αPsi, 29 mg, 0.026 mmol, 31%) and mixture of αβ-**S2** (αAll-βPsi, 6.5 mg, 0.006 mmol, 7%), βα-**S2** (βAll-αPsi, 21 mg, 0.019 mmol, 22%) and ββ-**S2** (βAll-βPsi, 4.9 mg, 0.004 mmol, 5%). The yields of αβ-**S2**, βα-**S2**, and ββ-**S2** were calculated from the amount of mixture and the integral value of the NMR spectra.

αα-**S2**; ^{1}H-NMR (400 MHz, $CDCl_3$) δ 8.06–7.08 (aromatic *H*), 5.78 (d, 1H, *J* = 8.4 Hz, H-3^{I}), 5.72 (d, 1H, *J* = 4.0 Hz, H-1^{II}), 5.66 (dd, 1H, *J* = 6.0, 7.2 Hz, H-4^{I}), 4.94 (broad dd, *J* = 4.6, 8.2 Hz, H-5^{I}), 4.83 (d, 1H, *J* = 12.4 Hz, H-1^{I}), 4.77 (d, 1H, *J* = 13.6 Hz, CH_2), 4.70 (m, 3H, H-1^{I}, H-6^{I}, CH_2), 4.53 (d, 1H, *J* = 12.4 Hz, H-6^{I}), 4.56 (d, 2H, *J* = 13.6 Hz, CH_2, CH_2), 4.39 (d, 1H, *J* = 13.6 Hz, CH_2), 4.22 (broad s, 1H, H-3^{II}), 3.66 (dd, 1H, *J* = 2.0, 10.8 Hz, H-6^{II}), 3.57 (dd, 1H, *J* = 1.6, 10.0 Hz, H-4^{II}), 3.53 (d, 1H, *J* = 3.6 Hz, H-2^{II}), 3.48 (broad d, 1H, *J* = 10.4 Hz, H-6^{II}). ^{13}C-NMR (100 MHz, $CDCl_3$) δ 166.1 (*C*O), 165.9 (*C*O), 165.6 (*C*O), 165.5 (*C*O), 139.7–127.4 (aromatic *C*), 104.1 (C-2^{II}), 90.4 (C-1^{II}), 79.4 (C-5^{I}), 76.9 (C-2^{II}), 74.6 (C-4^{II}), 73.5 (Ph*C*H_2), 72.2 (Ph*C*H_2, C-3^{I}) 71.6 (Ph*C*H_2), 71.0 (C-3^{II}, Ph*C*H_2), 70.1 (C-4^{I}), 68.5 (C-6^{II}), 67.3 (C-5^{II}), 64.7 (C-1^{I}), 63.4 (C-6^{I}).

αβ-**S2**; ^{1}H-NMR (400 MHz, $CDCl_3$) δ 8.13–6.97 (aromatic *H*), 6.24 (d, 1H, *J* = 5.2 Hz, H-3^{I}), 5.95 (dd, 1H, *J* = 5.2, 6.4 Hz, H-4^{I}), 5.58 (d, 1H, *J* = 3.6 Hz, H-1^{II}), 4.95 (d, 1H, *J* = 12.0 Hz, CH_2), 4.81–4.67 (m, 3H, H-5^{I}, H-6^{I}, H-6^{I}), 4.14 (t, 1H, *J* = 2.4 Hz, H-3^{II}), 3.80 (dd, 1H, *J* = 3.2, 10.4 Hz, H-6^{II}), 3.76 (dd, *J* = 2.0, 10.4 Hz, H-6^{II}), 3.53 (dd, 1H, *J* = 1.6, 10.4 Hz, H-4^{II}), 3.36 (dd, 1H, *J* = 3.6, 4.8 Hz, H-2^{II}). ^{13}C-NMR (100 MHz, $CDCl_3$) δ 166.0-164.5 (*C*O), 138.7–126.8 (aromatic *C*), 107.3 (C-2^{I}), 90.9 (C-1^{II}), 79.7 (C-5^{I}), 75.6 (C-2^{II}), 74.9 (C-3^{I}), 74.4 (C-4^{II}), 74.3–72.8 (Ph*C*H_2, Ph*C*H_2, Ph*C*H_2), 72.2 (C-4^{I}), 71.0 (C-3^{II}), 70.8 (Ph*C*H_2), 67.6 (C-5^{II}), 65.5 (C-1^{I}), 62.8 (C-6^{I}).

βα-**S2;** ^{1}H-NMR (400 MHz, $CDCl_3$) δ 8.13–6.97 (aromatic *H*), 5.81 (d, 1H, *J* = 6.4 Hz, H-3^{I}), 5.79 (dd, 1H, *J* = 2.8, 6.4 Hz, H-4^{I}), 5.53 (d, 1H, *J* = 8.4 Hz, H-1^{II}), 5.19 (broad q, 1H, *J* = 2.8, 5.6 Hz, H-5^{I}), 4.72–4.27 (m, 4H, H-1^{I}, H-1^{I}, H-6^{I}, H-6^{I}), 4.16 (ddd, 1H, *J* =1.6, 6.4, 8.0, 9.6 Hz, H-5^{II}), 4.05 (broad t, 1H, *J* = 2.4 Hz, H-3^{II}), 3.62 (dd, 1H, *J* = 1.6, 10.4 Hz, H-6^{II}), 3.28 (dd, 1H, *J* = 6.4, 10.8 Hz, H-6^{II}), 3.27–3.22 (m, 2H, H-2^{II}, H-4^{II}). ^{13}C-NMR (100 MHz, $CDCl_3$) δ 166.0–164.5 (*C*O), 138.7–126.8 (aromatic *C*), 104.2 (C-2^{I}), 94.8 (C-1^{II}), 81.6 (C-5^{I}), 78.4 (C-2^{II}), 75.5 (C-4^{II}), 74.7 (C-3^{II}), 74.8 (C-3^{I}), 74.3–72.8 (Ph*C*H_2, Ph*C*H_2, Ph*C*H_2), 72.0 (C-5^{II}), 71.2 (Ph*C*H_2), 71.1 (C-4^{I}), 69.9 (C-6^{II}), 64.3 (C-1^{I}), 62.9 (C-6^{I}).

ββ-**S2**; ^{1}H-NMR (400 MHz, $CDCl_3$) δ 8.00–7.00 (aromatic *H*), 6.13 (d, 1H, *J* = 4.4 Hz, H-3^{I}), 5.96 (dd, *J* = 4.8 Hz, 8.8 Hz, H-4^{I}), 5.56 (d, *J* = 8.0 Hz, H-1^{II}), 4.86–4.37 (m, 4H, H-1^{I}, H-1^{I}, H-5^{I}, H-6^{I}), 4.08 (s, 1H, H-3^{II}), 4.02 (ddd, 1H, *J* = 2.0, 5.0, 10.2 Hz, H-5^{II}), 3.70 (dd, 1H, *J* = 2.0, 11.2 Hz, H-6^{II}), 3.57–3.53 (m, 2H, H-4^{II}, H-6^{II}), 3.38 (dd, *J* = 2.4, 8.4 Hz, H-2^{II}). ^{13}C-NMR (100 MHz, $CDCl_3$) δ 165.9 (*C*O), 165.7 (*C*O), 165.1 (*C*O), 164.6 (*C*O), 139.1–127.2 (aromatic *C*), 107.7 (C-2^{I}), 92.5 (C-1^{II}), 79.1 (C-5^{I}), 78.8 (C-2^{II}), 74.8 (C-3^{II}, C-4^{II}), 74.6 (C-3^{I}, Ph*C*H_2), 73.4 (C-4^{I}, Ph*C*H_2), 72.9 (C-5^{II}), 72.6 (Ph*C*H_2), 71.8 (Ph*C*H_2), 68.8 (C-6^{II}), 65.5 (C-1^{I}), 62.6 (C-6^{I}).

Table S1. ^{13}C-chemical shifts (ppm) and $^{1}J_{C1-H1}$ coupling constants (Hz) for stereoisomers of α/β-D-allopyranosyl-(1→2)-α/β-D-psicofuranoside derivatives ($CDCl_3$).

	αα-**S2**	αβ-**S2**	βα-**S2**	ββ-**S2**
δ_C D-allo 1	90.4	90.9	94.8	92.5
2	76.9	75.6	78.4	78.8
3	71.0	71.0	74.7	74.8
4	74.6	74.4	75.5	74.8
5	67.3	67.6	72.0	72.9
6	68.5	68.7	69.9	68.8
D-psico 1	64.7	65.5	64.3	65.5
2	104.1	107.3	104.2	107.7
3	72.2	74.9	74.8	74.6
4	70.1	72.2	71.1	73.4
5	79.4	79.7	81.6	79.1
6	63.4	62.8	62.9	62.6
J_{C1-H1} (allo)	177.0	170.0	172.0	172–174

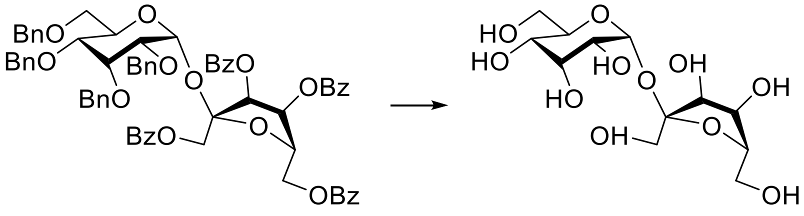

Scheme S2. Deprotection of α-D-allosyl-(1→2)-α-D-psicofuranoside derivative.

To a solution of the αα-**S2** (29 mg, 0.026 mmol) in MeOH (1.0 mL) and THF (1.0 mL) was added 1 M NaOMe in MeOH (26 µL) at 0 °C. The reaction mixture was stirred at room temperature for 12 hours, and neutralized with Amberlyst, filtered, and concentrated *in vacuo*. The resulting residue was purified by flash silica gel column chromatography with chloroform–MeOH (85/15, *v/v*) to afford intermediate (6.9 mg, 0.019 mmol, 73%). To a solution of the intermediate in H_2O (5.0 mL) was stirred in the presence of $Pd(OH)_2/C$ (15 mg) at 40 °C under H_2 atmosphere for 1 day. The reaction mixture was filtered through celite. The filtrate was lyophilized. The residue was purified by C18 reversed-phase column chromatography (H_2O) to give deprotected product (6.9 mg, 0.019 mmol, 73%). ^{1}H-NMR (400 MHz, $CDCl_3$) δ 5.36 (d, 1H, J = 4.0 Hz, H-1^{II}), 4.48 (d, 1H, J = 6.4 Hz, H-3^{I}), 4.21–4.19 (m, 1H, H-5^{I}), 4.09 (t, 1H, J = 6.8 Hz, H-3^{II}), 4.05 (dd, 1H, J = 1.2, 6.0 Hz, H-4^{I}), 3.88 (td, 1H, J = 2.5, 5.6, 10.4 Hz, H-5^{II}), 3.91–3.82 (m, 2H, H-1^{I}, H-6^{II}), 3.77 (t, 1H, J = 3.6 Hz, H-2^{II}), 3.73–3.63 (m, 3H, H-1^{I}, H-6^{I}, H-6^{II}), 3.58 (dd, 1H, J = 2.8, 10.8 Hz, H-4^{II}). ^{13}C-NMR (100 MHz, $CDCl_3$) δ 106.2 (C-2^{I}), 90.4 (C-1^{I}), 85.4 (C-5^{I}), 70.8 (C-3^{I}, C-3^{II}) 70.7 (C-4^{I}), 67.5 (C-5^{II}), 66.6 (C-2^{II}), 66.0 (C-4^{II}), 61.6 (C-6^{I}), 60.7 (C-1^{I}), 60.2 (C-6^{II}).

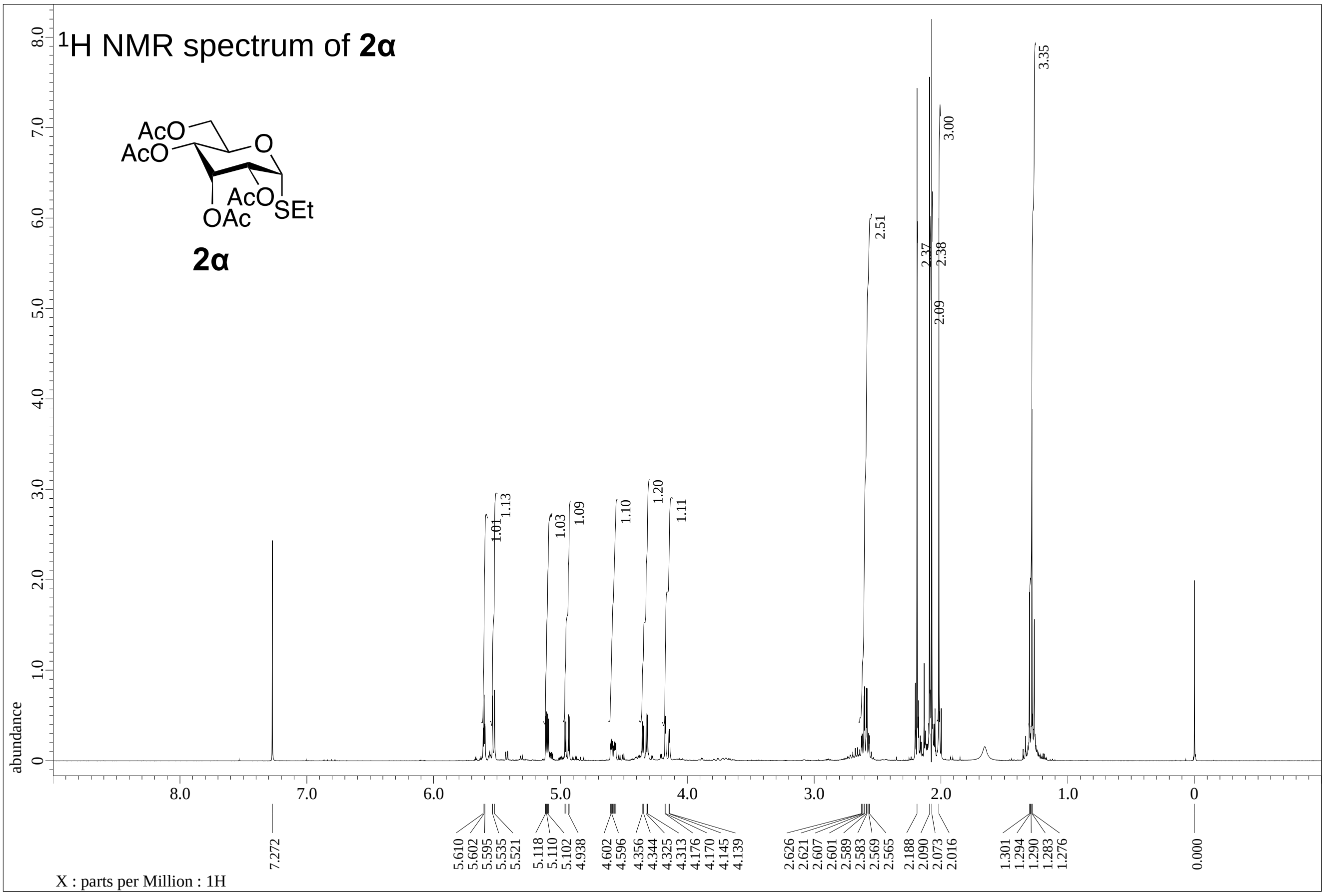

^{1}H NMR spectrum of **2α**
AcO
AcO
O
AcO
SEt
OAc
2α
abundance
X : parts per Million : 1H

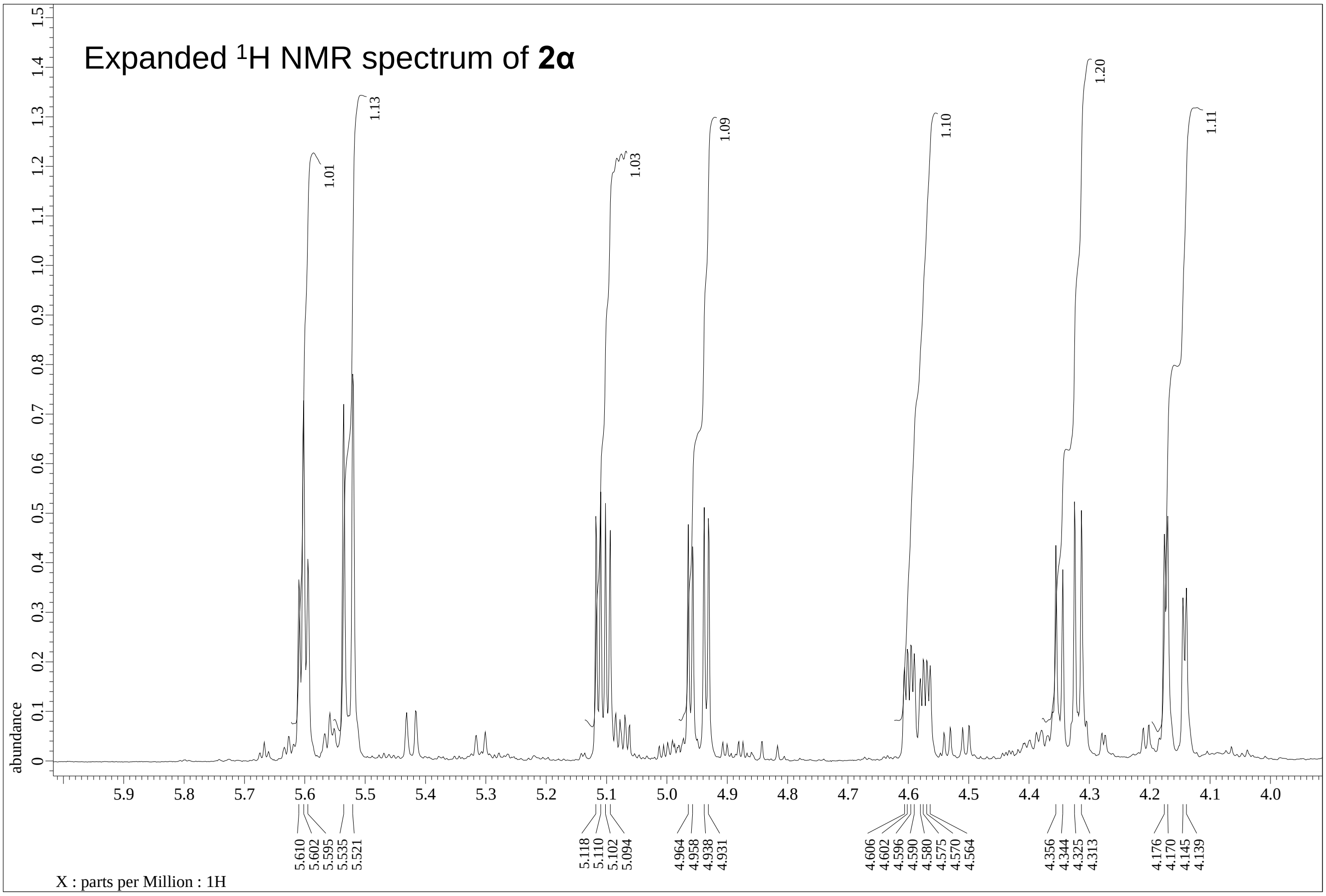

Expanded ^{1}H NMR spectrum of **2α**
abundance
X : parts per Million : 1H

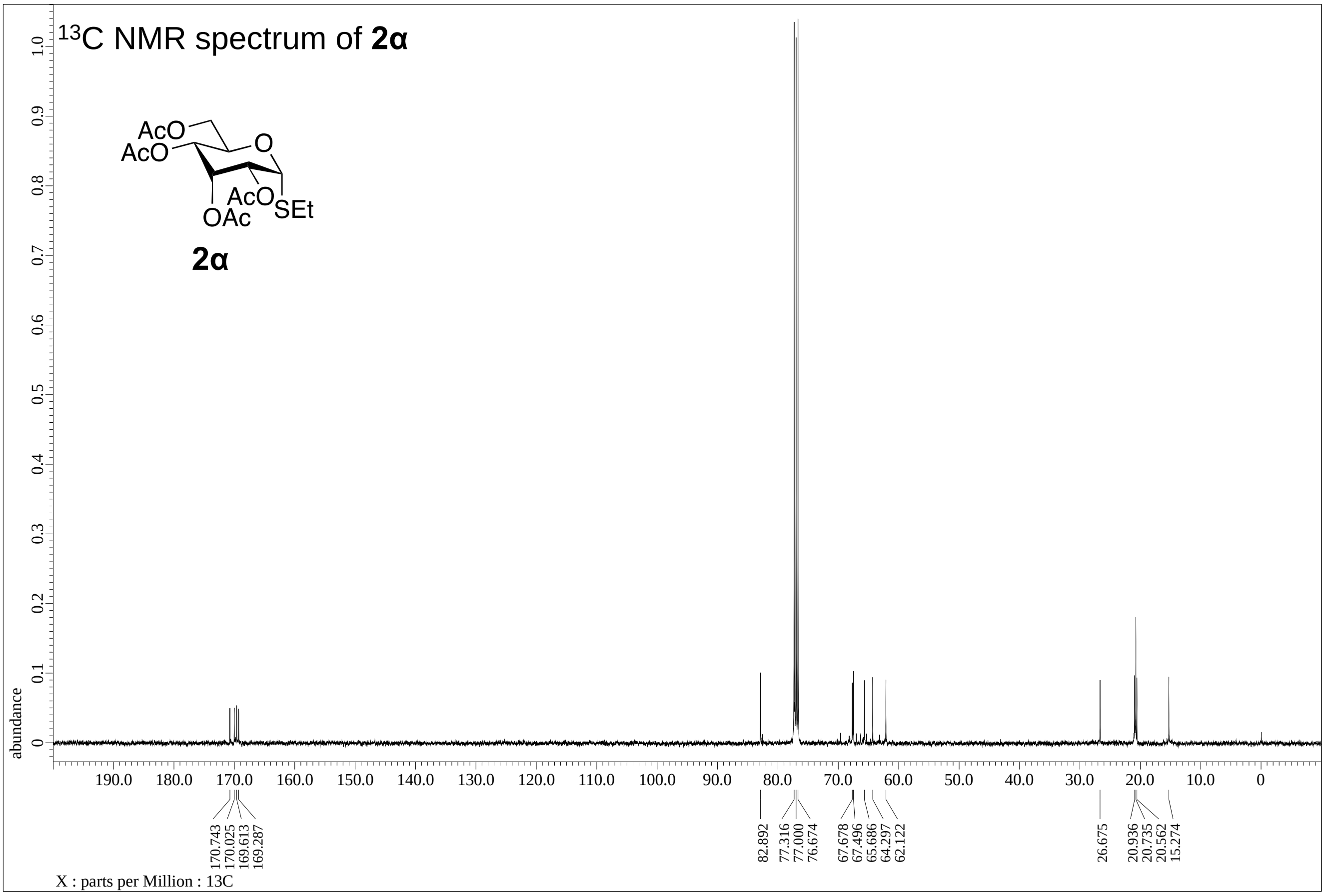

13C NMR spectrum of 2α
AcO
AcO
O
AcO
OAc
SEt
2α
abundance
X : parts per Million : 13C
170.743
170.025
169.613
169.287
82.892
77.316
77.000
76.674
67.678
67.496
65.686
64.297
62.122
26.675
20.936
20.735
20.562
15.274

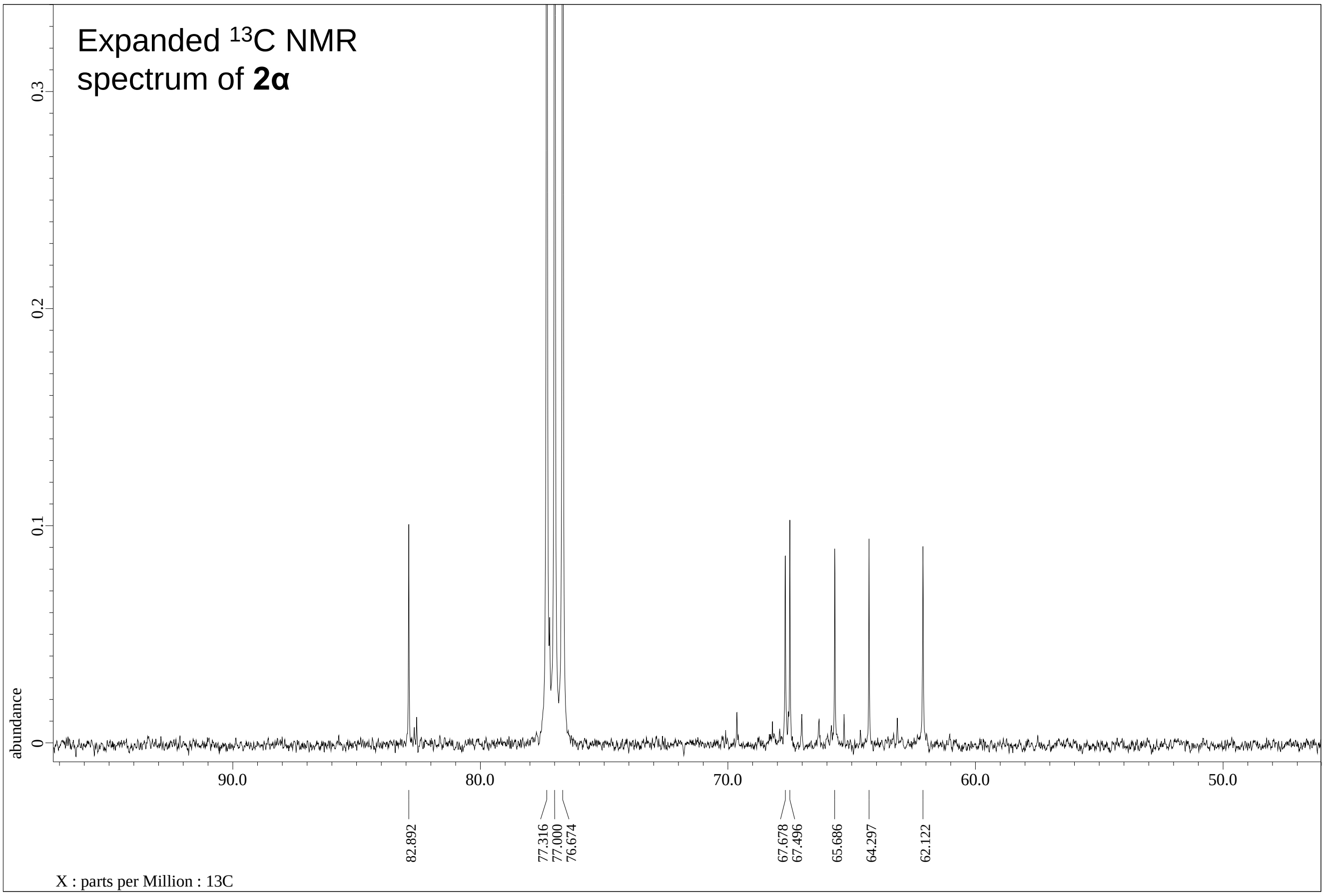

Expanded 13C NMR spectrum of 2α
abundance
X : parts per Million : 13C
82.892
77.316
77.000
76.674
67.678
67.496
65.686
64.297
62.122

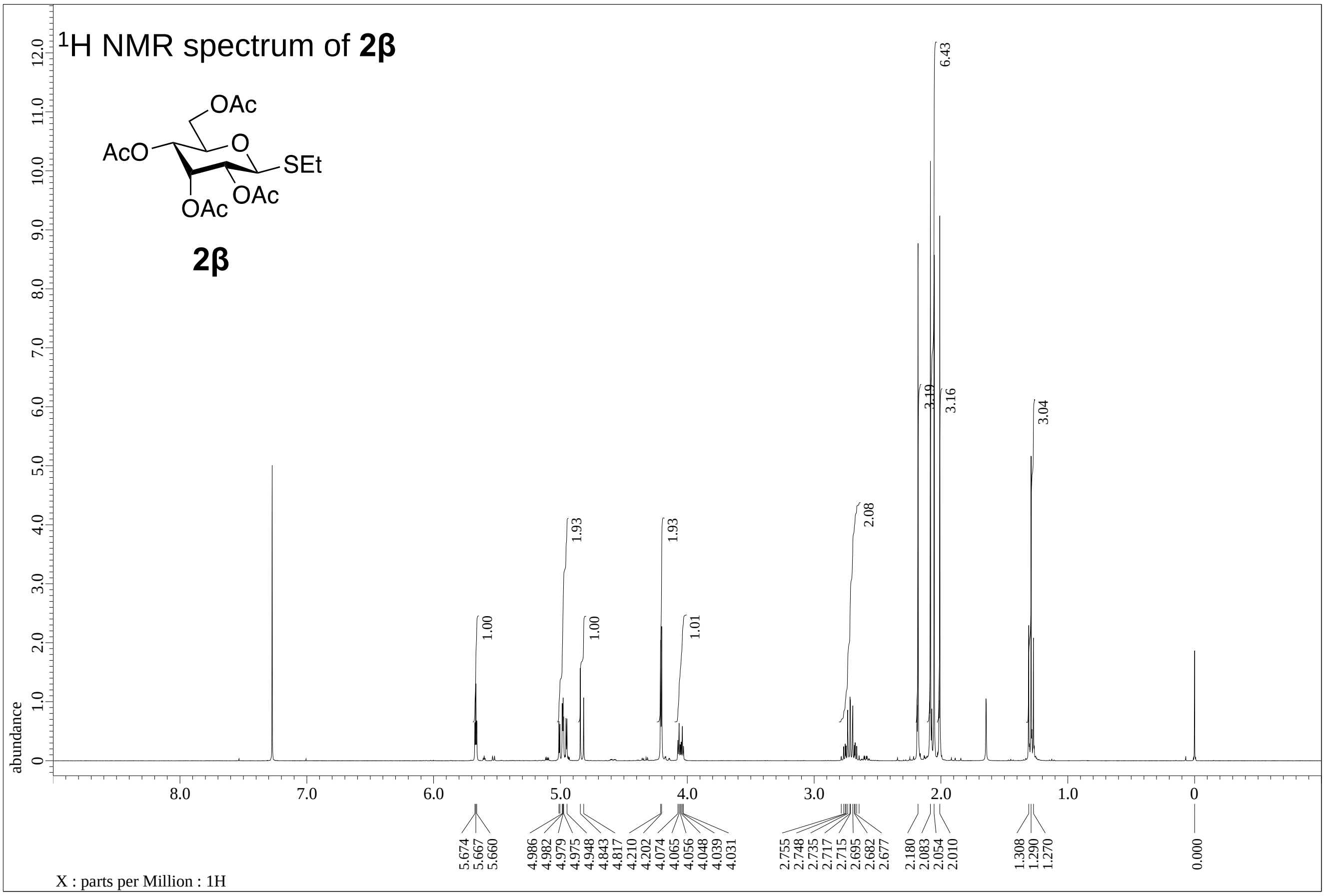

1H NMR spectrum of 2β
OAc
AcO
O
SEt
OAc
OAc
2β
abundance
X : parts per Million : 1H

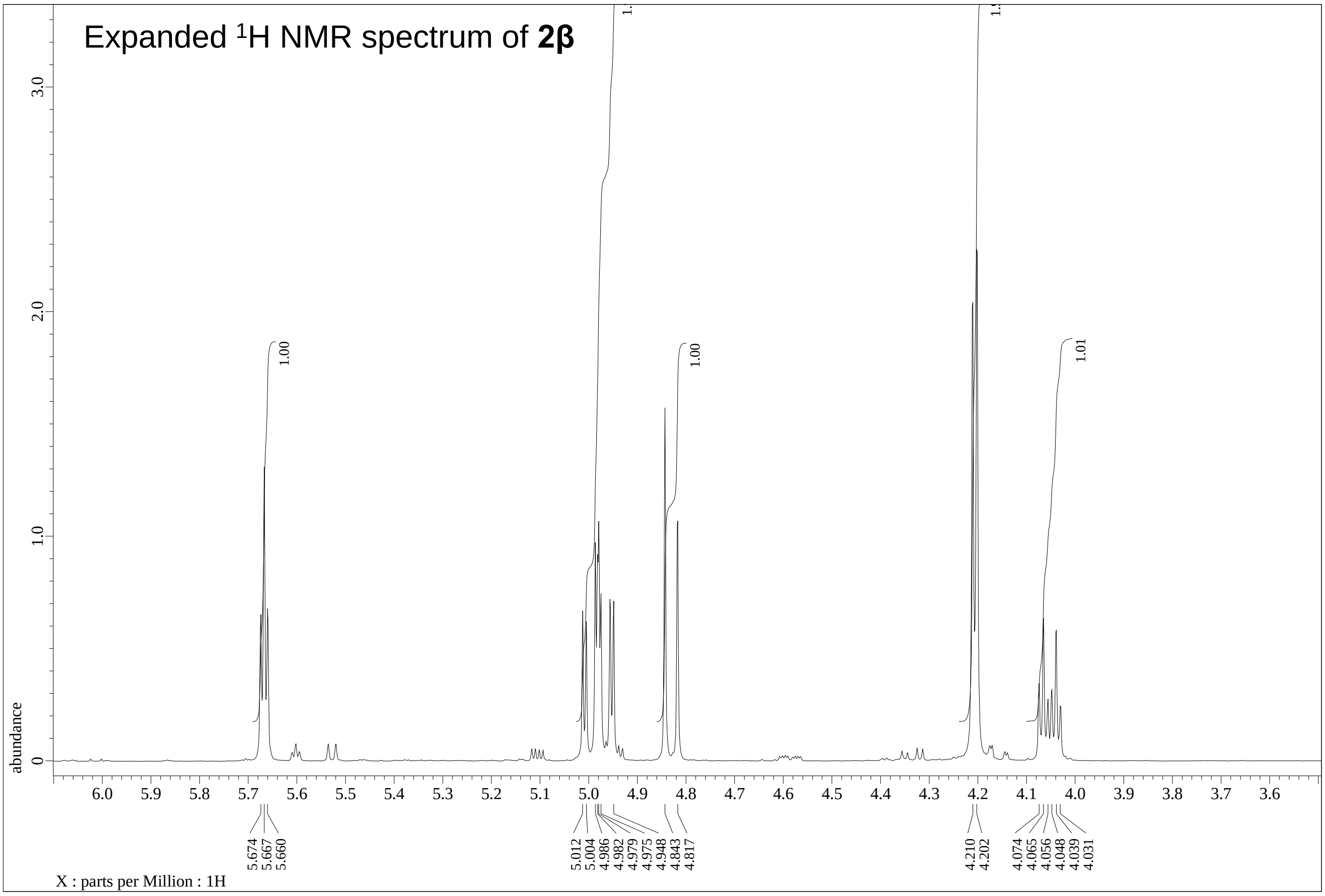

Expanded 1H NMR spectrum of 2β
abundance
X : parts per Million : 1H

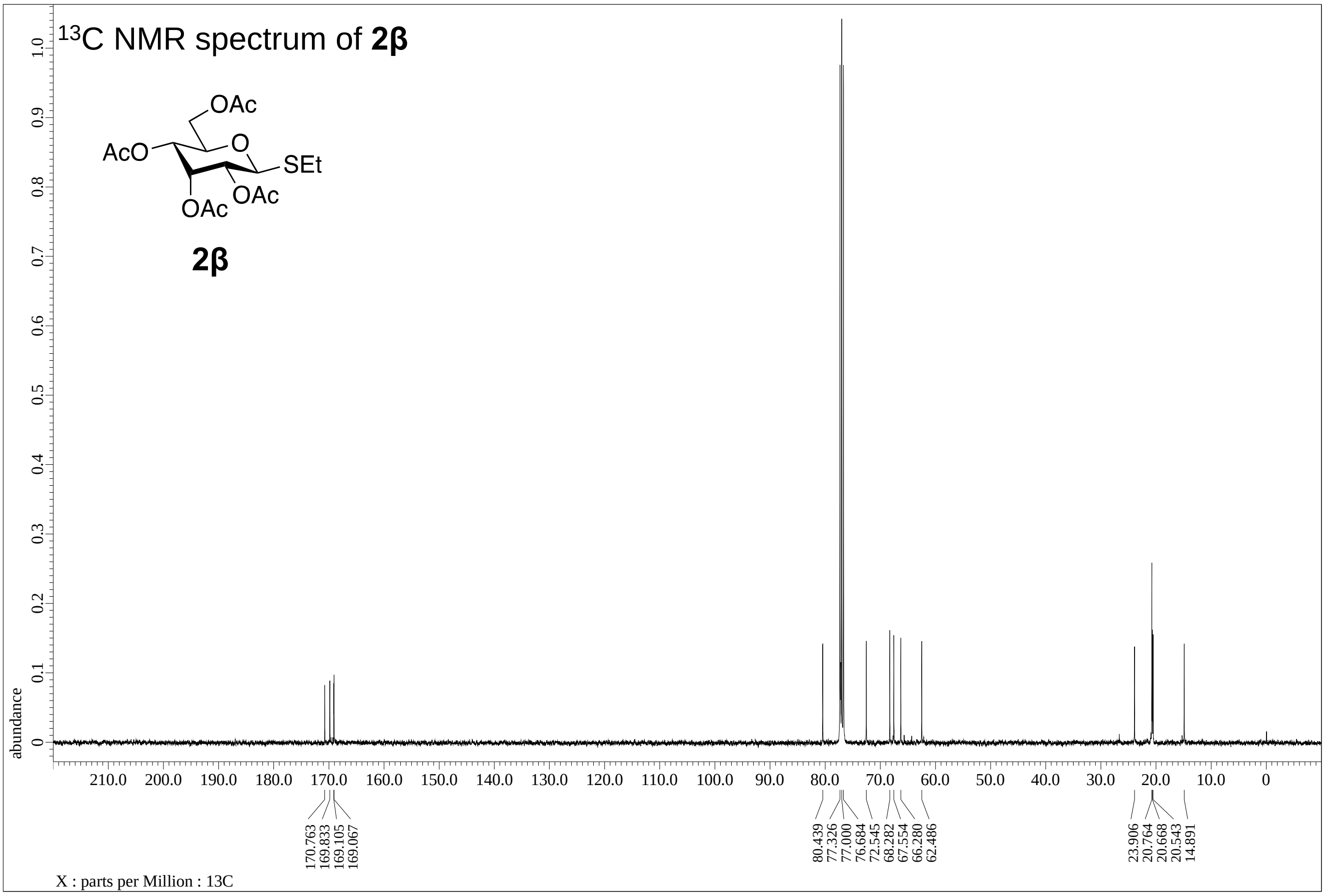

13C NMR spectrum of 2β
OAc
AcO
O
SEt
OAc
OAc
2β
abundance
170.763
169.833
169.105
169.067
80.439
77.326
77.000
76.684
72.545
68.282
67.554
66.280
62.486
23.906
20.764
20.668
20.543
14.891
X : parts per Million : 13C

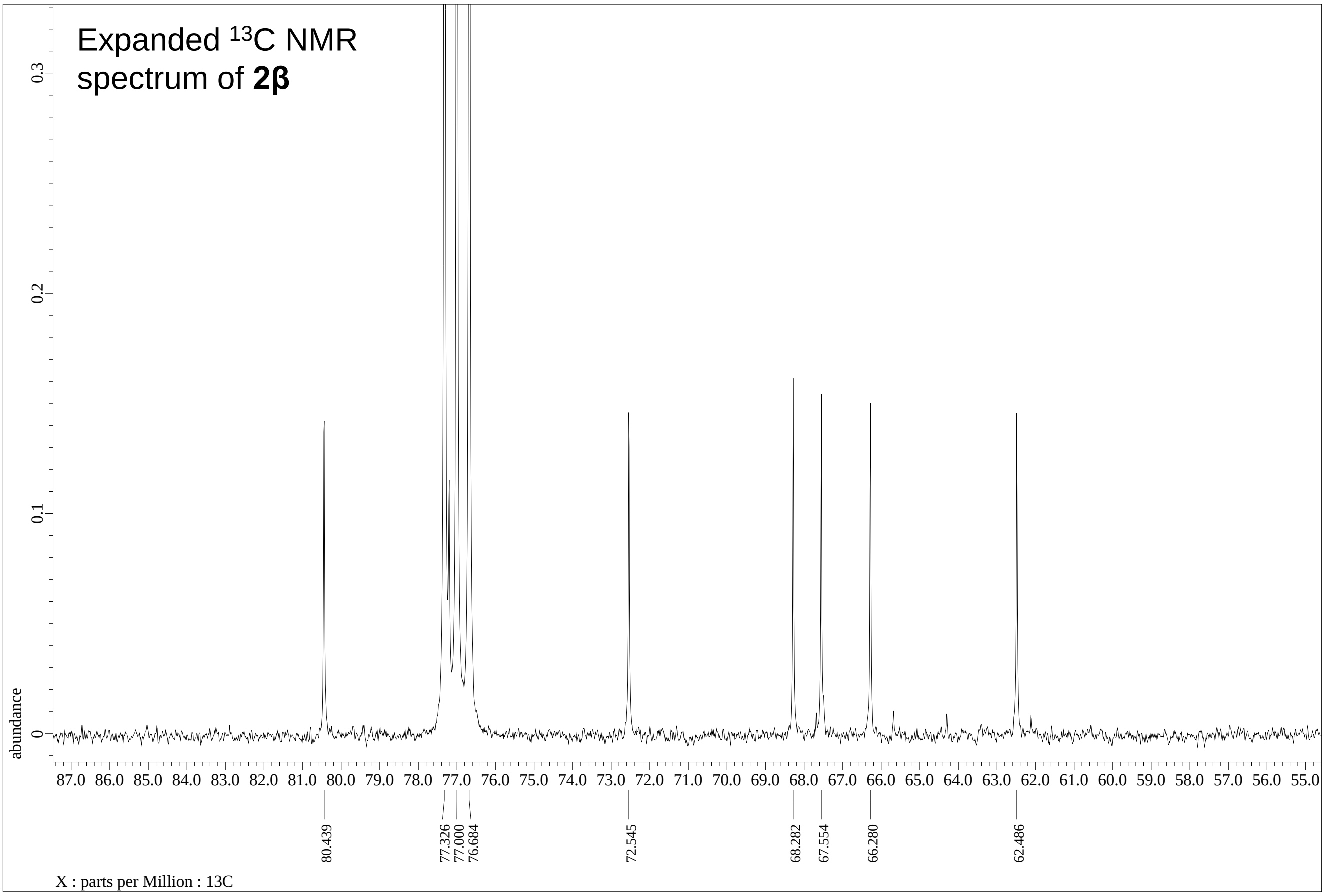

Expanded 13C NMR spectrum of 2β
abundance
80.439
77.326
77.000
76.684
72.545
68.282
67.554
66.280
62.486
X : parts per Million : 13C

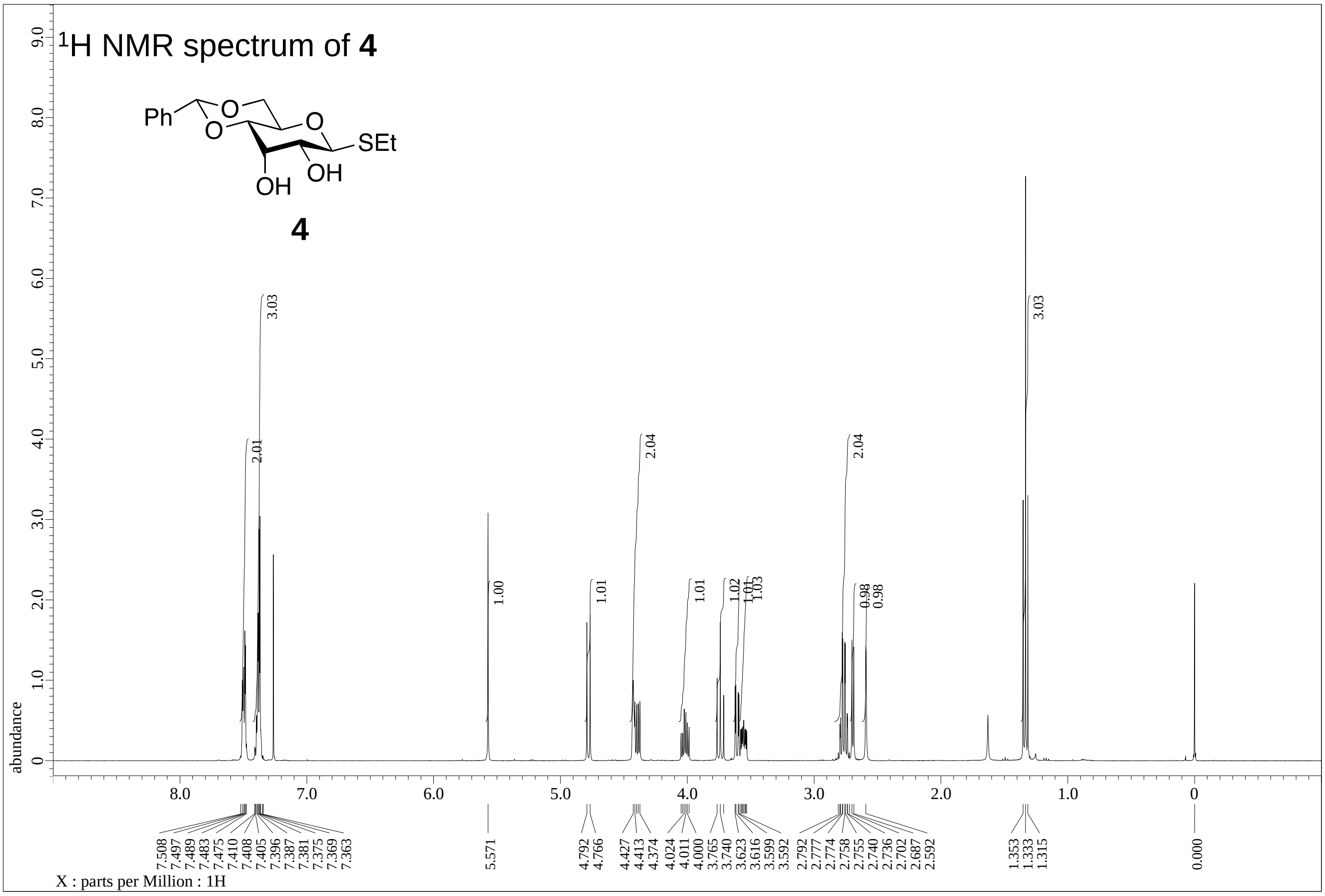

1H NMR spectrum of 4
Ph
O
O
O
SEt
OH
OH
4
abundance
X : parts per Million : 1H

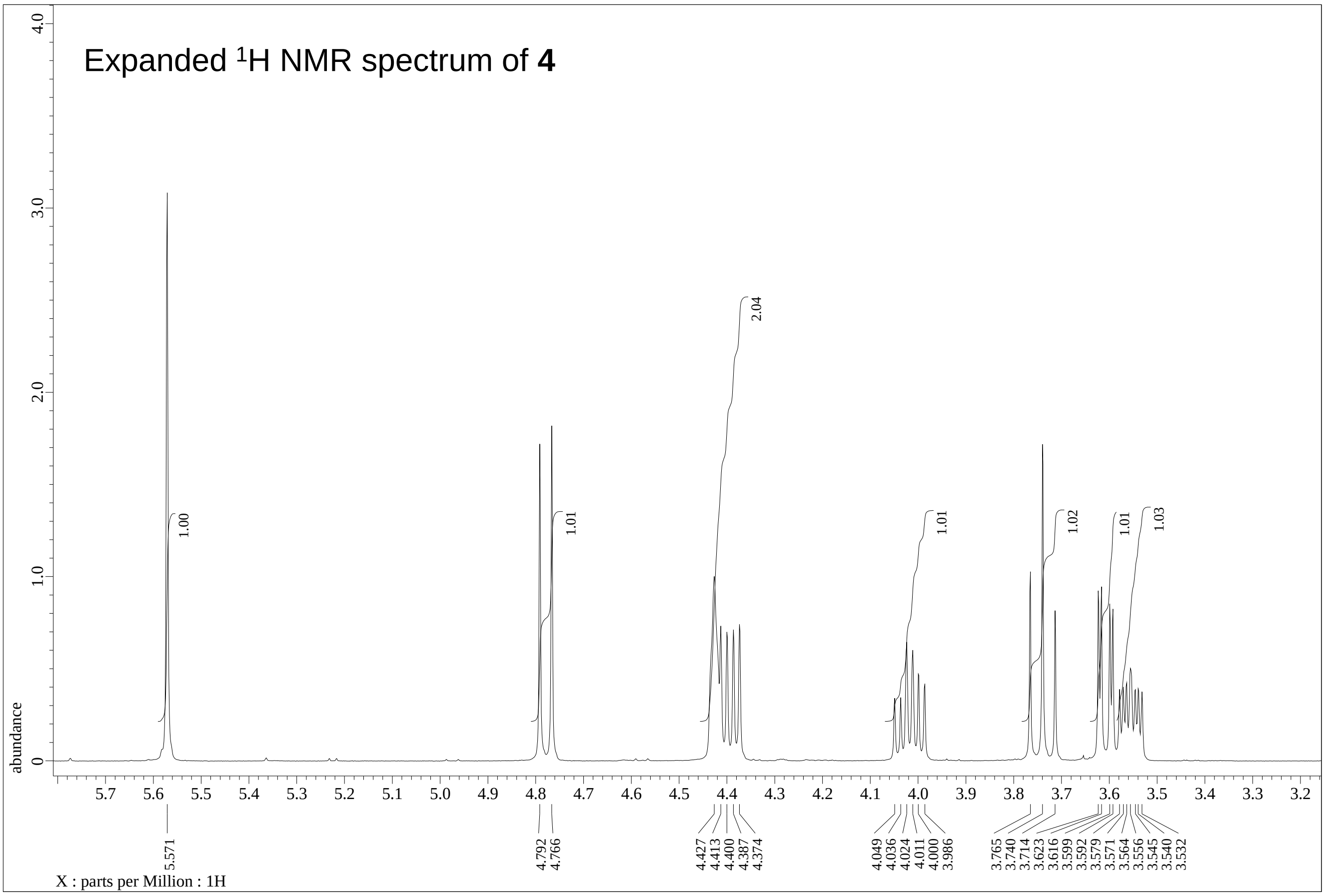

Expanded 1H NMR spectrum of 4
abundance
X : parts per Million : 1H

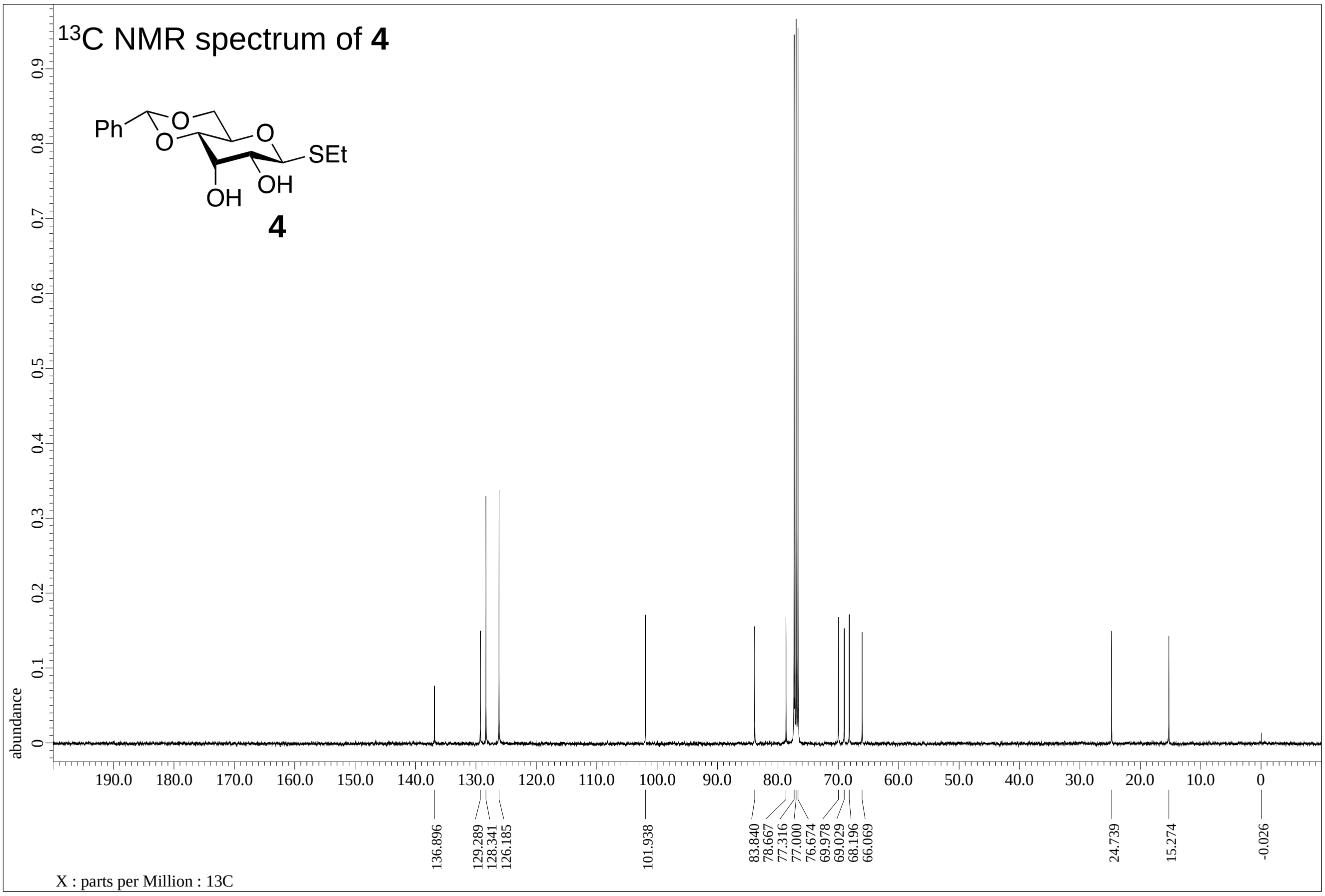
13C NMR spectrum of 4
Ph
O
O
O
SEt
OH
OH
4
abundance
0 0.1 0.2 0.3 0.4 0.5 0.6 0.7 0.8 0.9
190.0 180.0 170.0 160.0 150.0 140.0 130.0 120.0 110.0 100.0 90.0 80.0 70.0 60.0 50.0 40.0 30.0 20.0 10.0 0
136.896
129.289
128.341
126.185
101.938
83.840
78.667
77.316
77.000
76.674
69.978
69.029
68.196
66.069
24.739
15.274
-0.026
X : parts per Million : 13C

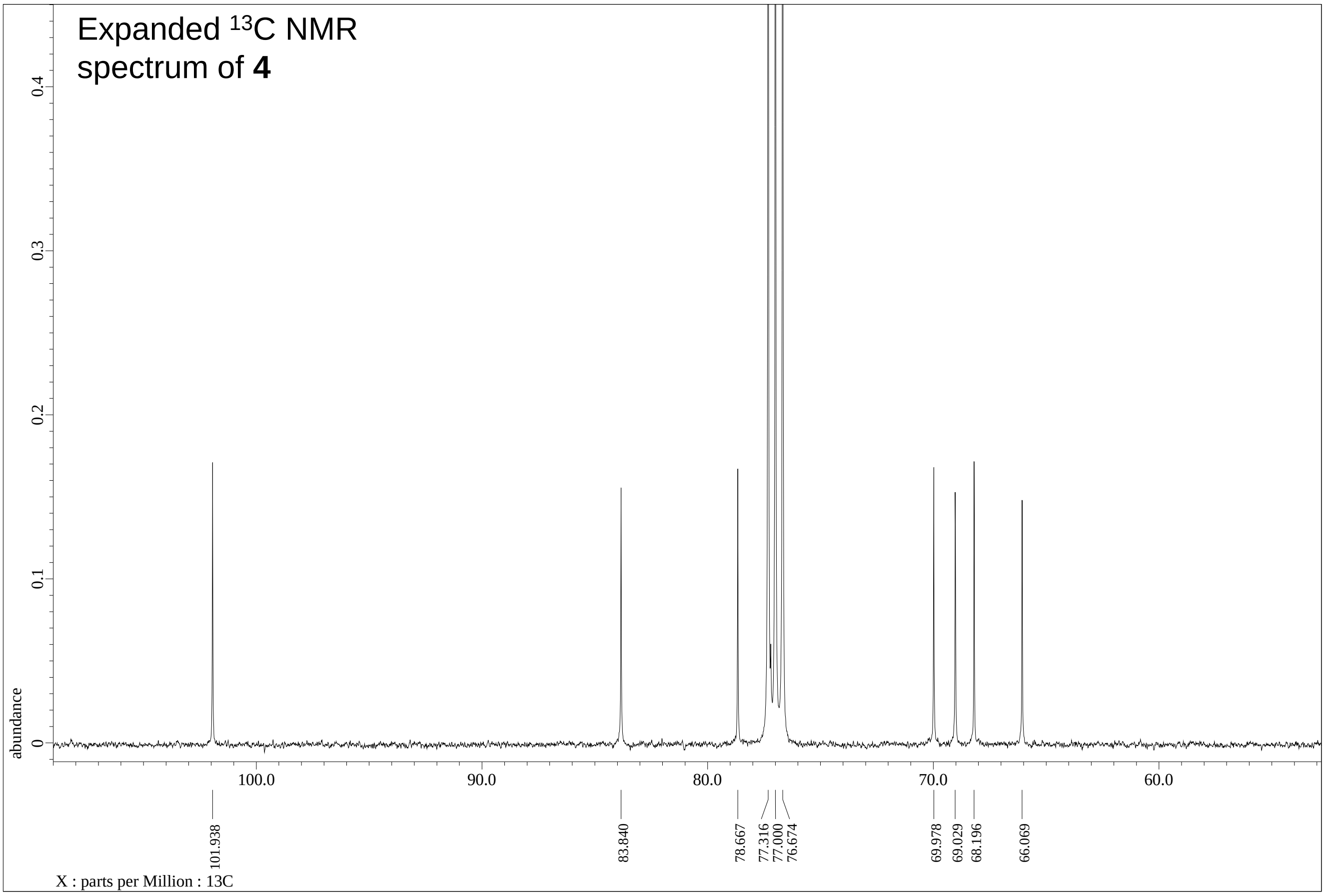
Expanded 13C NMR spectrum of 4
abundance
0 0.1 0.2 0.3 0.4
100.0 90.0 80.0 70.0 60.0
101.938
83.840
78.667
77.316
77.000
76.674
69.978
69.029
68.196
66.069
X : parts per Million : 13C

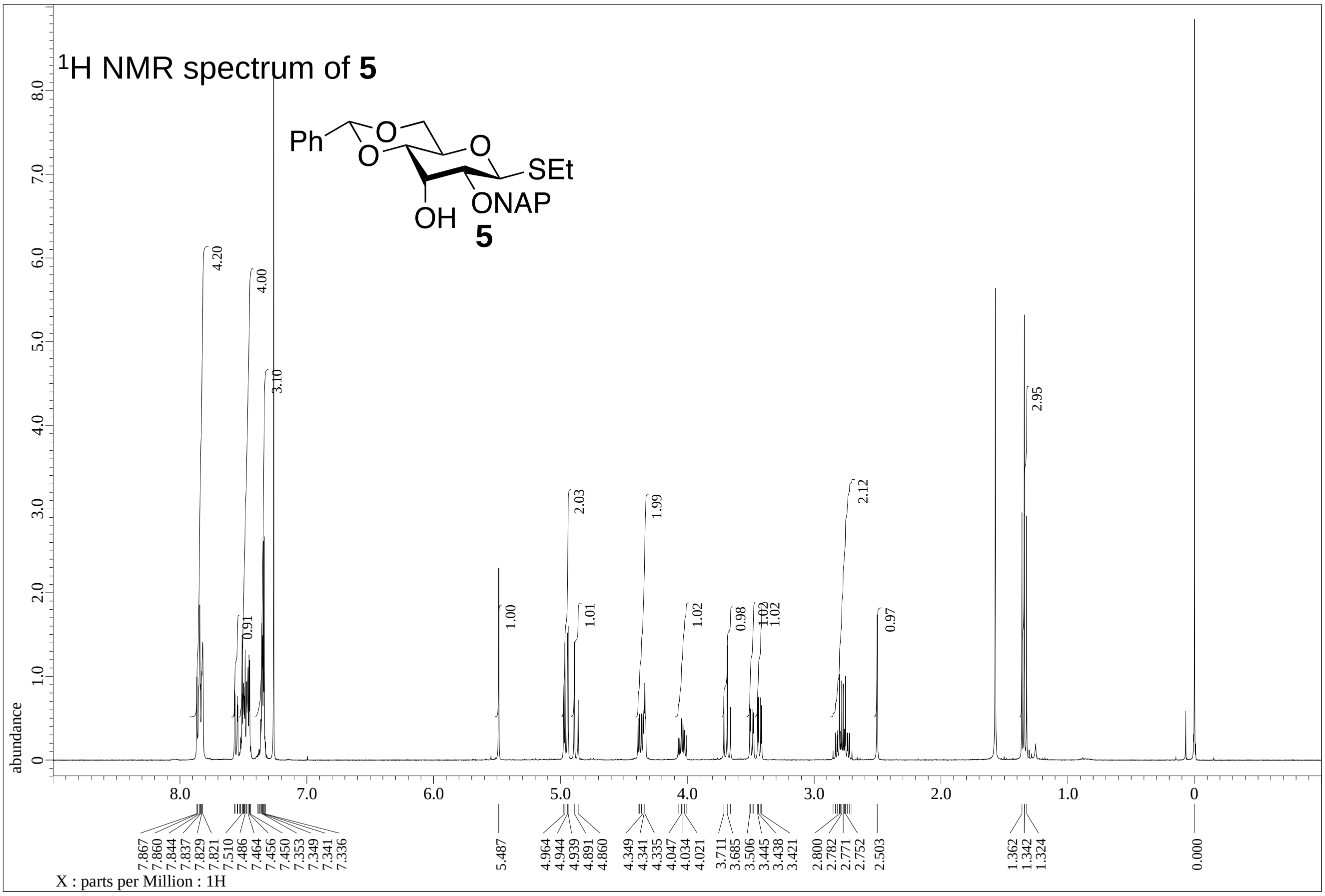
1H NMR spectrum of 5
Ph
O
O
O
SEt
OH
ONAP
5
abundance
X : parts per Million : 1H

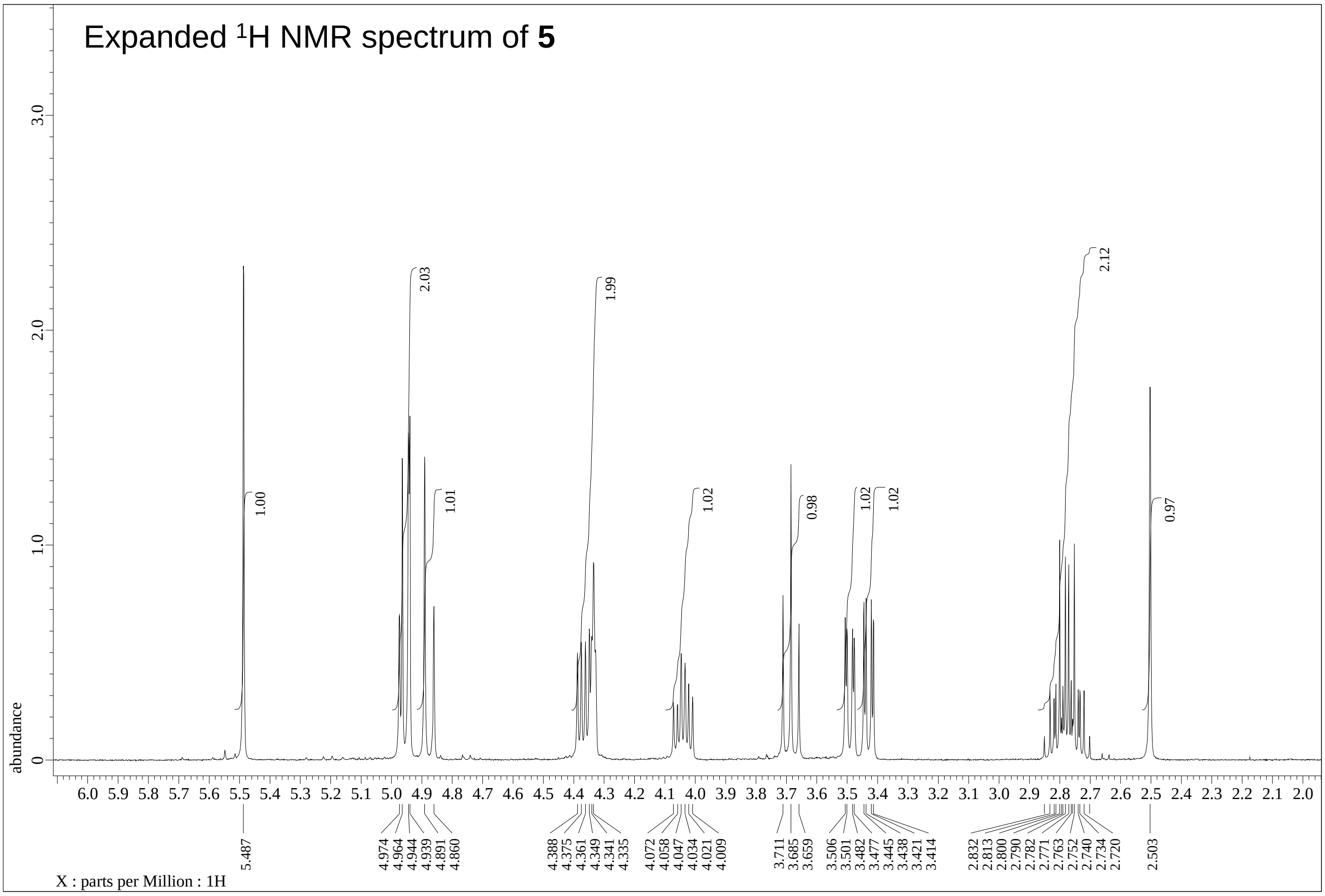
Expanded 1H NMR spectrum of 5
abundance
X : parts per Million : 1H

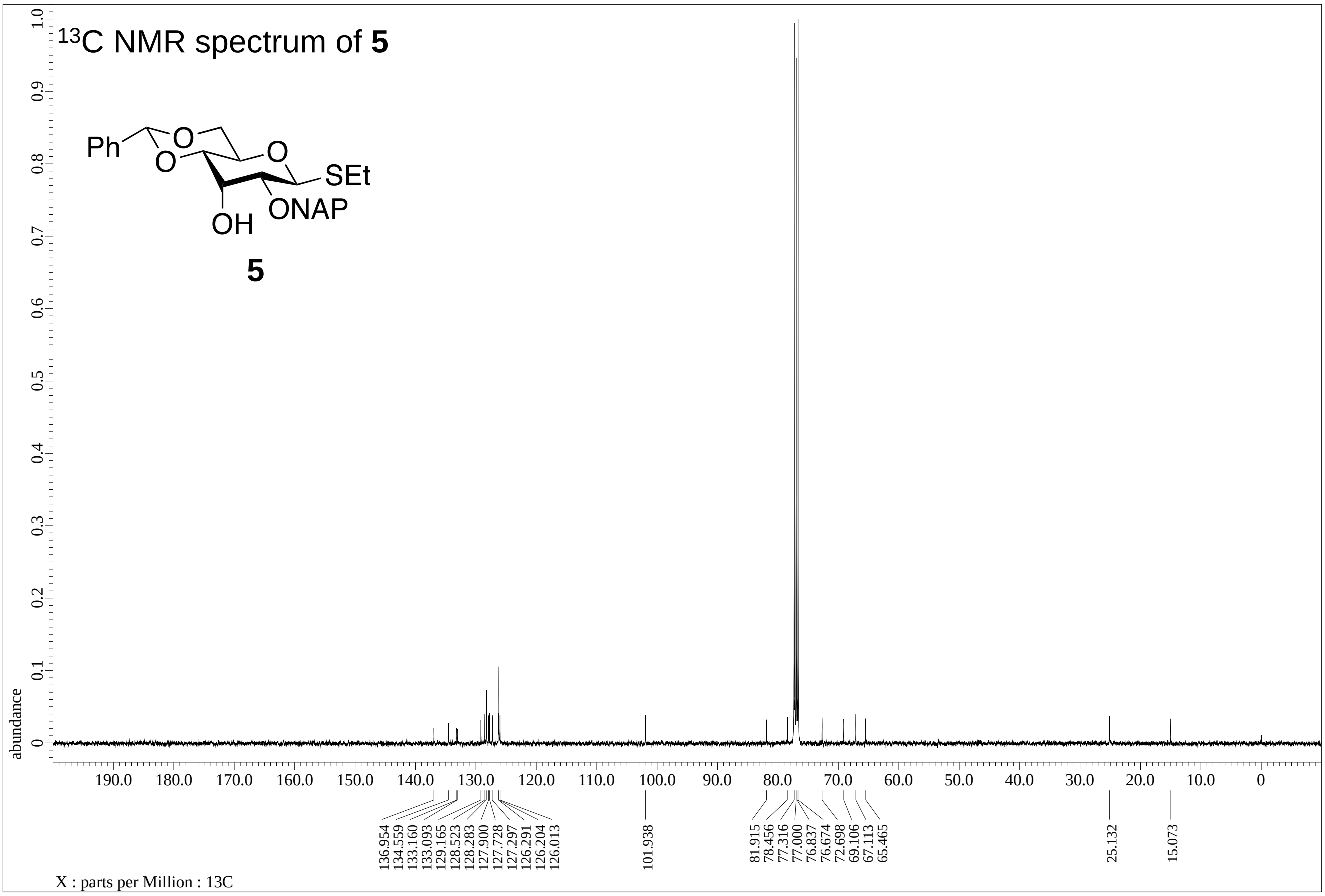
13C NMR spectrum of 5
Ph
O
O
O
SEt
OH
ONAP
5
abundance
136.954
134.559
133.160
133.093
129.165
128.523
128.283
127.900
127.728
127.297
126.291
126.204
126.013
101.938
81.915
78.456
77.316
77.000
76.837
76.674
72.698
69.106
67.113
65.465
25.132
15.073
X : parts per Million : 13C

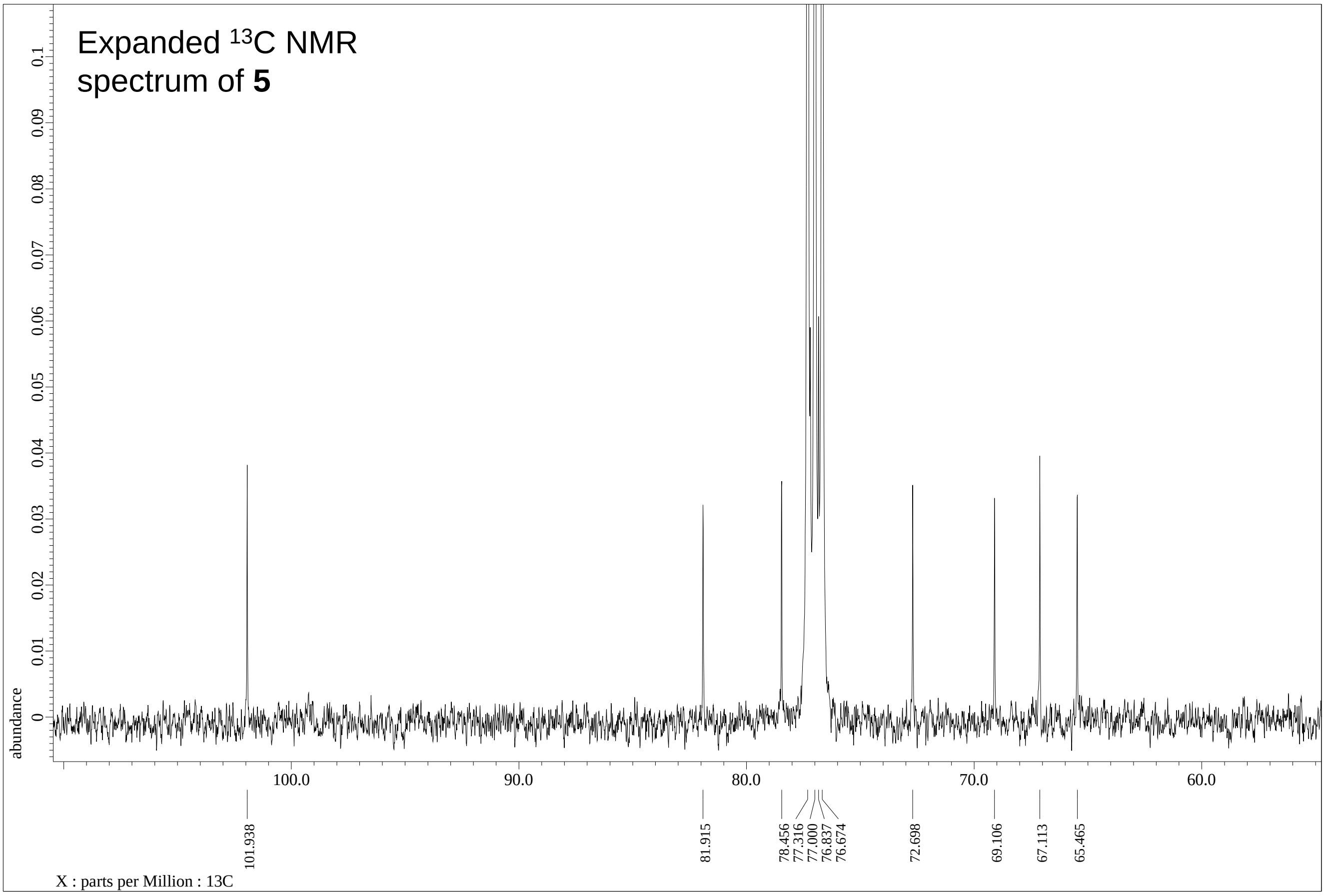
Expanded 13C NMR spectrum of 5
abundance
101.938
81.915
78.456
77.316
77.000
76.837
76.674
72.698
69.106
67.113
65.465
X : parts per Million : 13C

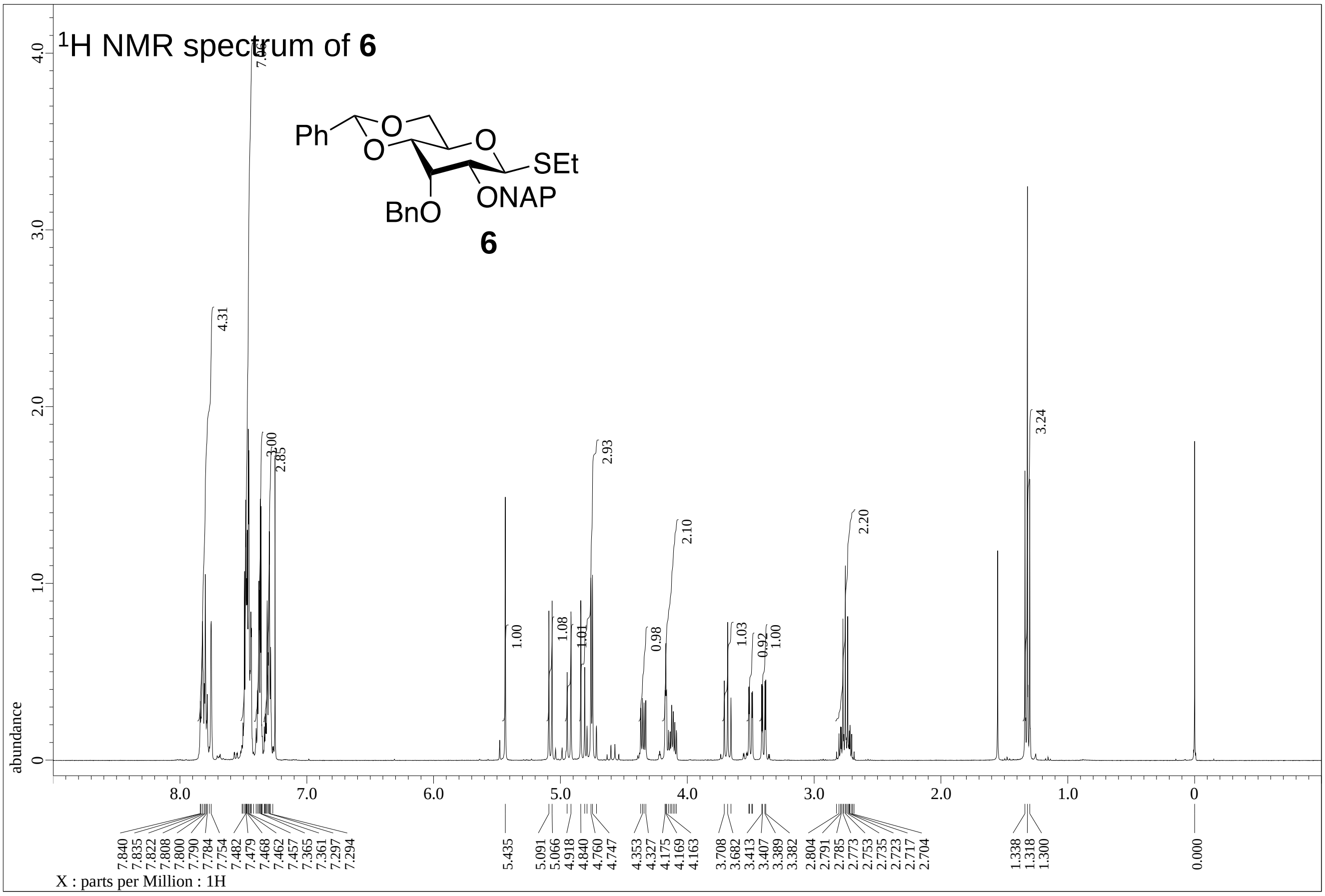

¹H NMR spectrum of 6
Ph
O
O
O
SEt
BnO
ONAP
6
abundance
X : parts per Million : 1H

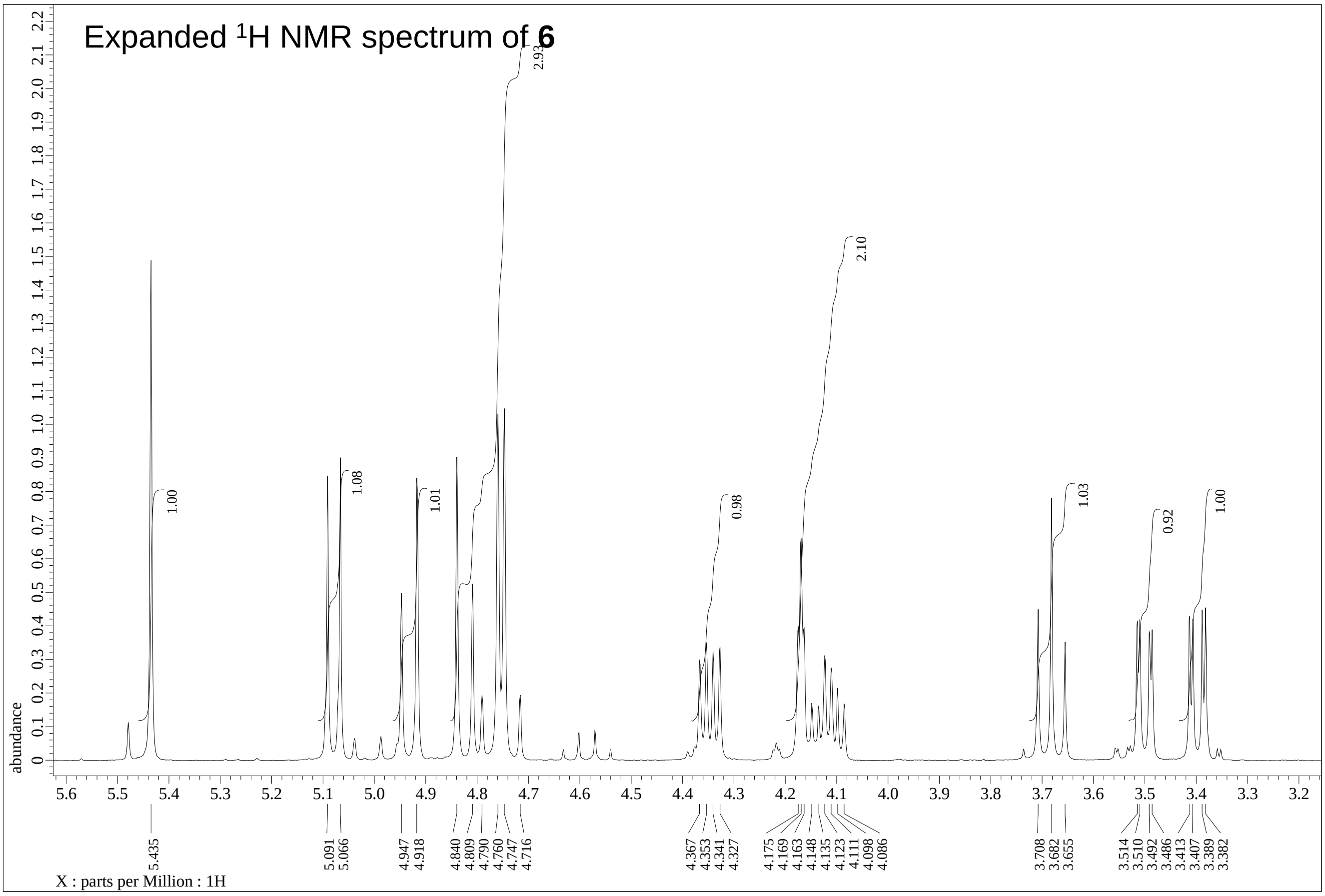

Expanded ¹H NMR spectrum of 6
abundance
X : parts per Million : 1H

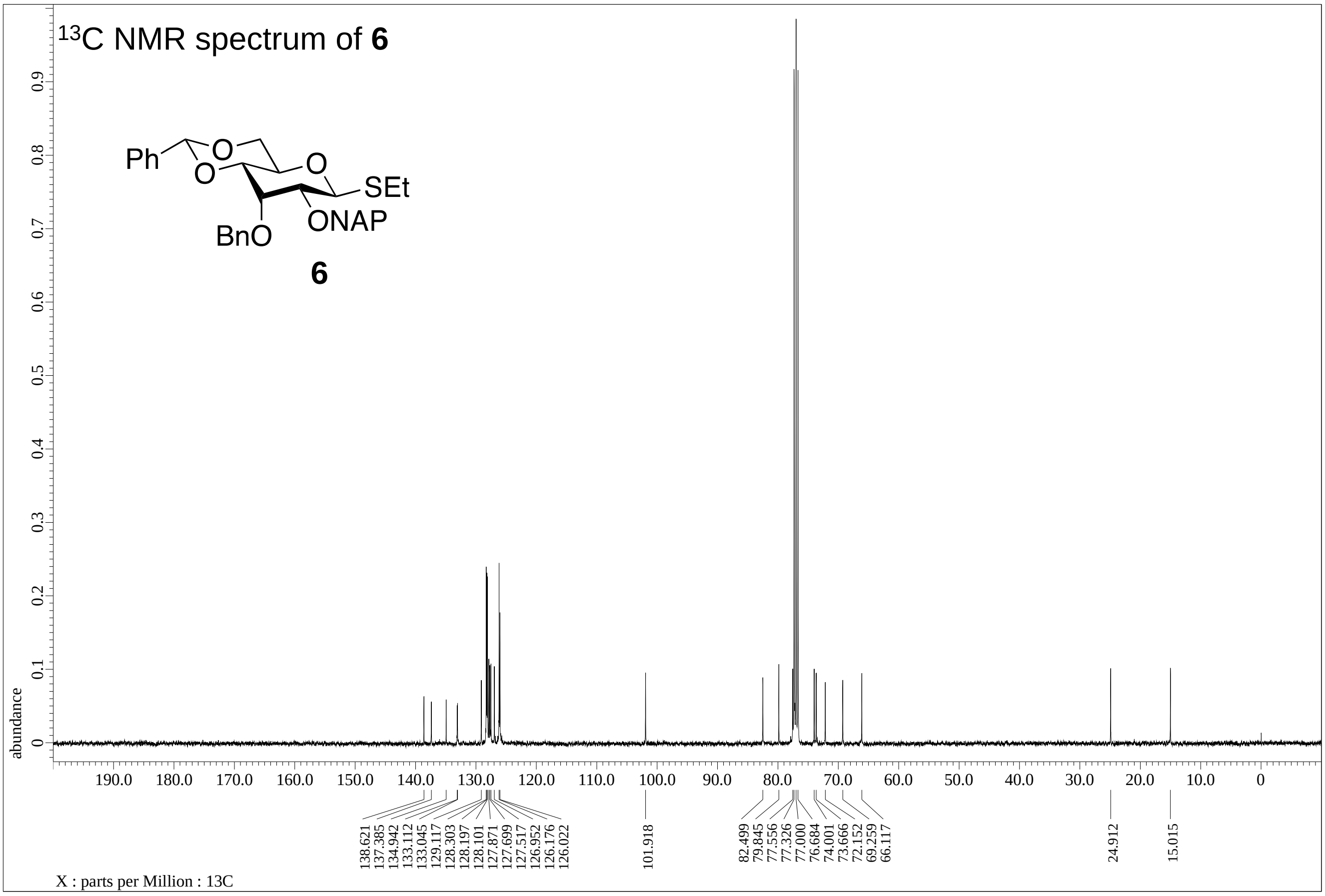
13C NMR spectrum of 6
Ph
O
O
O
SEt
BnO
ONAP
6
abundance
190.0 180.0 170.0 160.0 150.0 140.0 130.0 120.0 110.0 100.0 90.0 80.0 70.0 60.0 50.0 40.0 30.0 20.0 10.0 0
138.621
137.385
134.942
133.112
133.045
129.117
128.303
128.197
128.101
127.871
127.699
127.517
126.952
126.176
126.022
101.918
82.499
79.845
77.556
77.326
77.000
76.684
74.001
73.666
72.152
69.259
66.117
24.912
15.015
X : parts per Million : 13C

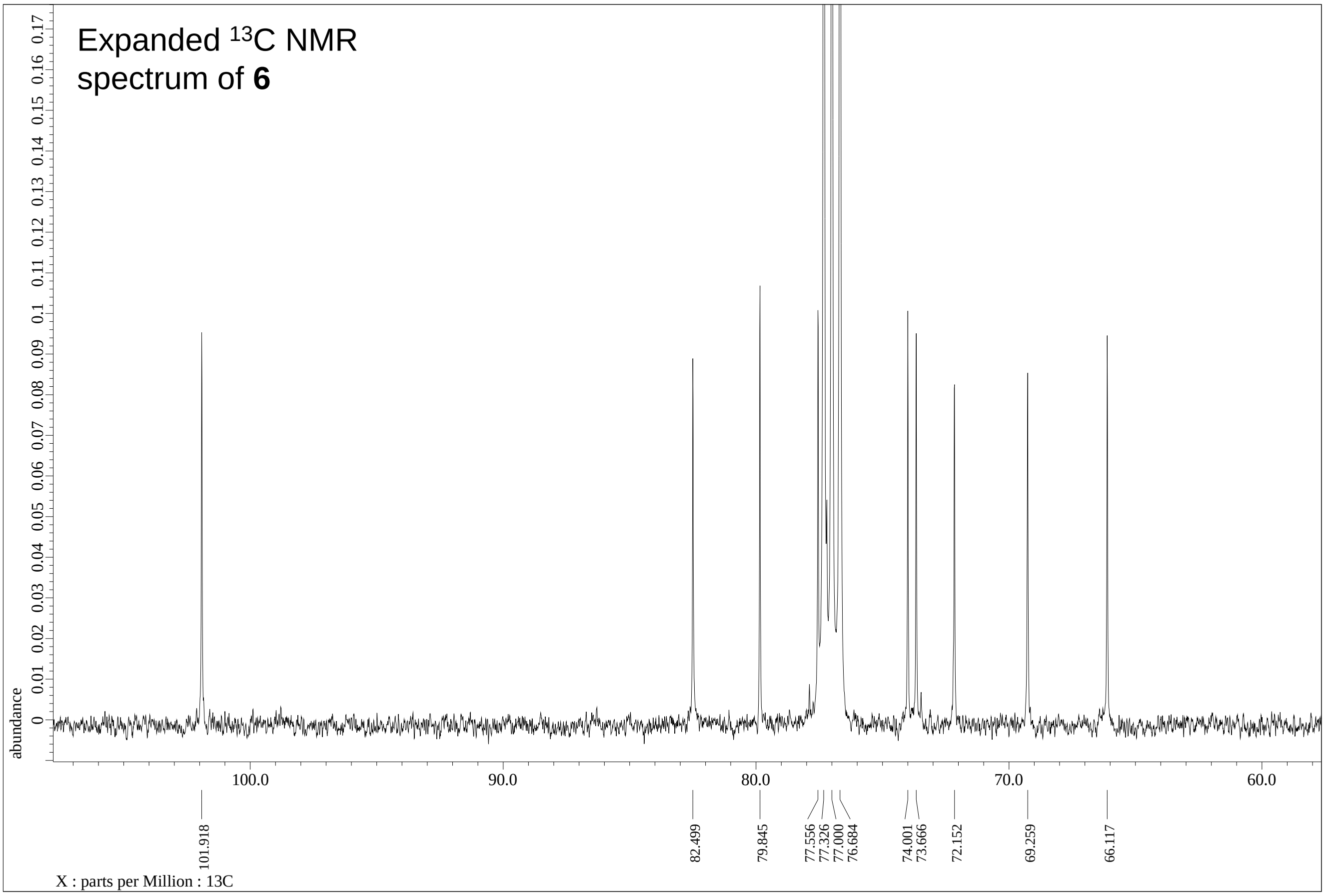
Expanded 13C NMR spectrum of 6
abundance
100.0 90.0 80.0 70.0 60.0
101.918
82.499
79.845
77.556
77.326
77.000
76.684
74.001
73.666
72.152
69.259
66.117
X : parts per Million : 13C

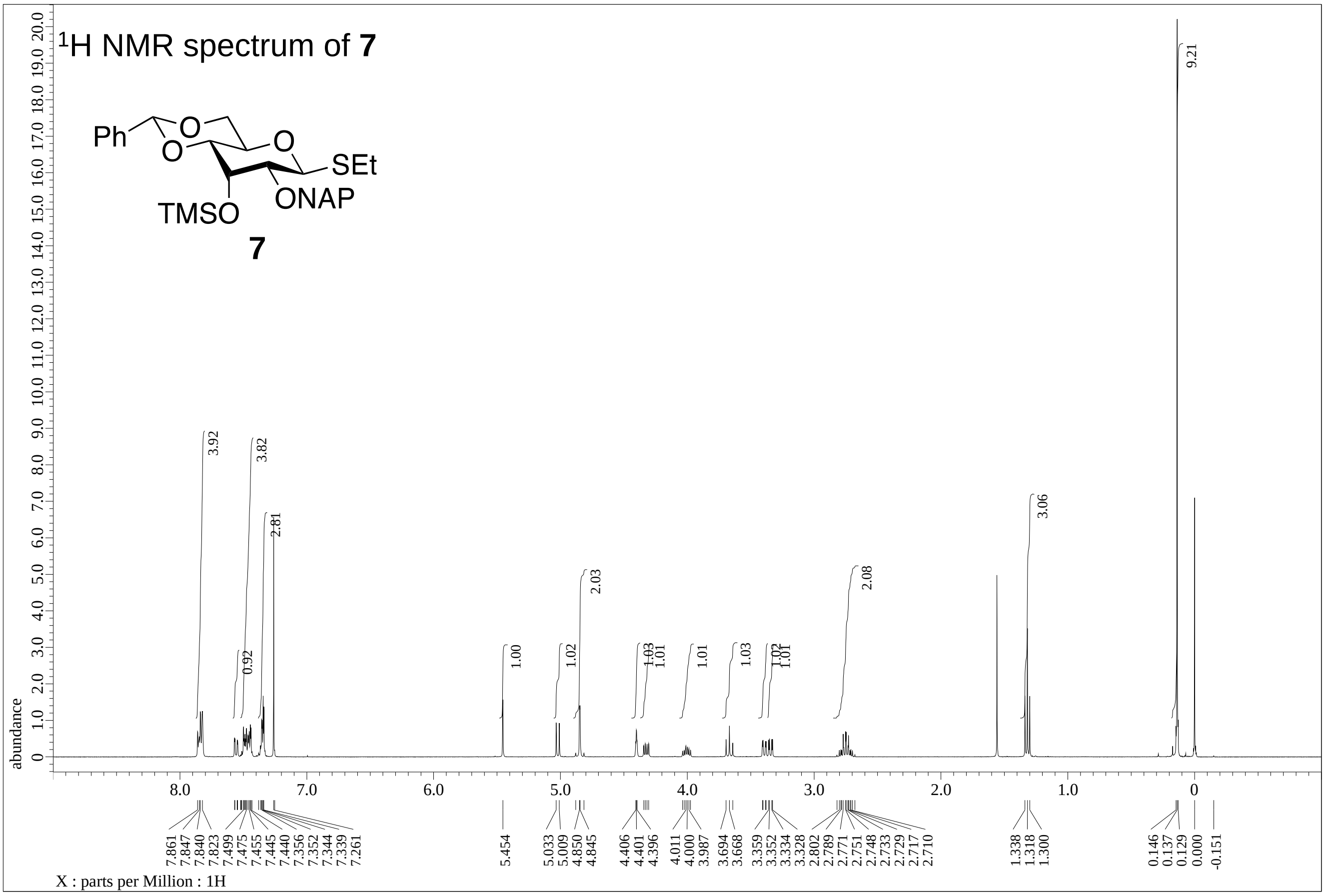

¹H NMR spectrum of 7
Ph
O
O
O
SEt
TMSO
ONAP
7
abundance
X : parts per Million : 1H

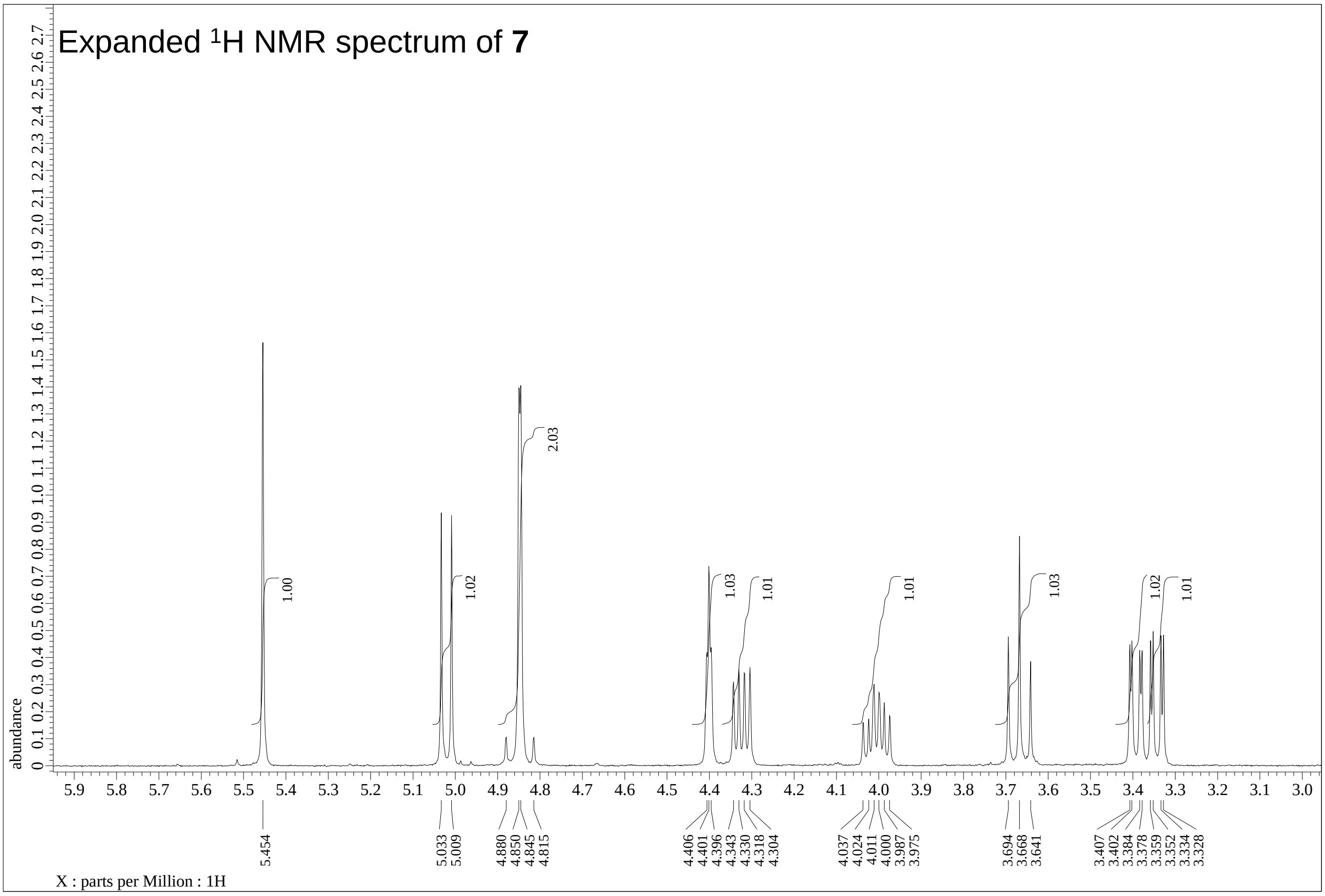

Expanded ¹H NMR spectrum of 7
abundance
X : parts per Million : 1H

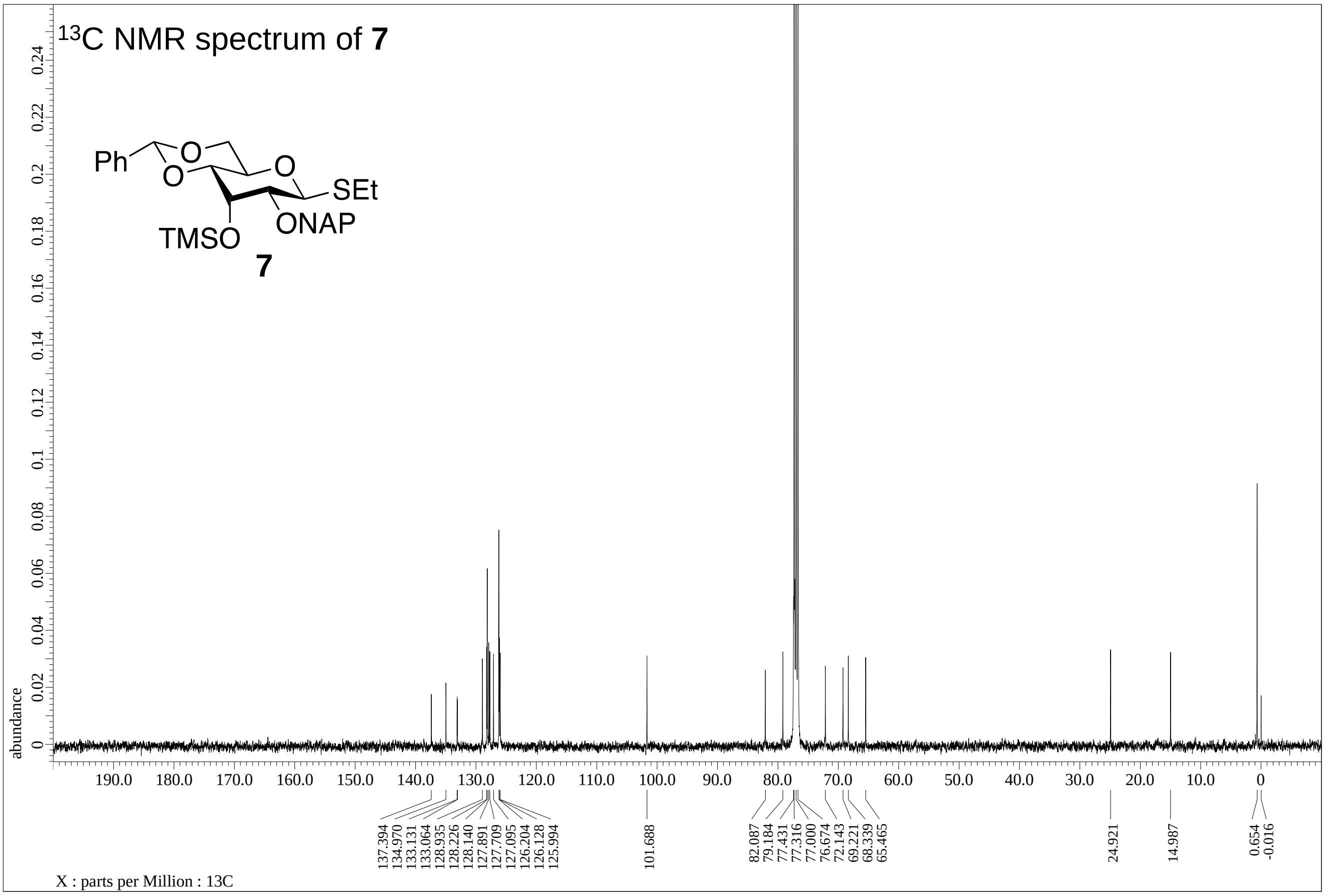

^{13}C NMR spectrum of **7**
Ph
O
O
O
SEt
ONAP
TMSO
7
abundance
X : parts per Million : 13C
137.394
134.970
133.131
133.064
128.935
128.226
128.140
127.891
127.709
127.095
126.204
126.128
125.994
101.688
82.087
79.184
77.431
77.316
77.000
76.674
72.143
69.221
68.339
65.465
24.921
14.987
0.654
-0.016

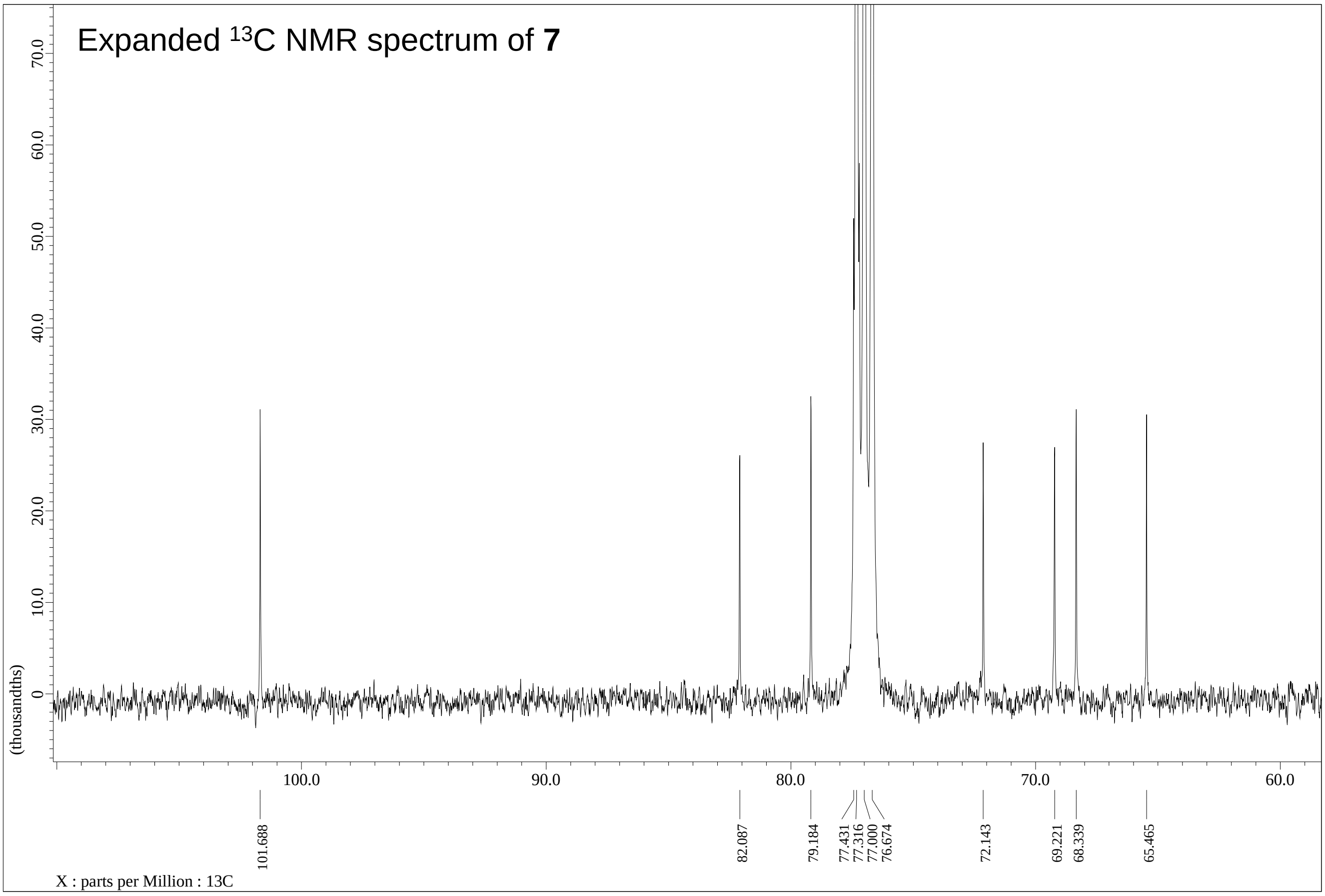

Expanded ^{13}C NMR spectrum of **7**
(thousandths)
X : parts per Million : 13C
101.688
82.087
79.184
77.431
77.316
77.000
76.674
72.143
69.221
68.339
65.465

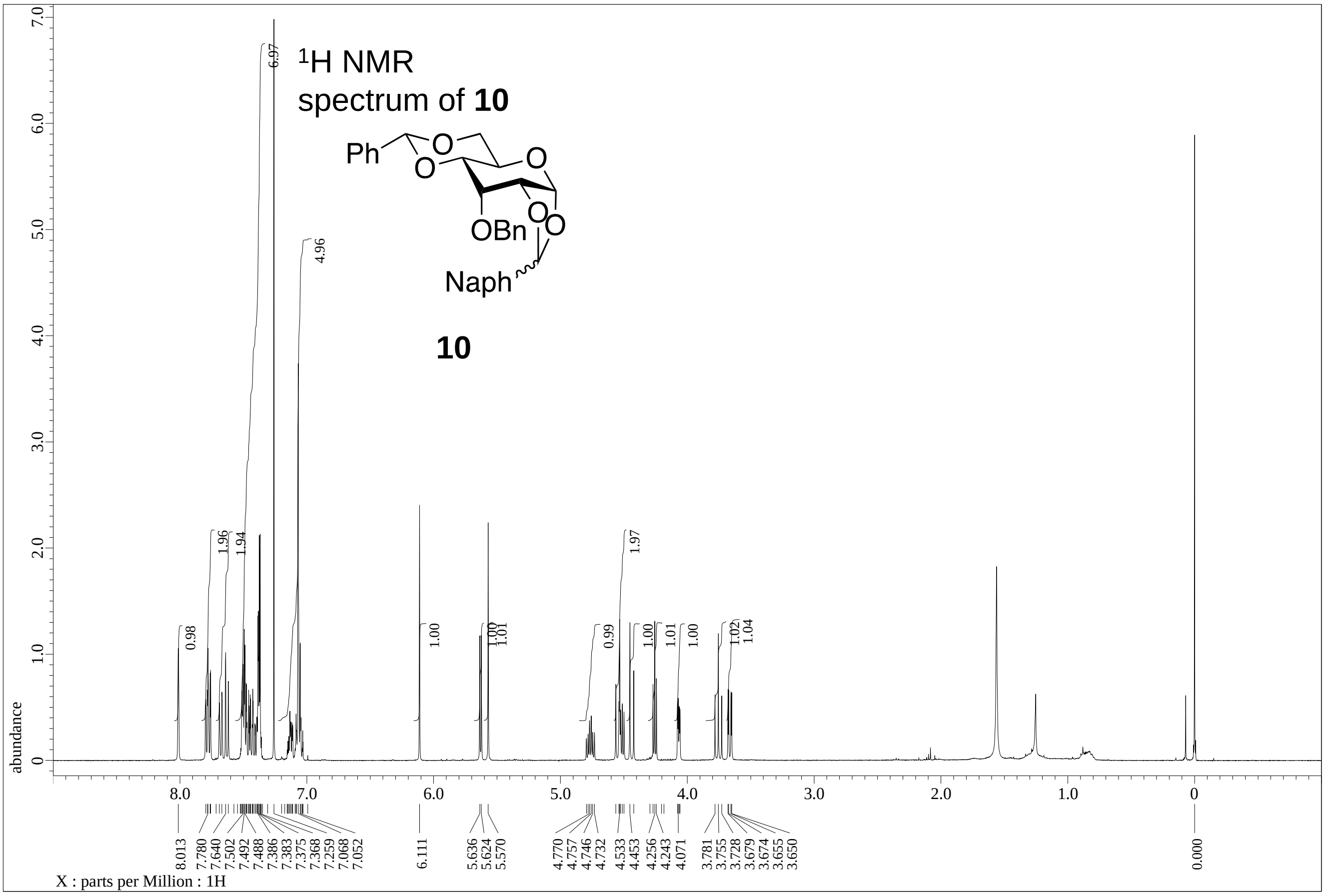
^{1}H NMR spectrum of **10**
Ph
O
O
O
OBn
O
O
Naph
10
abundance
X : parts per Million : 1H
8.013
7.780
7.640
7.502
7.492
7.488
7.386
7.383
7.375
7.368
7.259
7.068
7.052
6.111
5.636
5.624
5.570
4.770
4.757
4.746
4.732
4.533
4.453
4.256
4.243
4.071
3.781
3.755
3.728
3.679
3.674
3.655
3.650
0.000

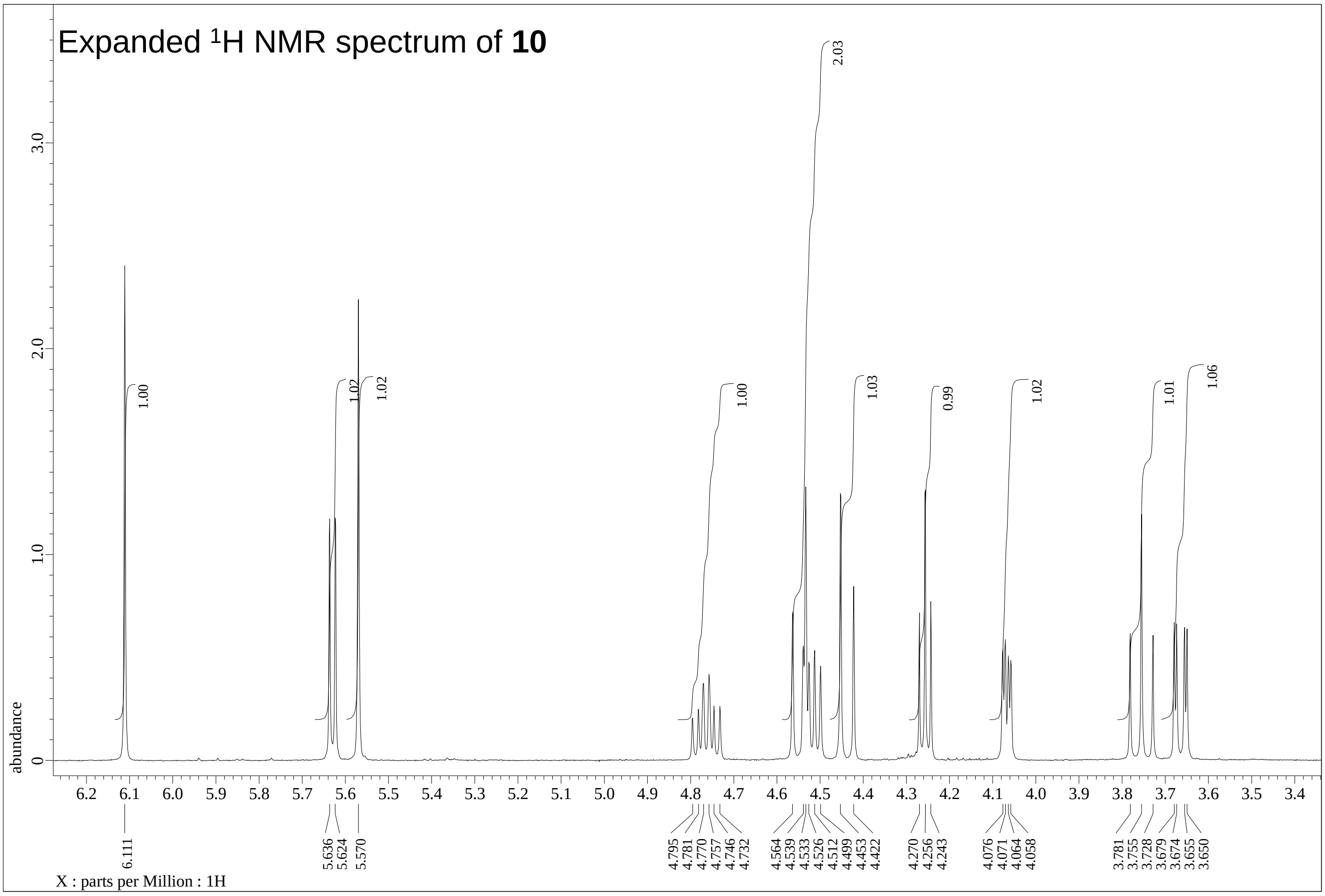
Expanded ^{1}H NMR spectrum of **10**
abundance
X : parts per Million : 1H
6.111
5.636
5.624
5.570
4.795
4.781
4.770
4.757
4.746
4.732
4.564
4.539
4.533
4.526
4.512
4.499
4.453
4.422
4.270
4.256
4.243
4.076
4.071
4.064
4.058
3.781
3.755
3.728
3.679
3.674
3.655
3.650

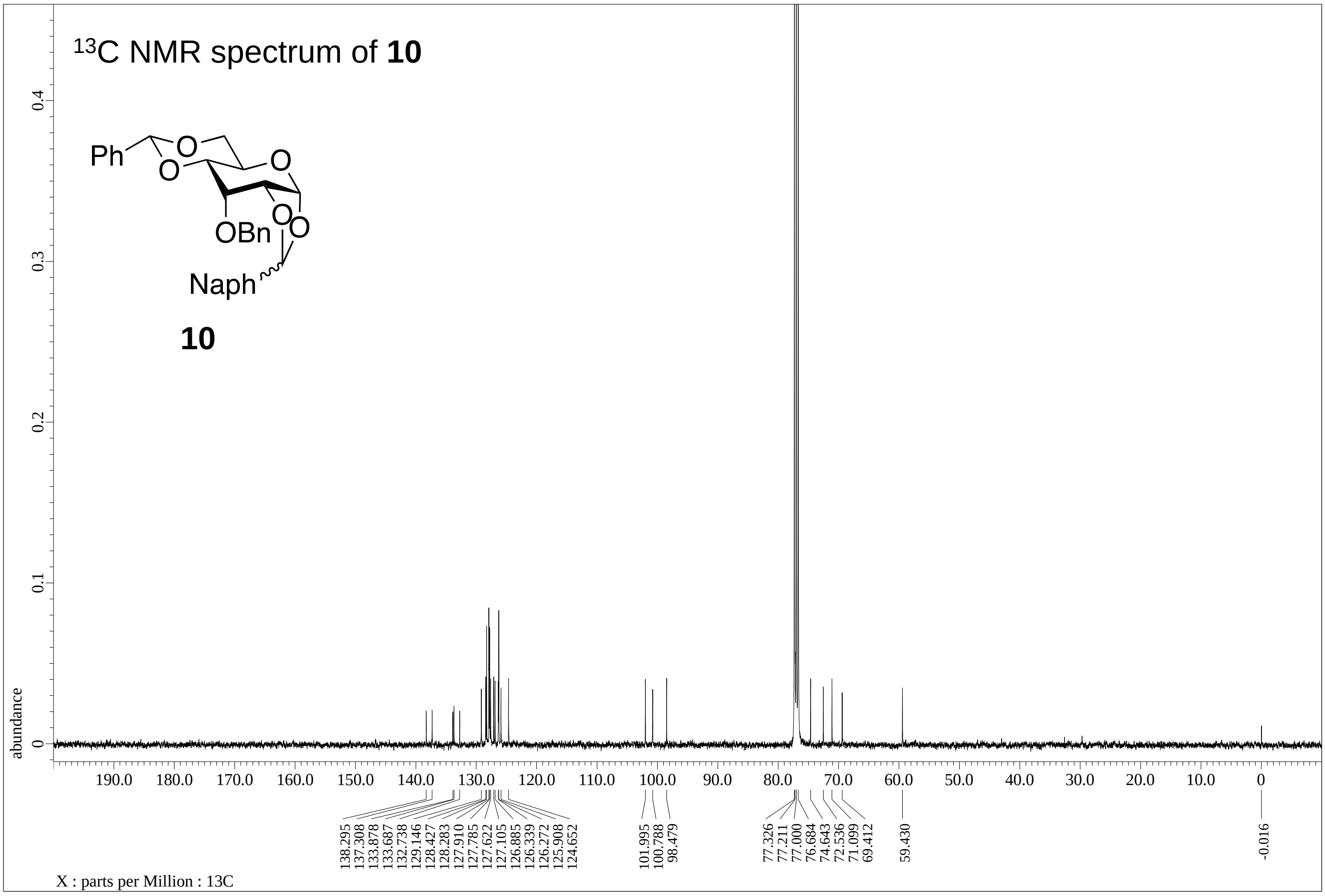

13C NMR spectrum of 10
Ph
O
O
O
O
O
OBn
Naph
10
abundance
X : parts per Million : 13C
138.295
137.308
133.878
133.687
132.738
129.146
128.427
128.283
127.910
127.785
127.622
127.105
126.885
126.339
126.272
125.908
124.652
101.995
100.788
98.479
77.326
77.211
77.000
76.684
74.643
72.536
71.099
69.412
59.430
-0.016

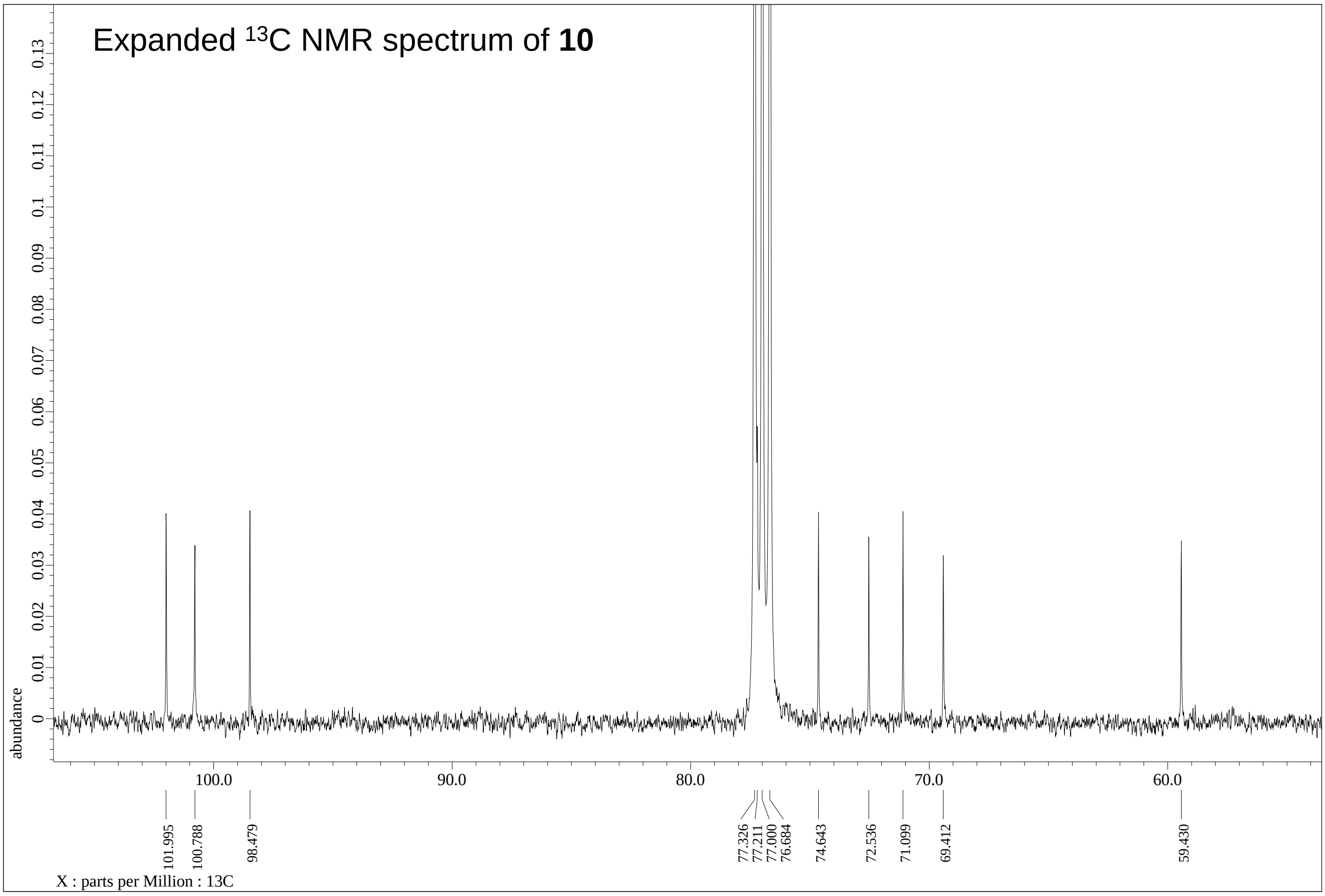

Expanded 13C NMR spectrum of 10
abundance
X : parts per Million : 13C
101.995
100.788
98.479
77.326
77.211
77.000
76.684
74.643
72.536
71.099
69.412
59.430

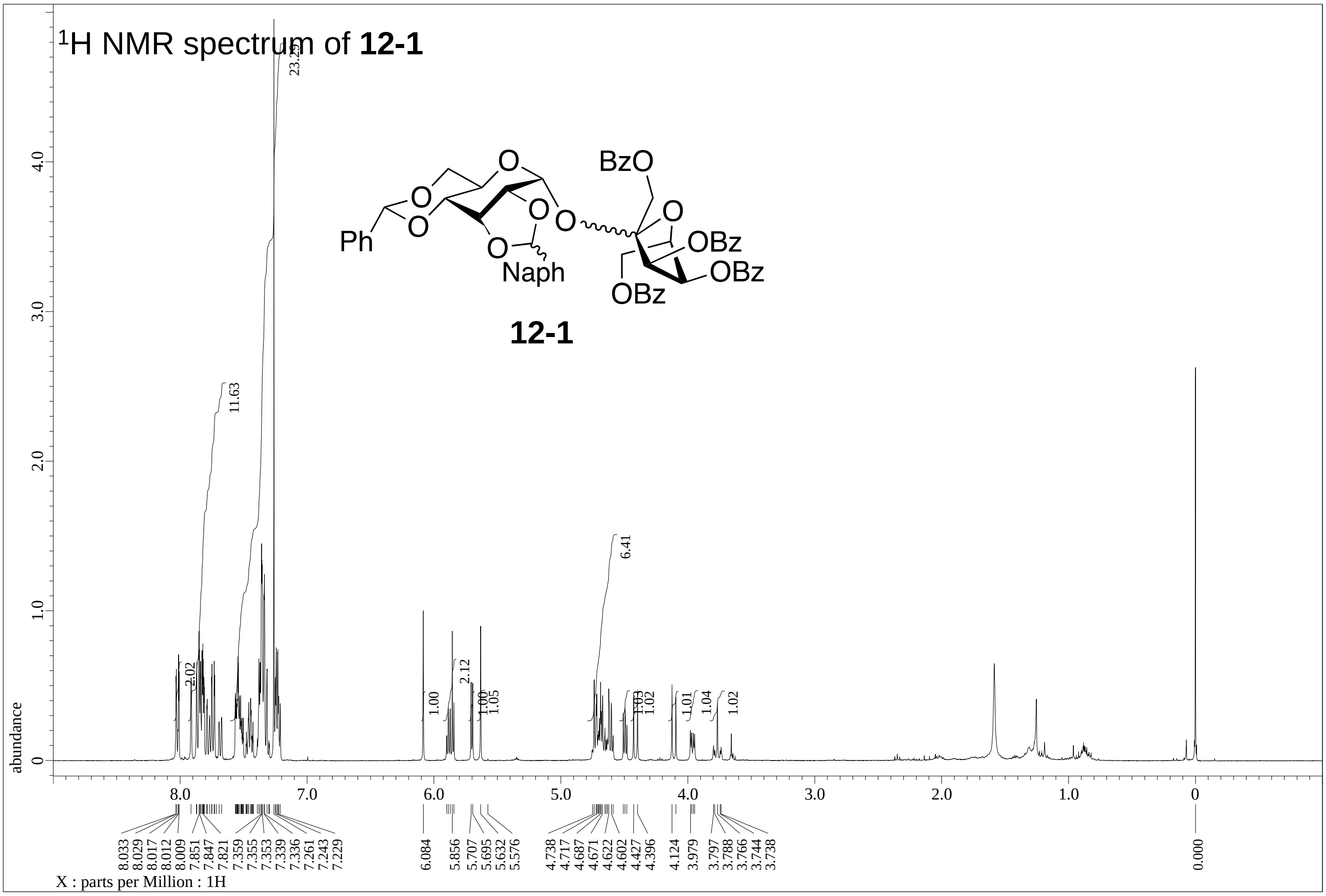
1H NMR spectrum of 12-1
BzO
O
O
O
O
O
O
Ph
Naph
OBz
OBz
OBz
12-1
abundance
X : parts per Million : 1H
8.033 8.029 8.017 8.012 8.009 7.851 7.847 7.821 7.359 7.355 7.353 7.339 7.336 7.261 7.243 7.229
6.084 5.856 5.707 5.695 5.632 5.576
4.738 4.717 4.687 4.671 4.622 4.602 4.427 4.396
4.124 3.979 3.797 3.788 3.766 3.744 3.738
0.000
2.02 11.63 23.25 1.00 2.12 1.00 1.05 6.41 1.03 1.02 1.01 1.04 1.02

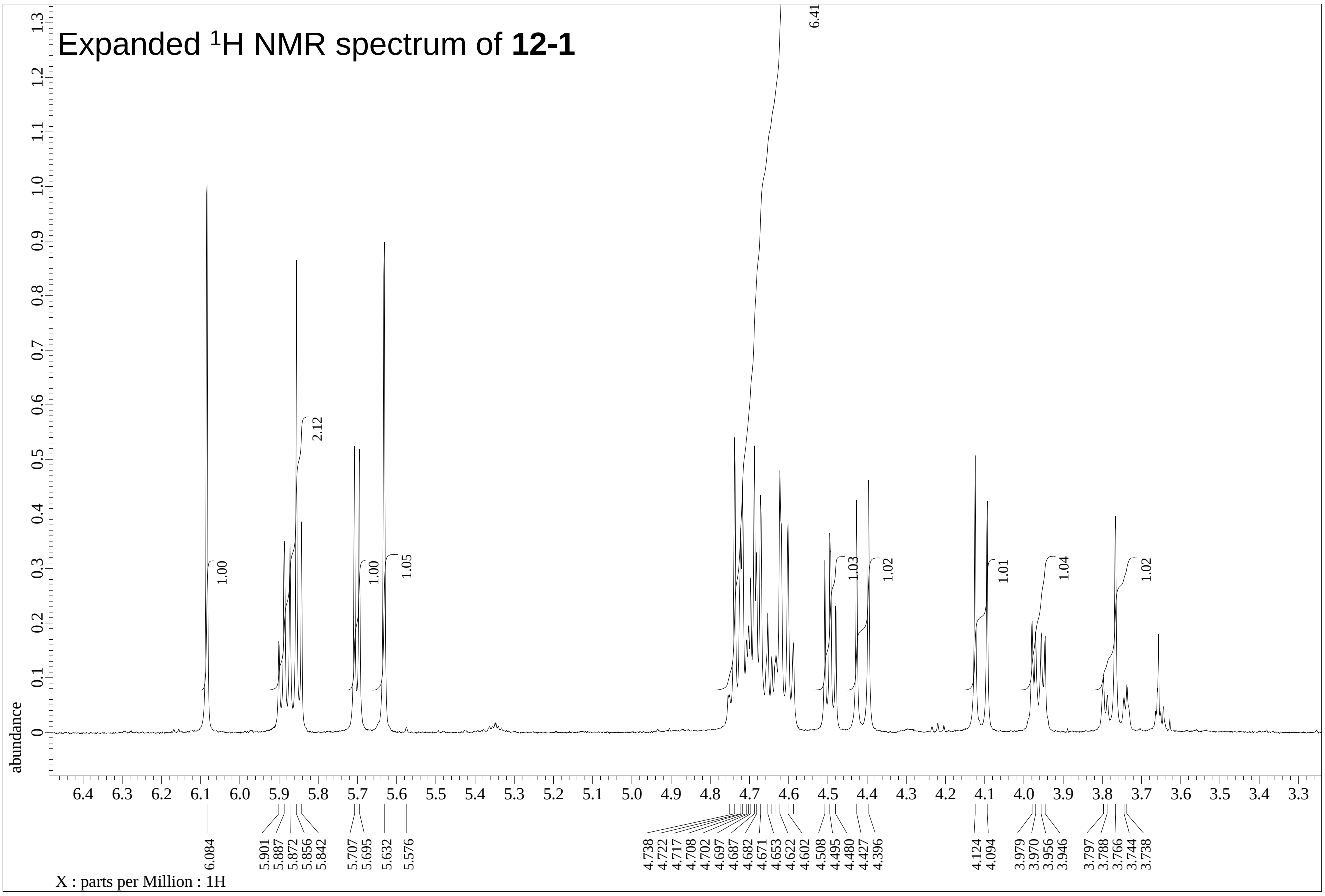
Expanded 1H NMR spectrum of 12-1
abundance
X : parts per Million : 1H
6.084
5.901 5.887 5.872 5.856 5.842
5.707 5.695 5.632 5.576
4.738 4.722 4.717 4.708 4.702 4.697 4.687 4.682 4.671 4.653 4.622 4.602
4.508 4.495 4.480 4.427 4.396
4.124 4.094
3.979 3.970 3.956 3.946
3.797 3.788 3.766 3.744 3.738
1.00 2.12 1.00 1.05 6.41 1.03 1.02 1.01 1.04 1.02

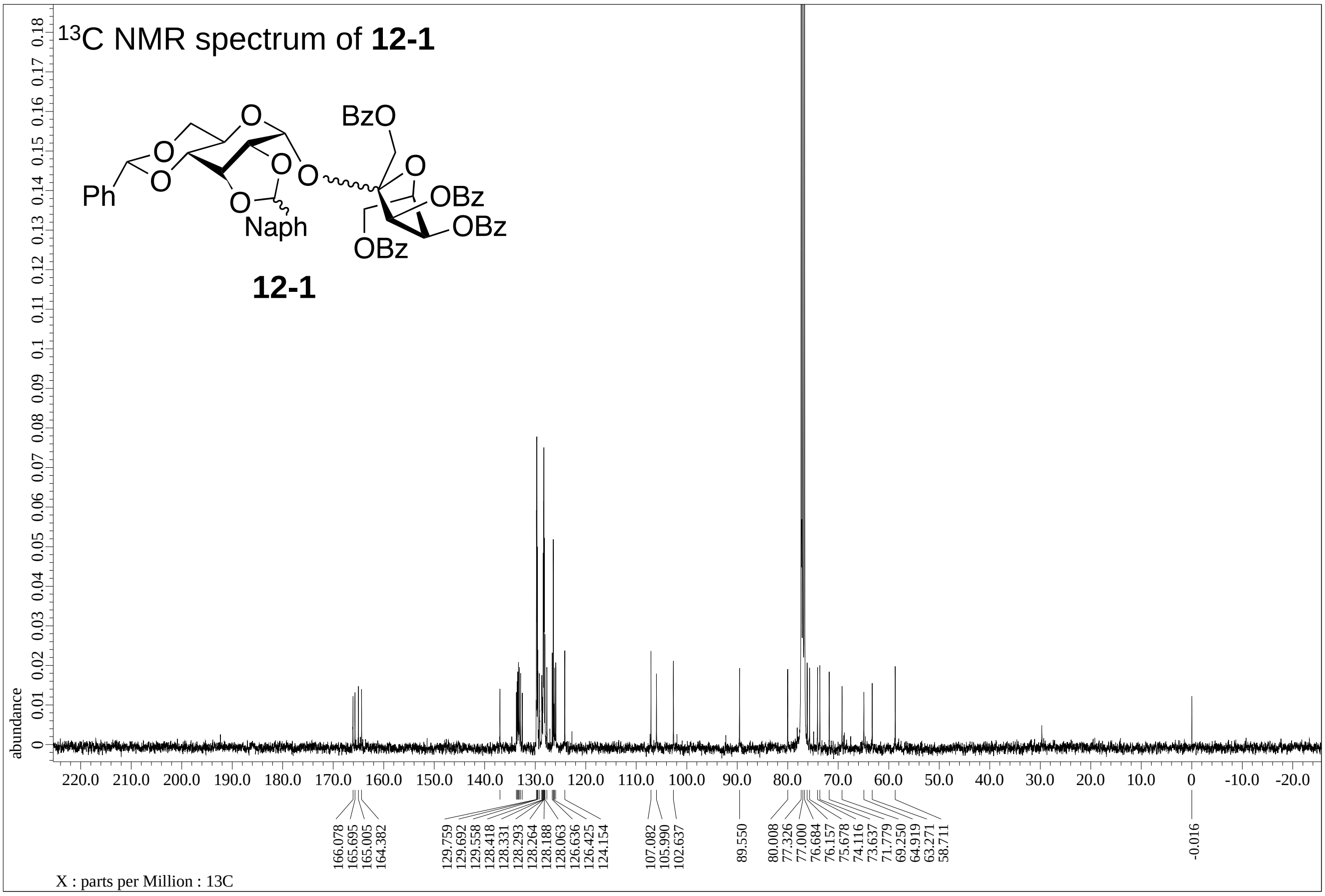

13C NMR spectrum of 12-1
BzO
O
O
O
O
O
O
OBz
OBz
OBz
Ph
Naph
12-1
abundance
166.078
165.695
165.005
164.382
129.759
129.692
129.558
128.418
128.331
128.293
128.264
128.188
128.063
126.636
126.425
124.154
107.082
105.990
102.637
89.550
80.008
77.326
77.000
76.684
76.157
75.678
74.116
73.637
71.779
69.250
64.919
63.271
58.711
-0.016
X : parts per Million : 13C

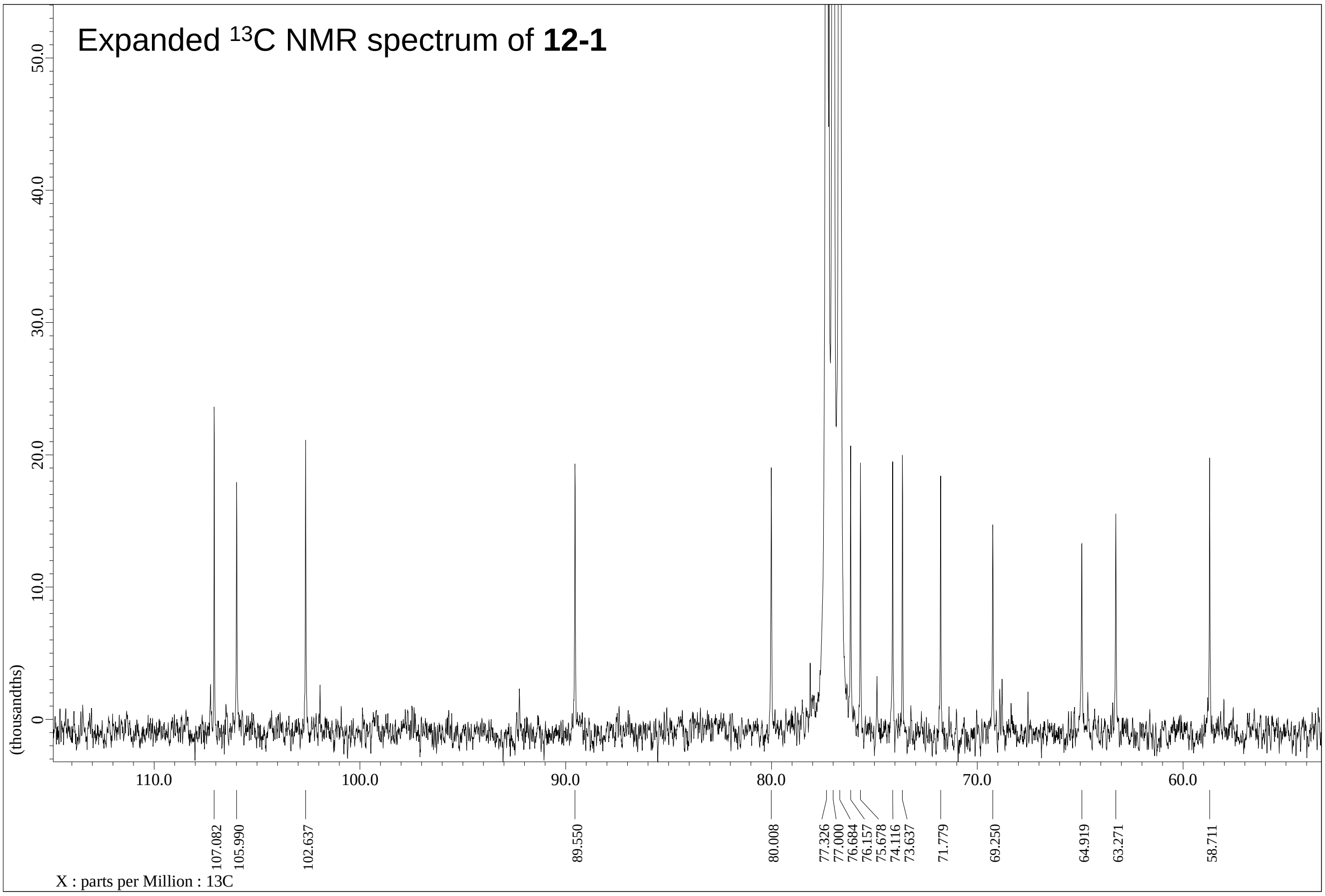

Expanded 13C NMR spectrum of 12-1
(thousandths)
107.082
105.990
102.637
89.550
80.008
77.326
77.000
76.684
76.157
75.678
74.116
73.637
71.779
69.250
64.919
63.271
58.711
X : parts per Million : 13C

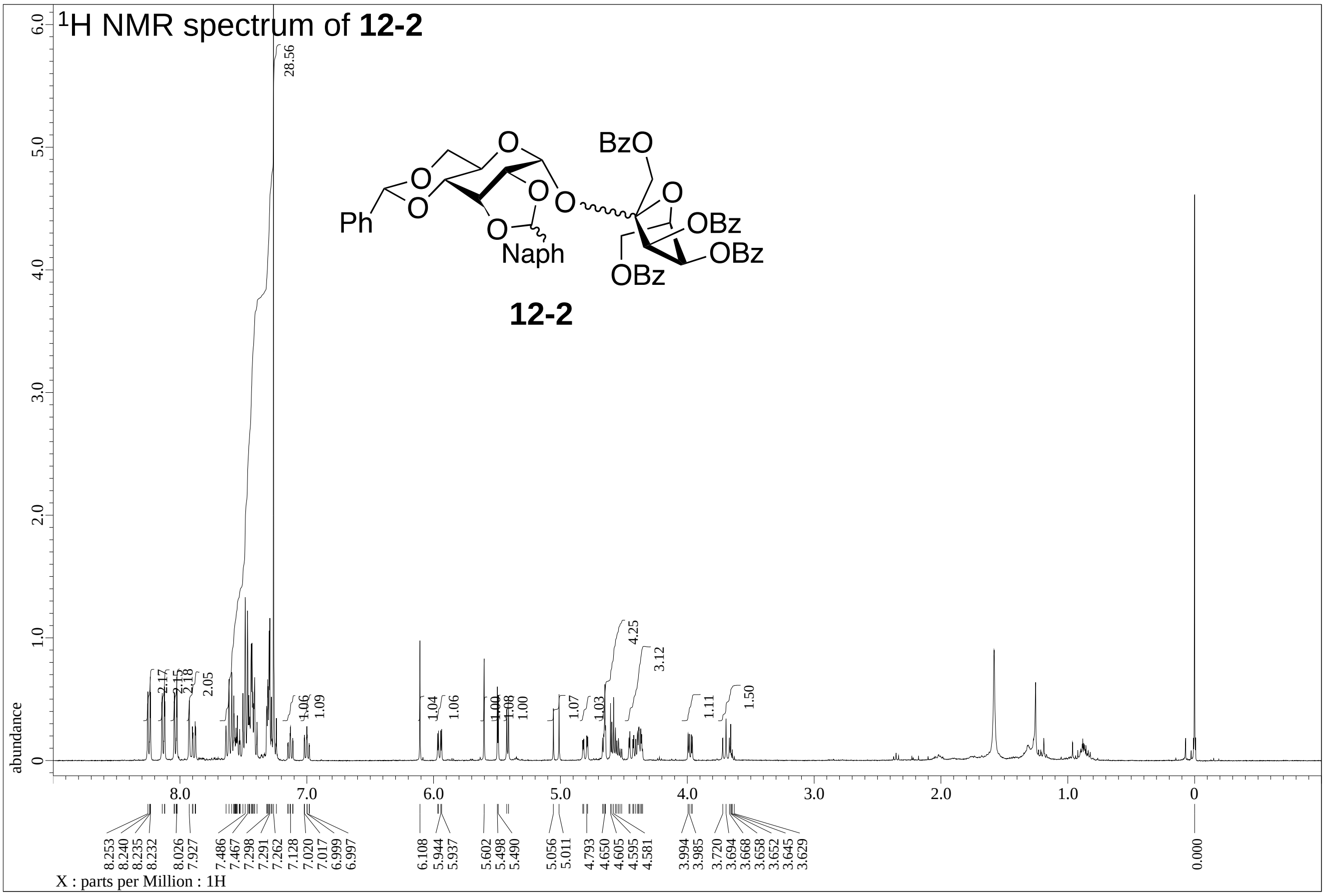

¹H NMR spectrum of 12-2
BzO
O
OBz
OBz
OBz
Ph
Naph
12-2
abundance
X : parts per Million : 1H

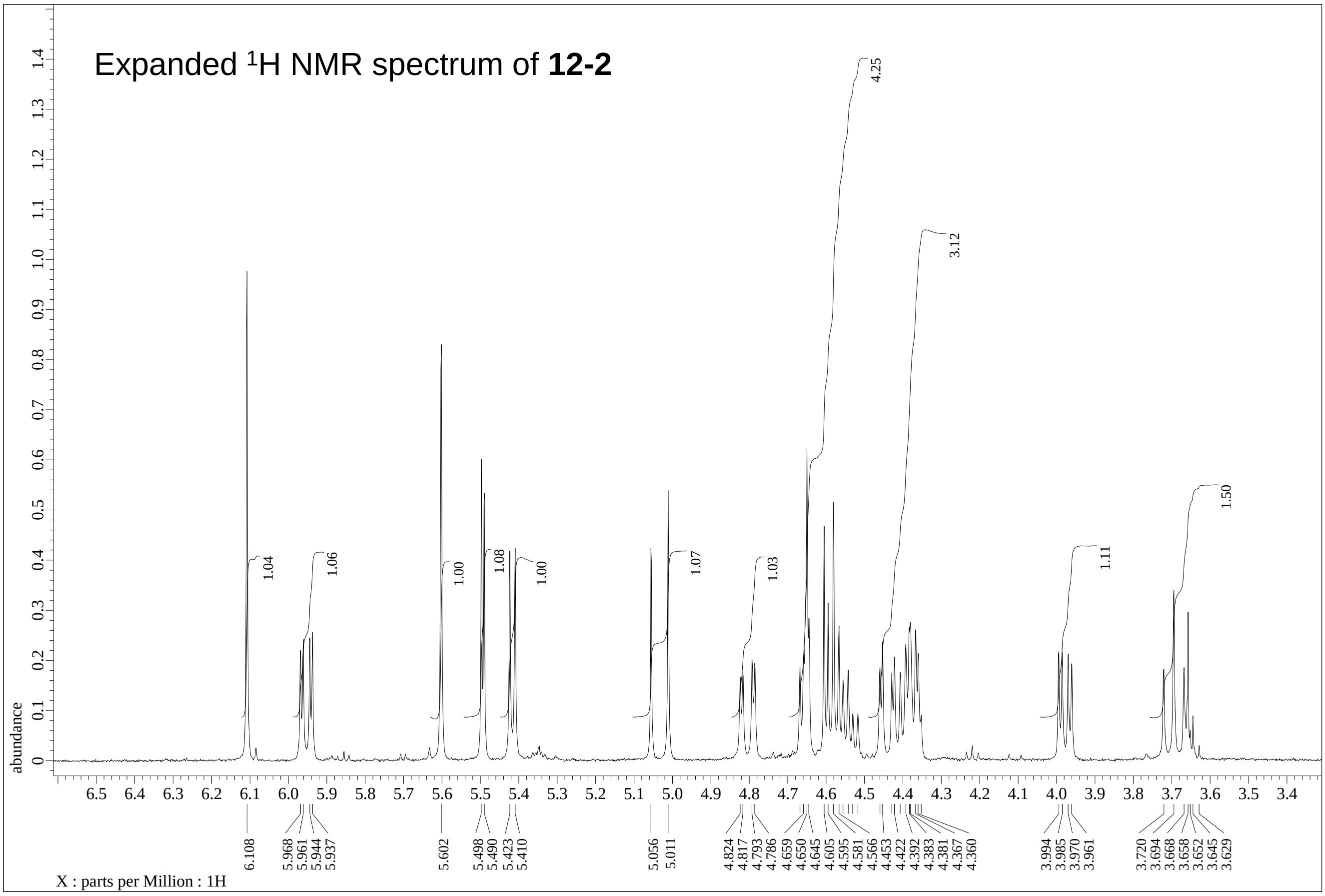

Expanded ¹H NMR spectrum of 12-2
abundance
X : parts per Million : 1H

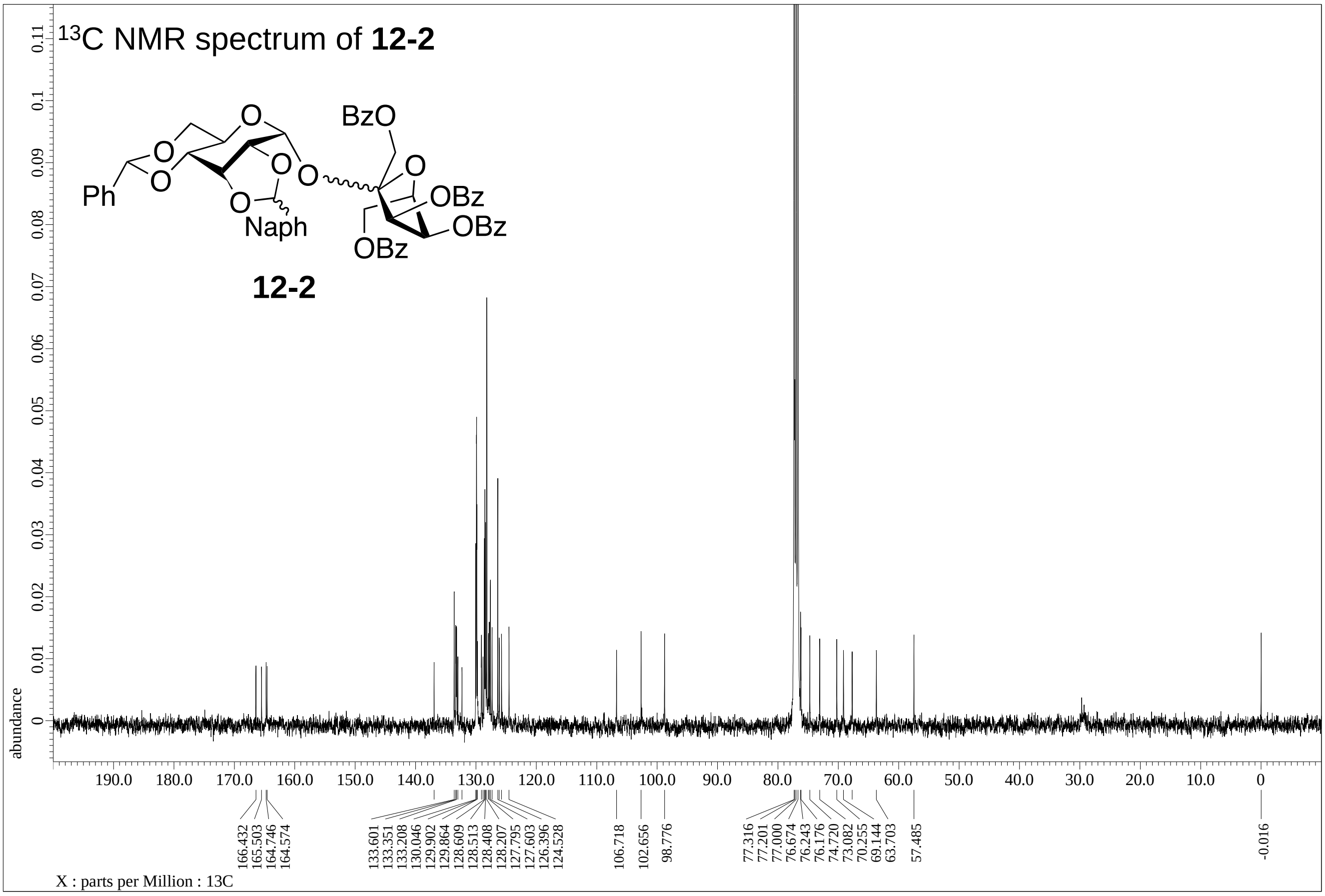

13C NMR spectrum of 12-2
BzO
O
O
O
O
O
O
Ph
Naph
OBz
OBz
OBz
12-2
abundance
190.0 180.0 170.0 160.0 150.0 140.0 130.0 120.0 110.0 100.0 90.0 80.0 70.0 60.0 50.0 40.0 30.0 20.0 10.0 0
166.432 165.503 164.746 164.574
133.601 133.351 133.208 130.046 129.902 129.864 128.609 128.513 128.408 128.207 127.795 127.603 126.396 124.528
106.718 102.656 98.776
77.316 77.201 77.000 76.674 76.243 76.176 74.720 73.082 70.255 69.144 63.703
57.485
-0.016
X : parts per Million : 13C

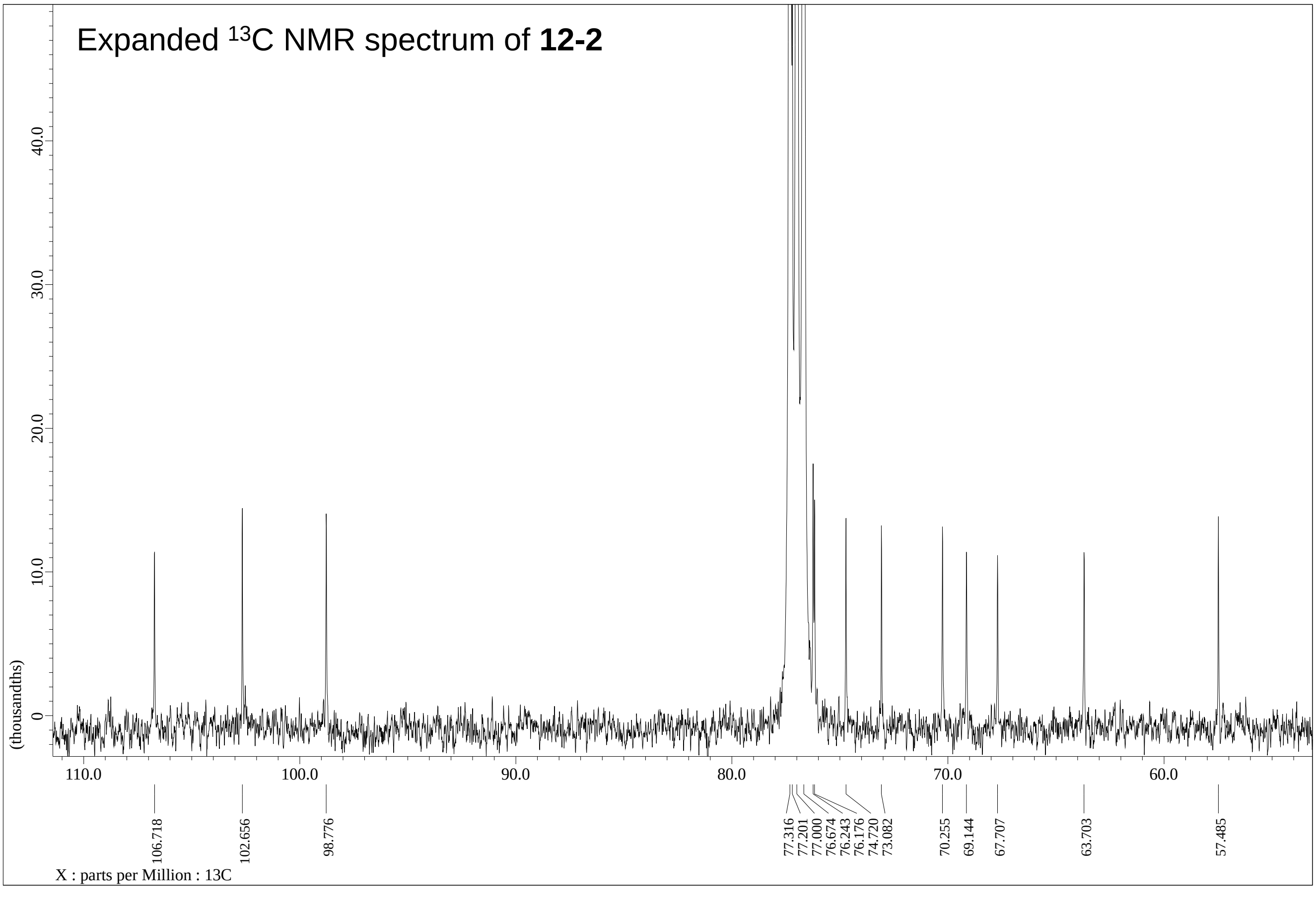

Expanded 13C NMR spectrum of 12-2
(thousandths)
110.0 100.0 90.0 80.0 70.0 60.0
106.718 102.656 98.776
77.316 77.201 77.000 76.674 76.243 76.176 74.720 73.082
70.255 69.144 67.707 63.703 57.485
X : parts per Million : 13C

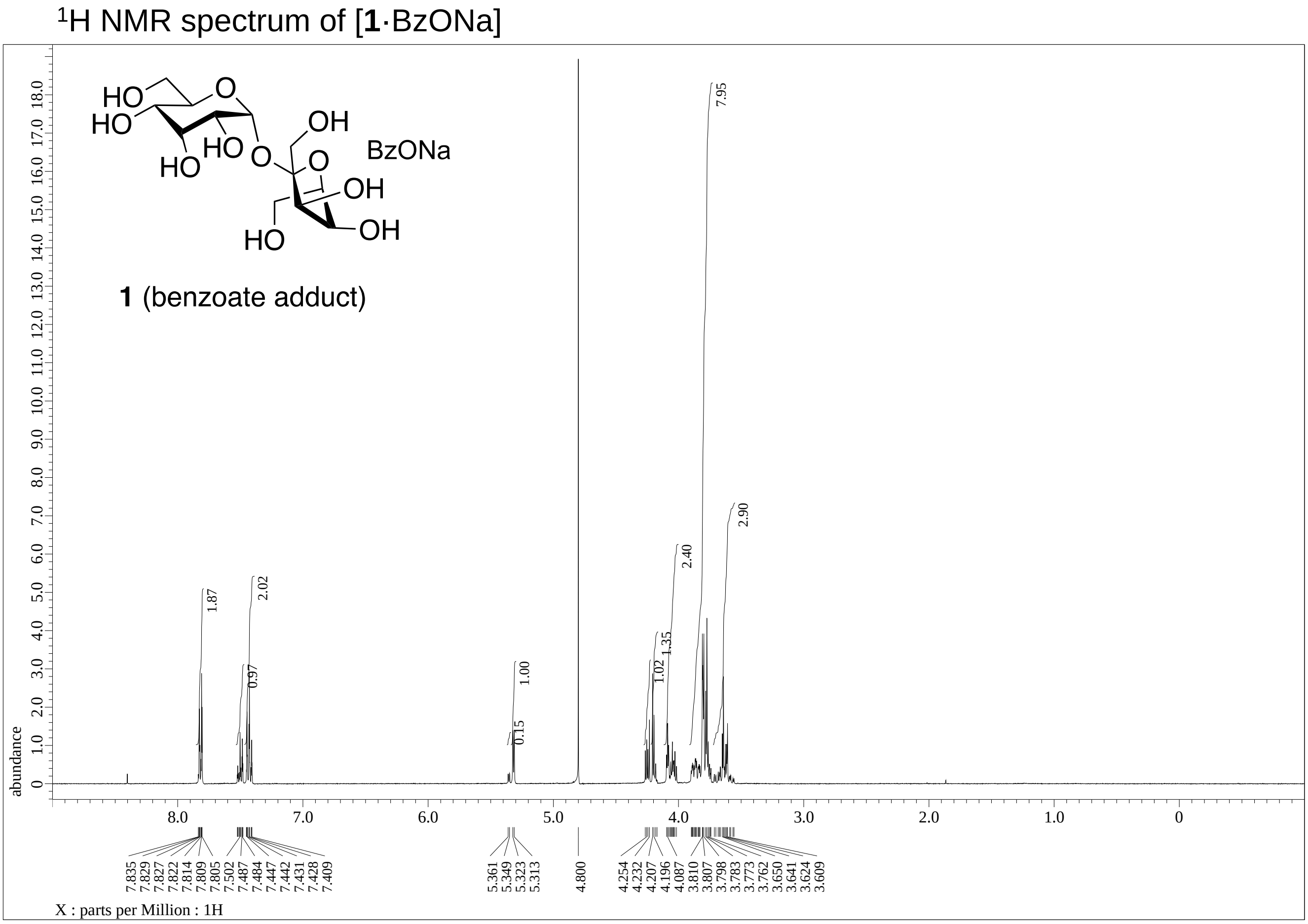
1H NMR spectrum of [1·BzONa]
BzONa
1 (benzoate adduct)
abundance
X : parts per Million : 1H

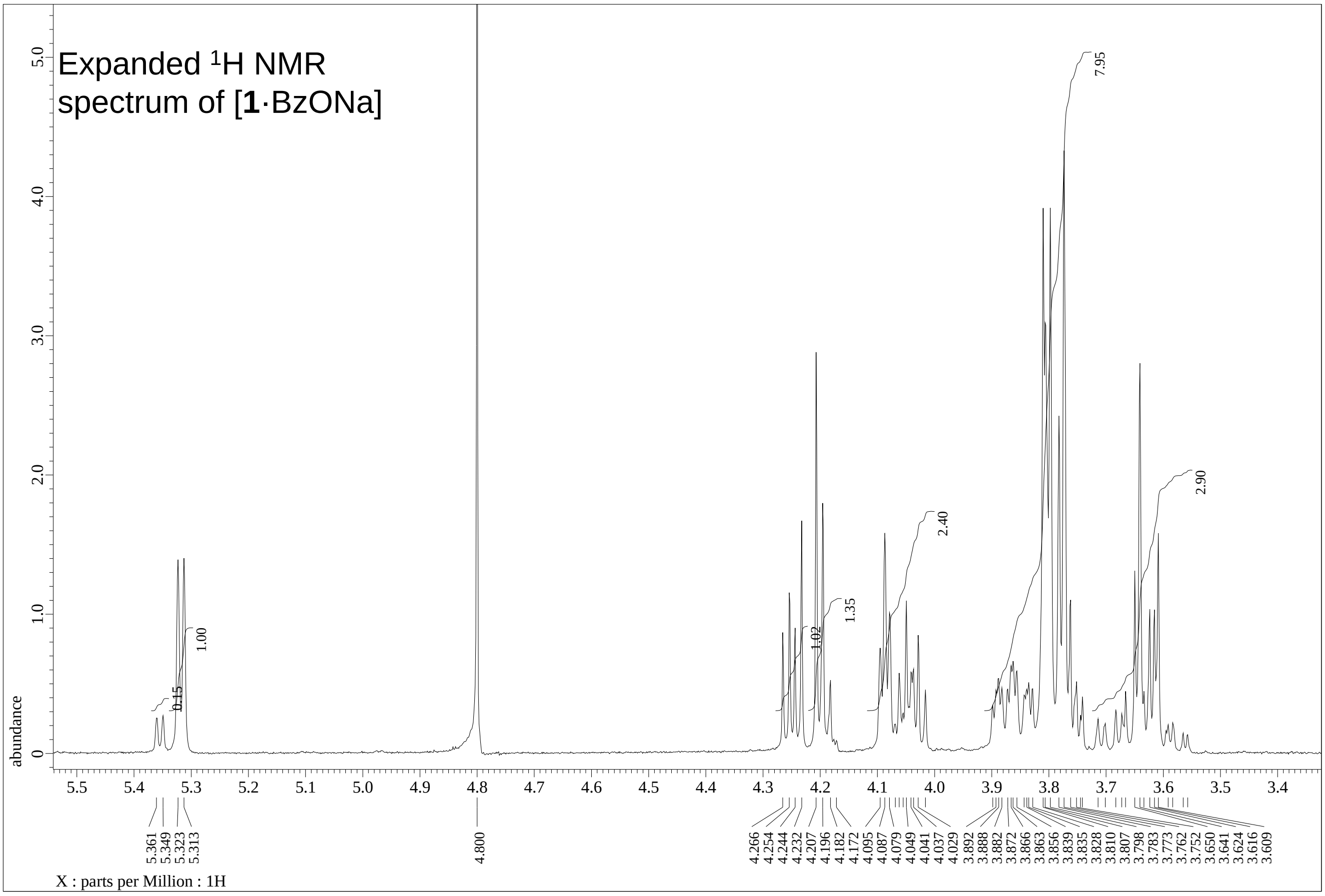
Expanded 1H NMR spectrum of [1·BzONa]
abundance
X : parts per Million : 1H

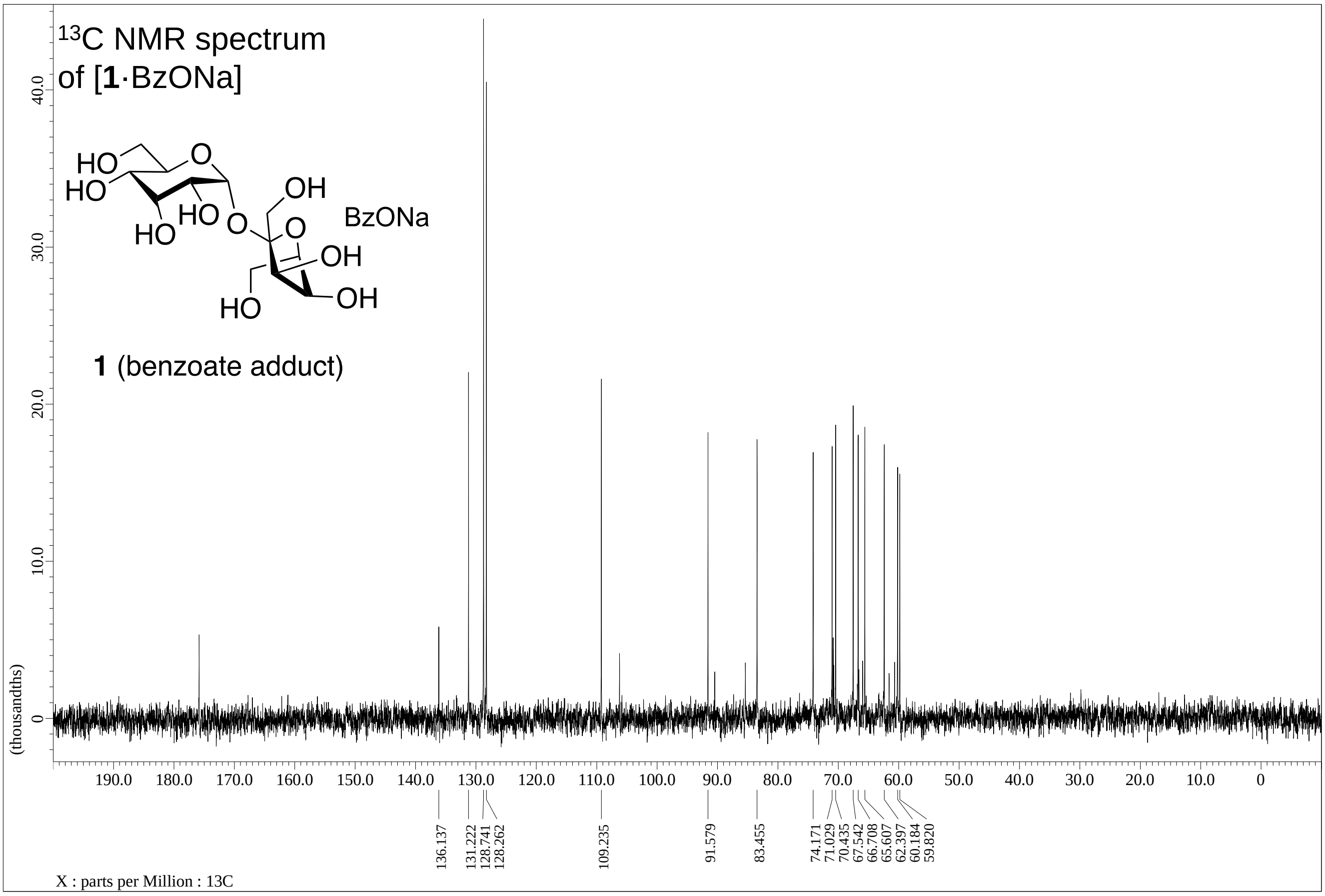
^{13}C NMR spectrum of [**1**·BzONa]
HO
HO
HO
HO
O
O
OH
O
OH
OH
HO
BzONa
1 (benzoate adduct)
(thousandths)
0
10.0
20.0
30.0
40.0
190.0 180.0 170.0 160.0 150.0 140.0 130.0 120.0 110.0 100.0 90.0 80.0 70.0 60.0 50.0 40.0 30.0 20.0 10.0 0
136.137
131.222
128.741
128.262
109.235
91.579
83.455
74.171
71.029
70.435
67.542
66.708
65.607
62.397
60.184
59.820
X : parts per Million : 13C

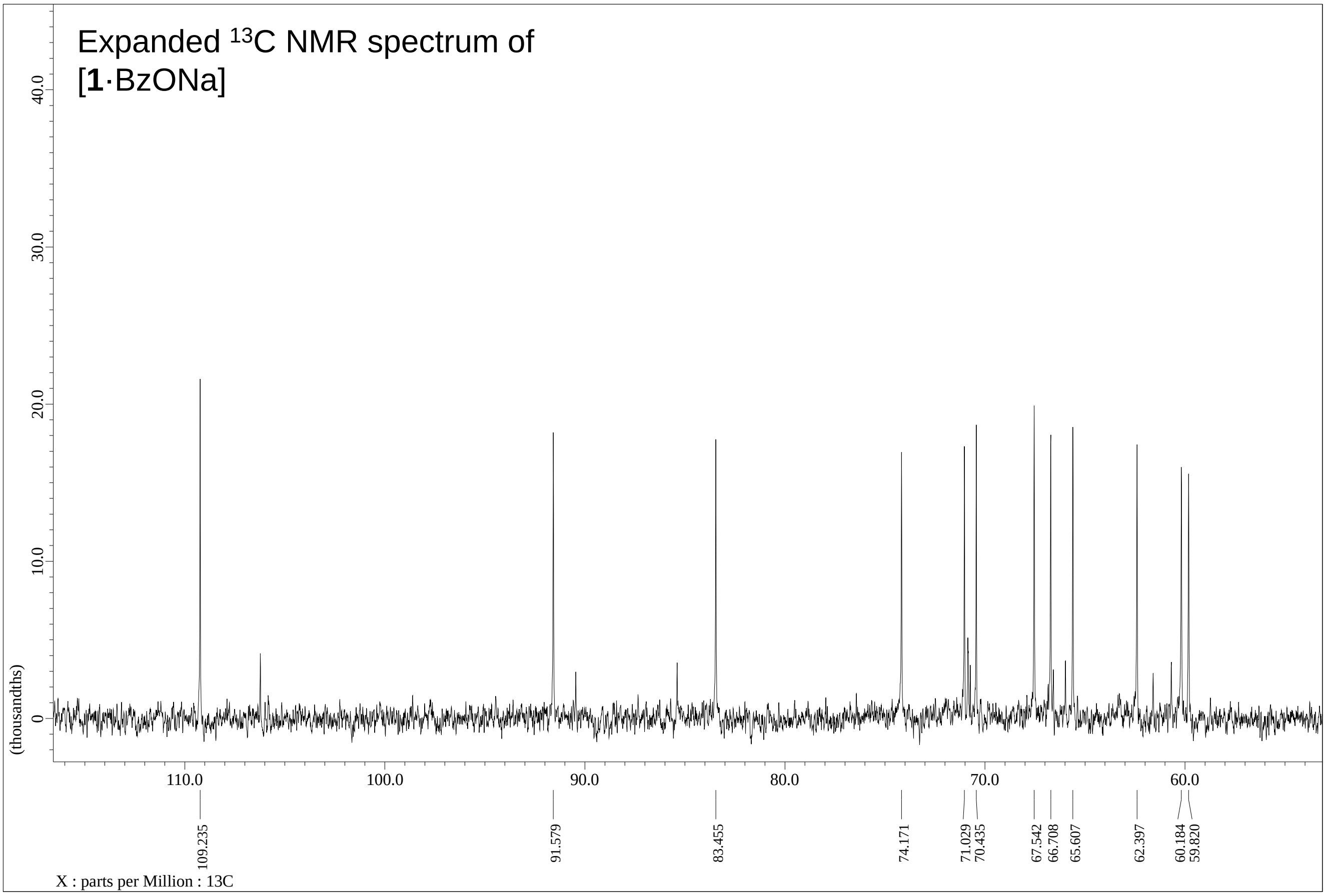
Expanded ^{13}C NMR spectrum of [**1**·BzONa]
(thousandths)
0
10.0
20.0
30.0
40.0
110.0 100.0 90.0 80.0 70.0 60.0
109.235
91.579
83.455
74.171
71.029
70.435
67.542
66.708
65.607
62.397
60.184
59.820
X : parts per Million : 13C

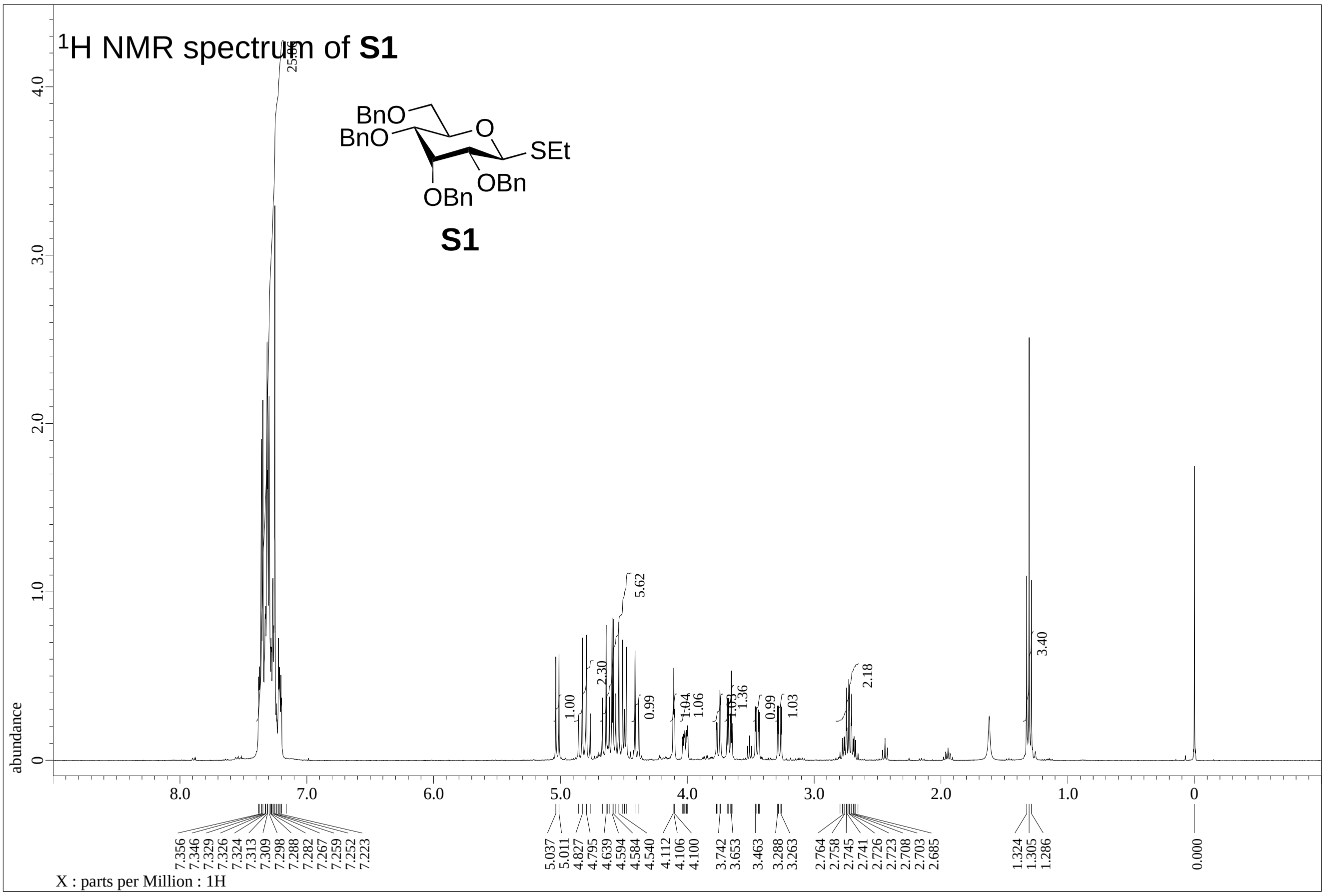

1H NMR spectrum of S1
BnO
BnO
O
SEt
OBn
OBn
S1
abundance
X : parts per Million : 1H

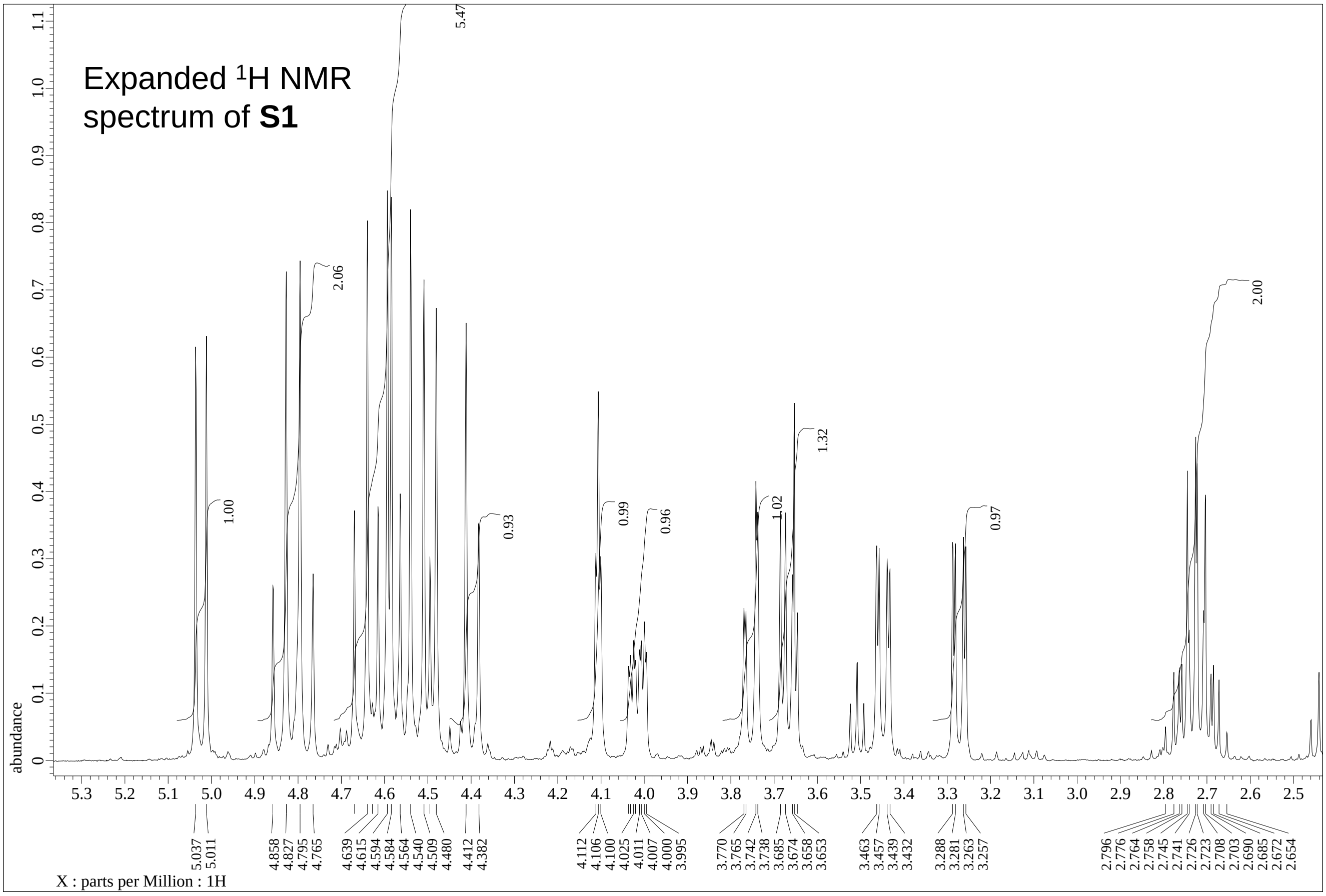

Expanded 1H NMR spectrum of S1
abundance
X : parts per Million : 1H

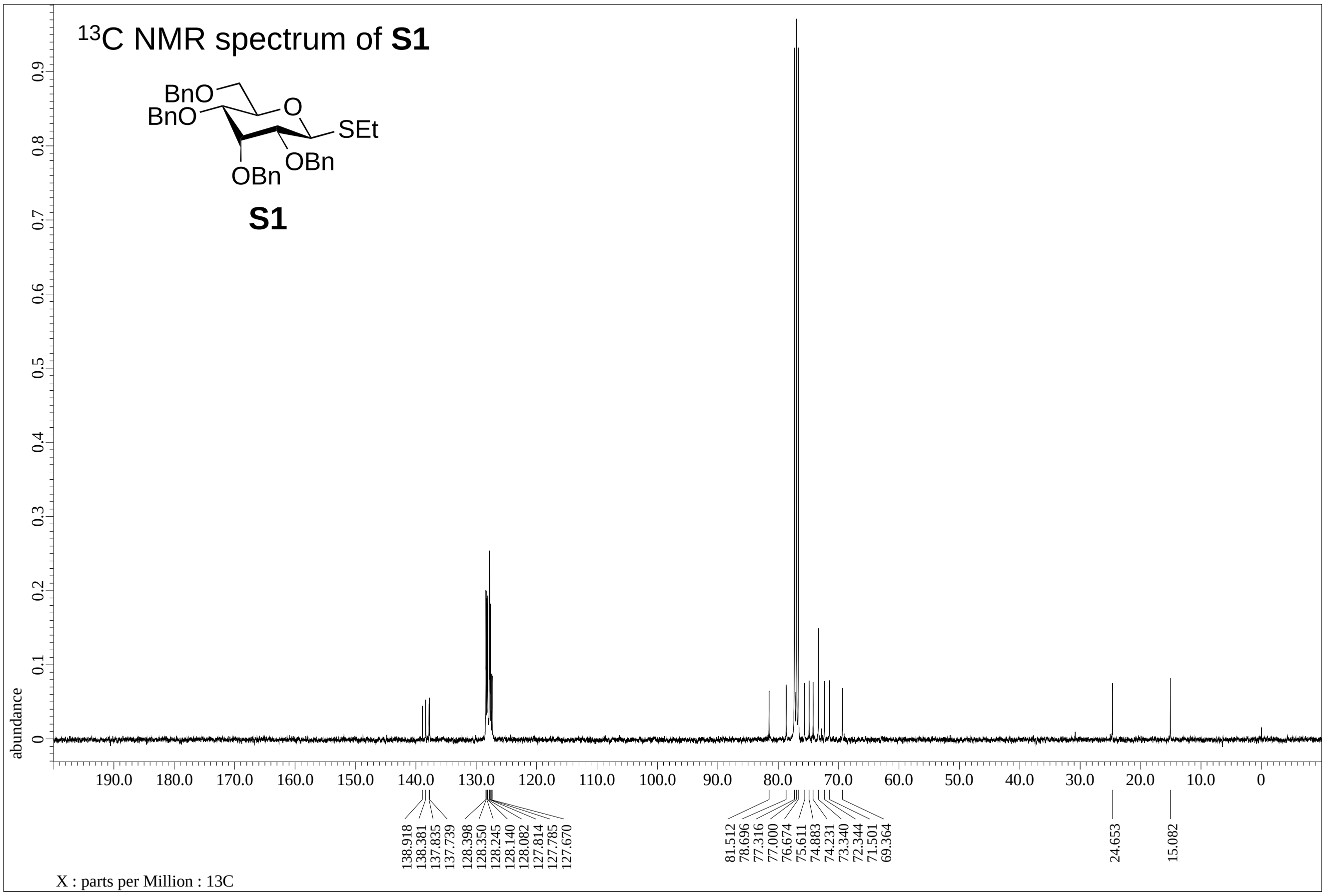
13C NMR spectrum of S1
BnO
BnO
O
SEt
OBn
OBn
S1
abundance
X : parts per Million : 13C
138.918
138.381
137.835
137.739
128.398
128.350
128.245
128.140
128.082
127.814
127.785
127.670
81.512
78.696
77.316
77.000
76.674
75.611
74.883
74.231
73.340
72.344
71.501
69.364
24.653
15.082

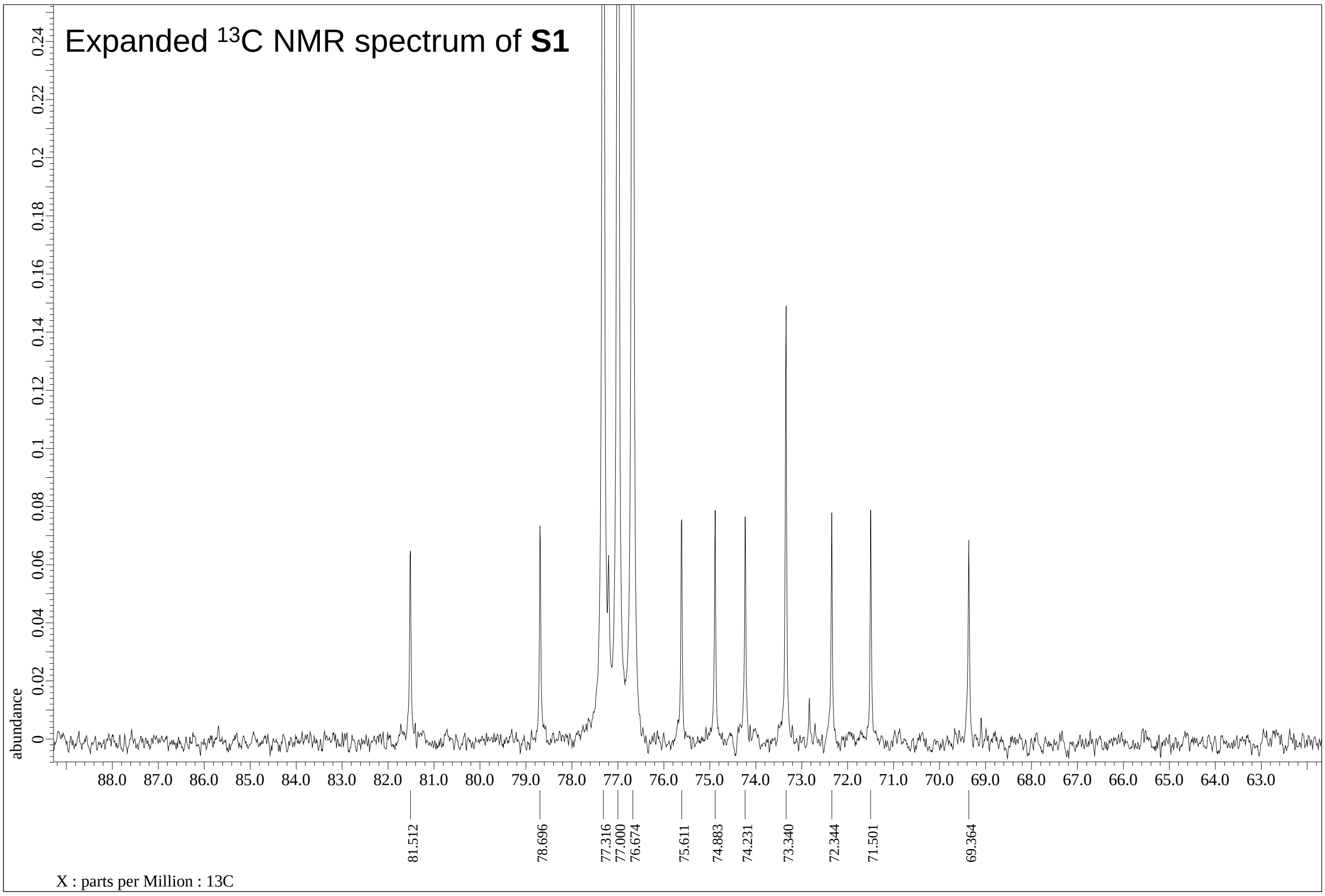
Expanded 13C NMR spectrum of S1
abundance
X : parts per Million : 13C
81.512
78.696
77.316
77.000
76.674
75.611
74.883
74.231
73.340
72.344
71.501
69.364

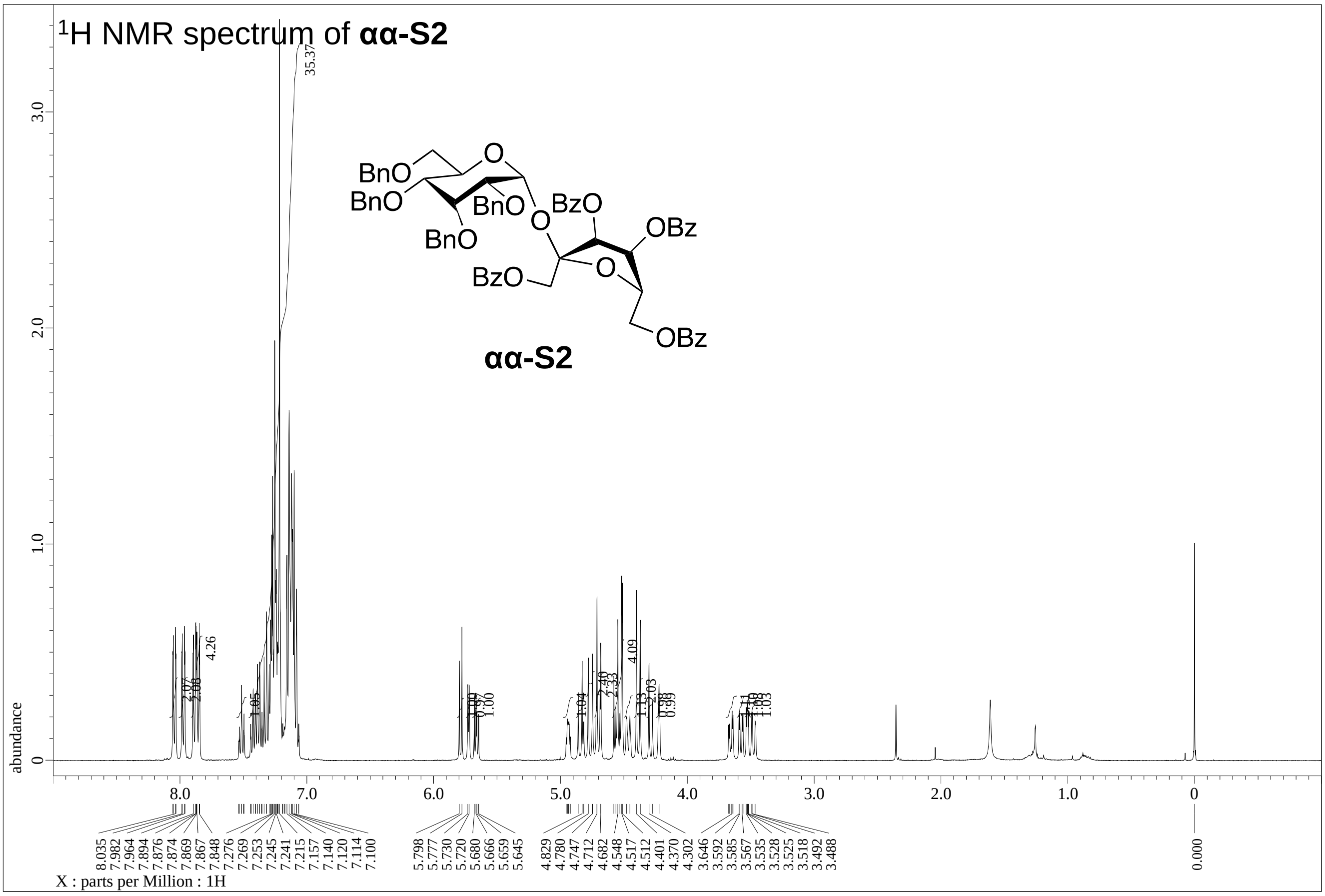

1H NMR spectrum of αα-S2
αα-S2
BnO
BnO
BnO
BnO
BzO
OBz
BzO
OBz
abundance
X : parts per Million : 1H

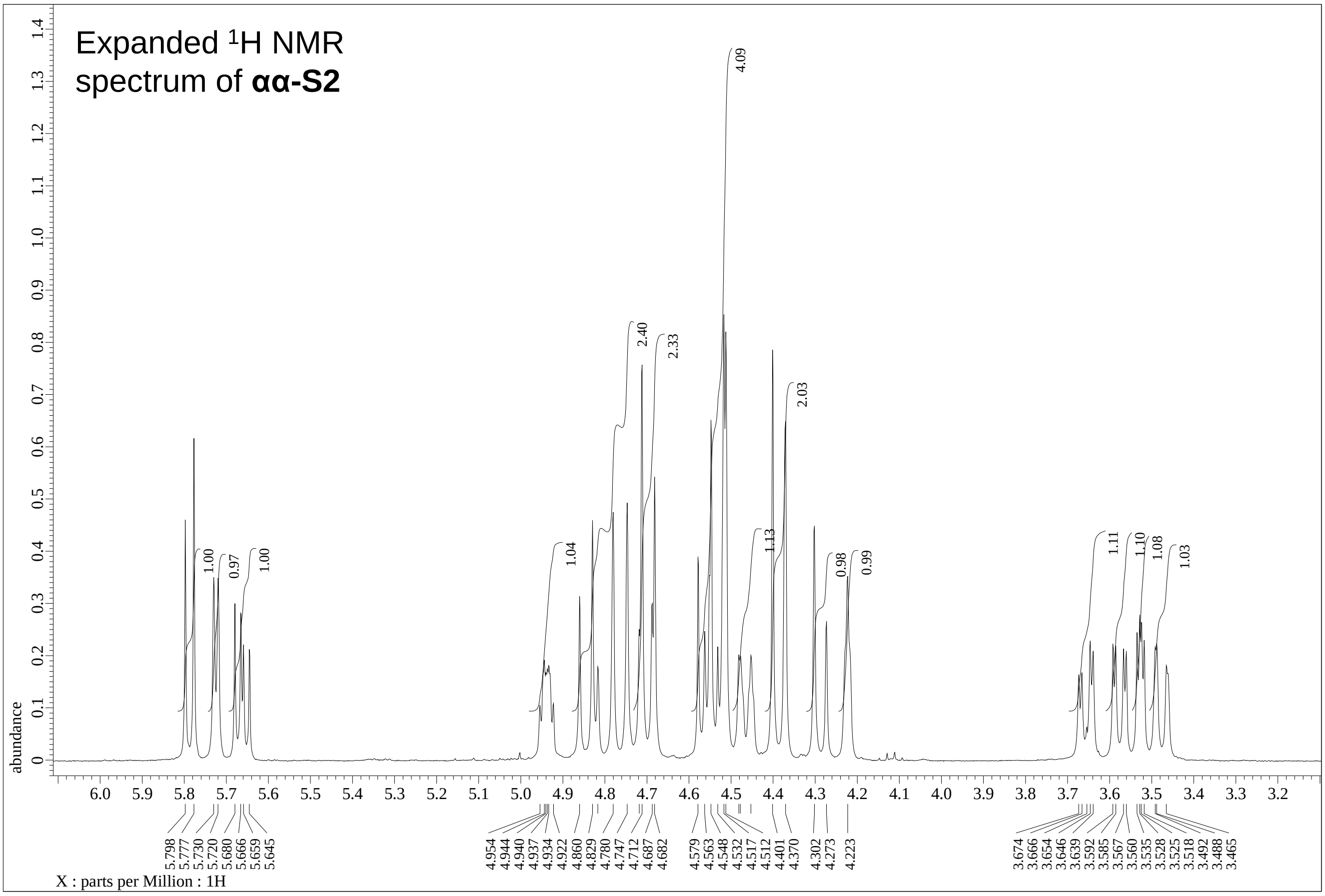

Expanded 1H NMR spectrum of αα-S2
abundance
X : parts per Million : 1H

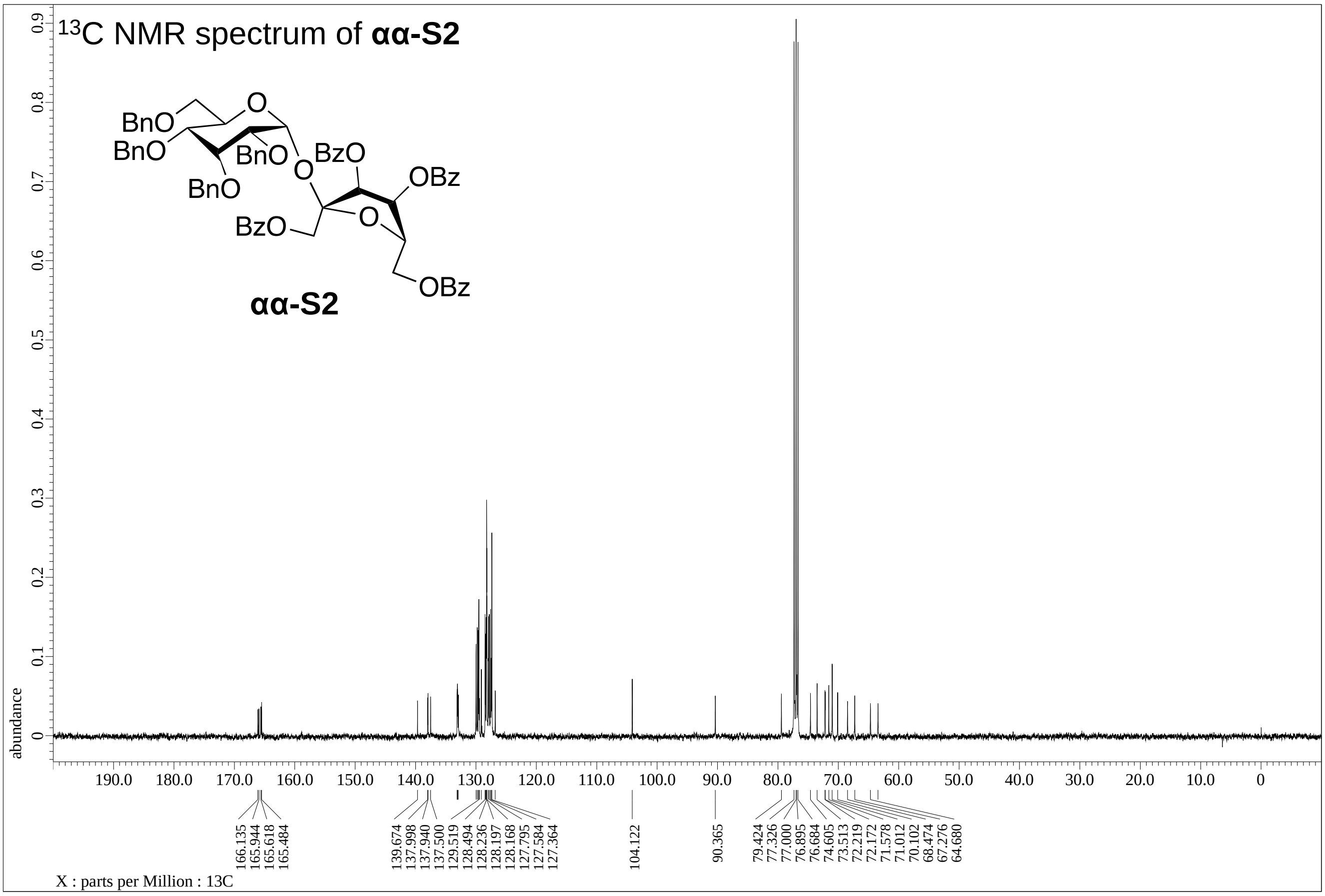

13C NMR spectrum of αα-S2
BnO
BnO
BnO
BnO
BzO
OBz
BzO
OBz
αα-S2
abundance
X : parts per Million : 13C
166.135
165.944
165.618
165.484
139.674
137.998
137.940
137.500
129.519
128.494
128.236
128.197
128.168
127.795
127.584
127.364
104.122
90.365
79.424
77.326
77.000
76.895
76.684
74.605
73.513
72.219
72.172
71.578
71.012
70.102
68.474
67.276
64.680

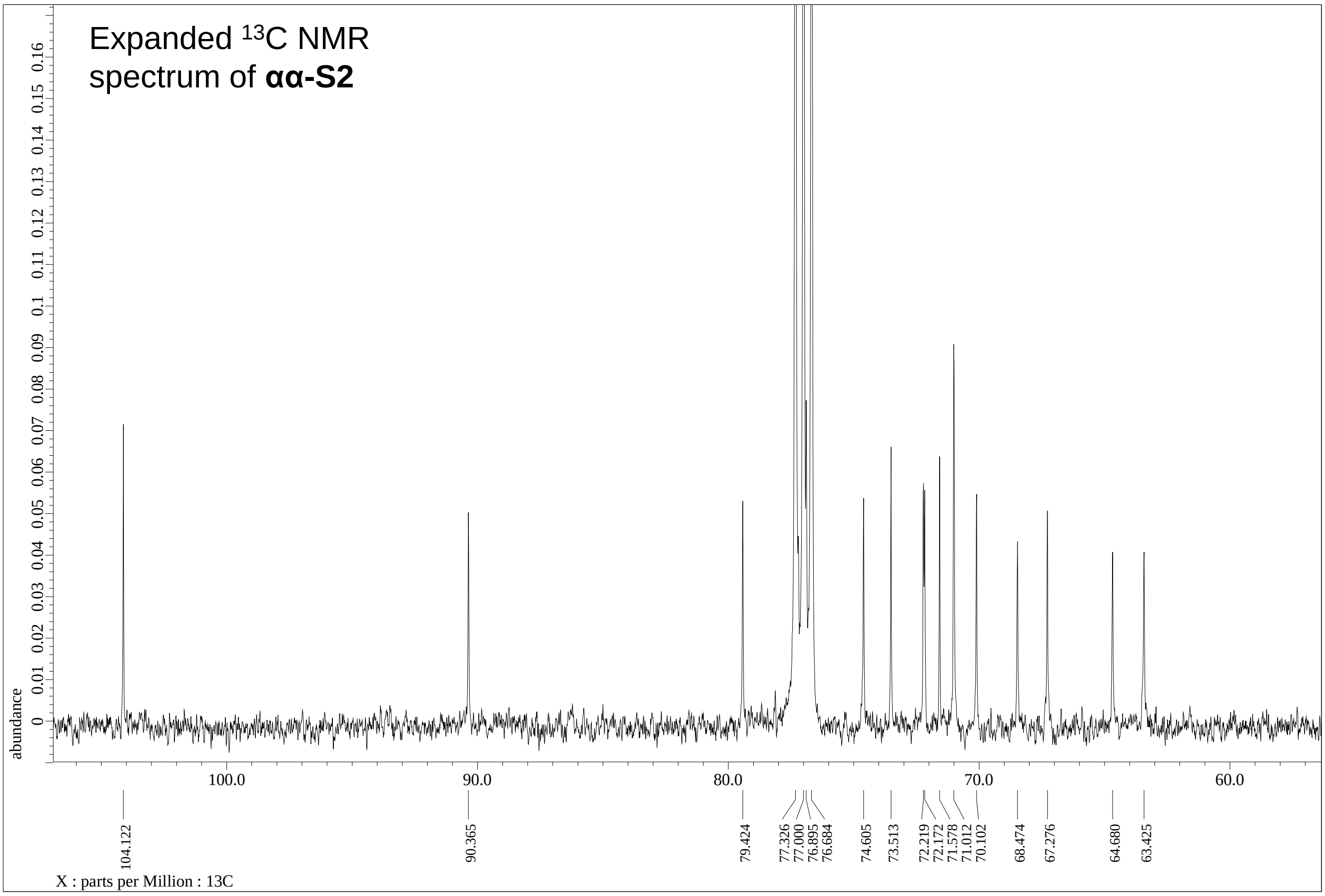

Expanded 13C NMR spectrum of αα-S2
abundance
X : parts per Million : 13C
104.122
90.365
79.424
77.326
77.000
76.895
76.684
74.605
73.513
72.219
72.172
71.578
71.012
70.102
68.474
67.276
64.680
63.425

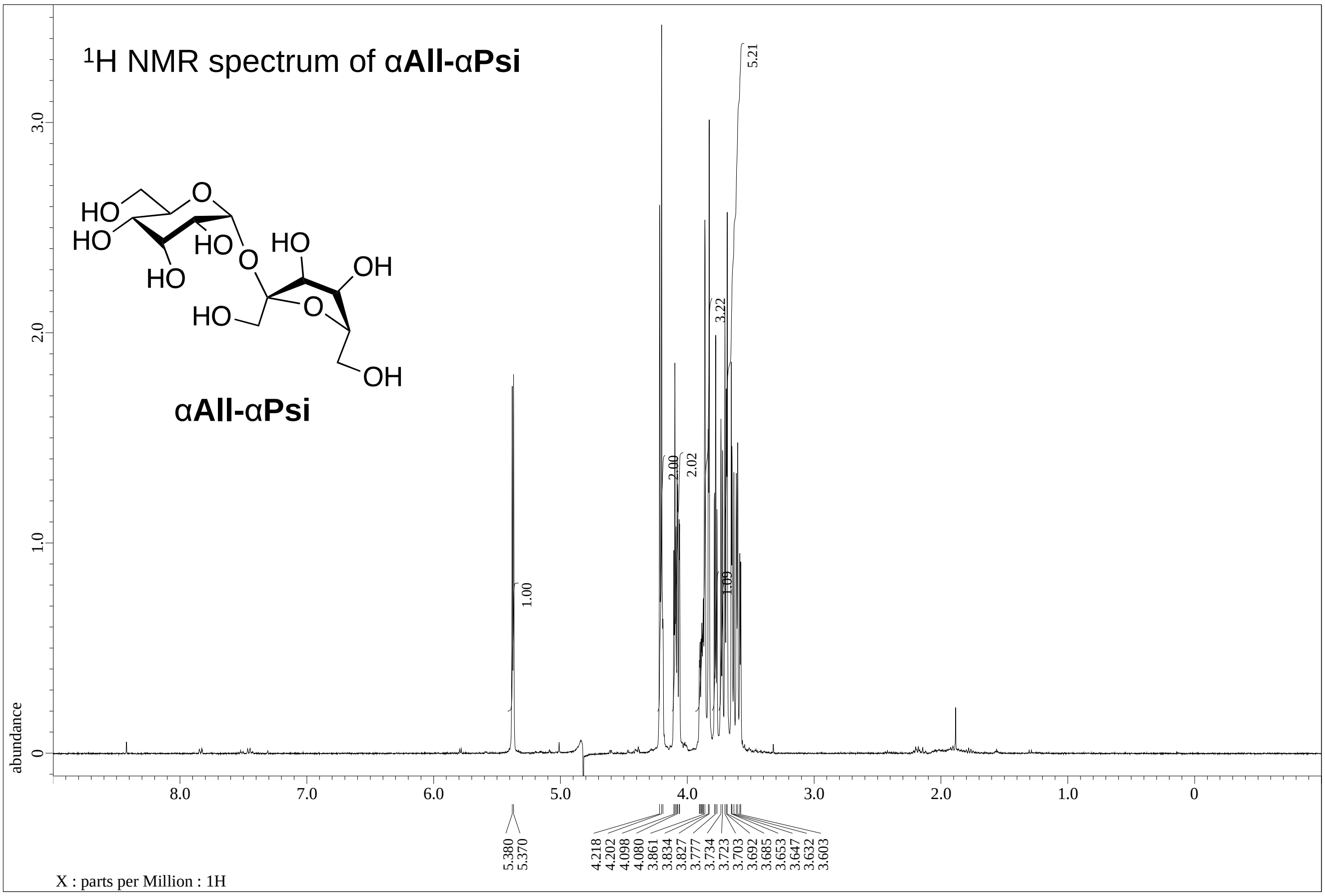

^{1}H NMR spectrum of α**All**-α**Psi**
α**All**-α**Psi**
abundance
X : parts per Million : 1H

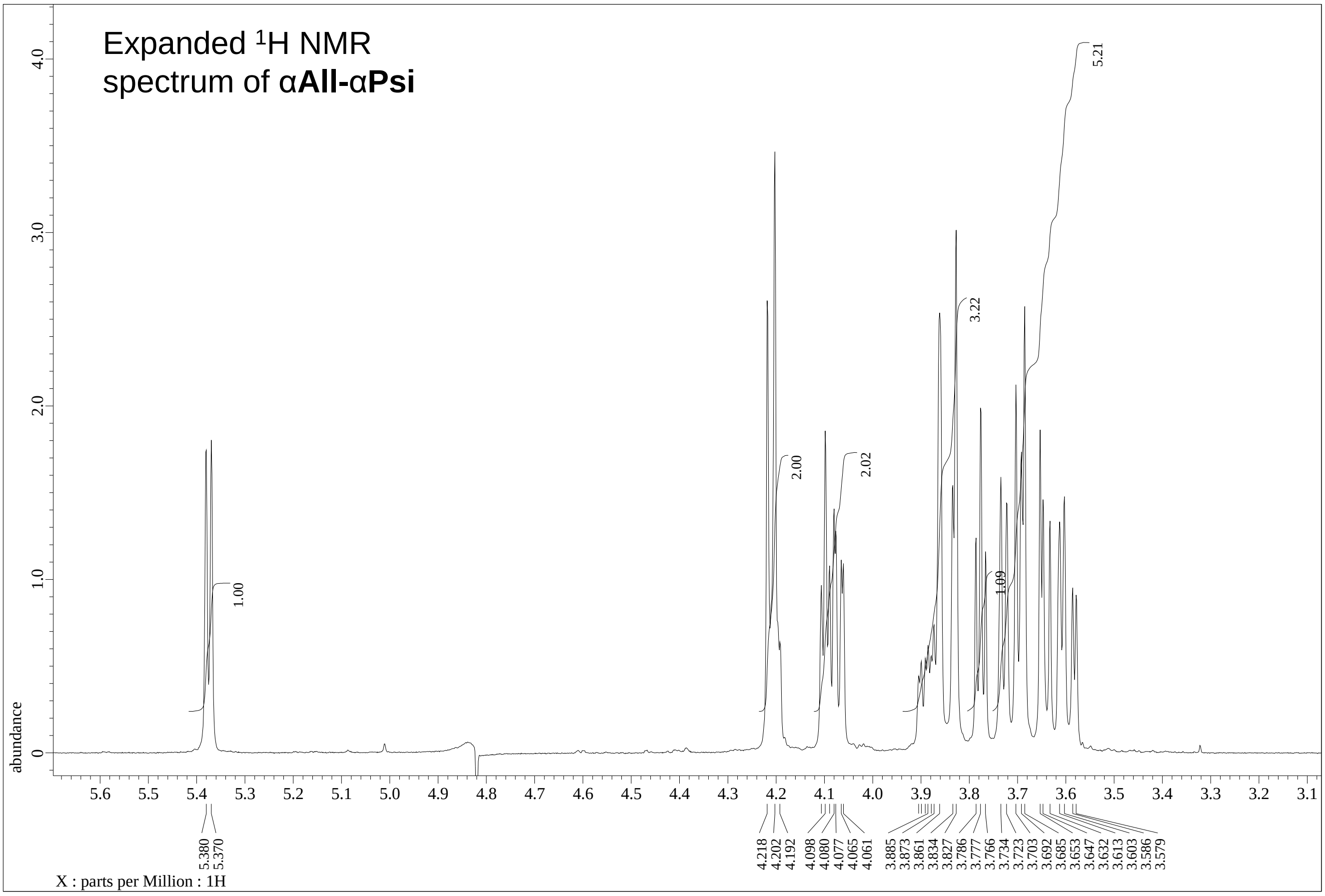

Expanded ^{1}H NMR spectrum of α**All**-α**Psi**
abundance
X : parts per Million : 1H

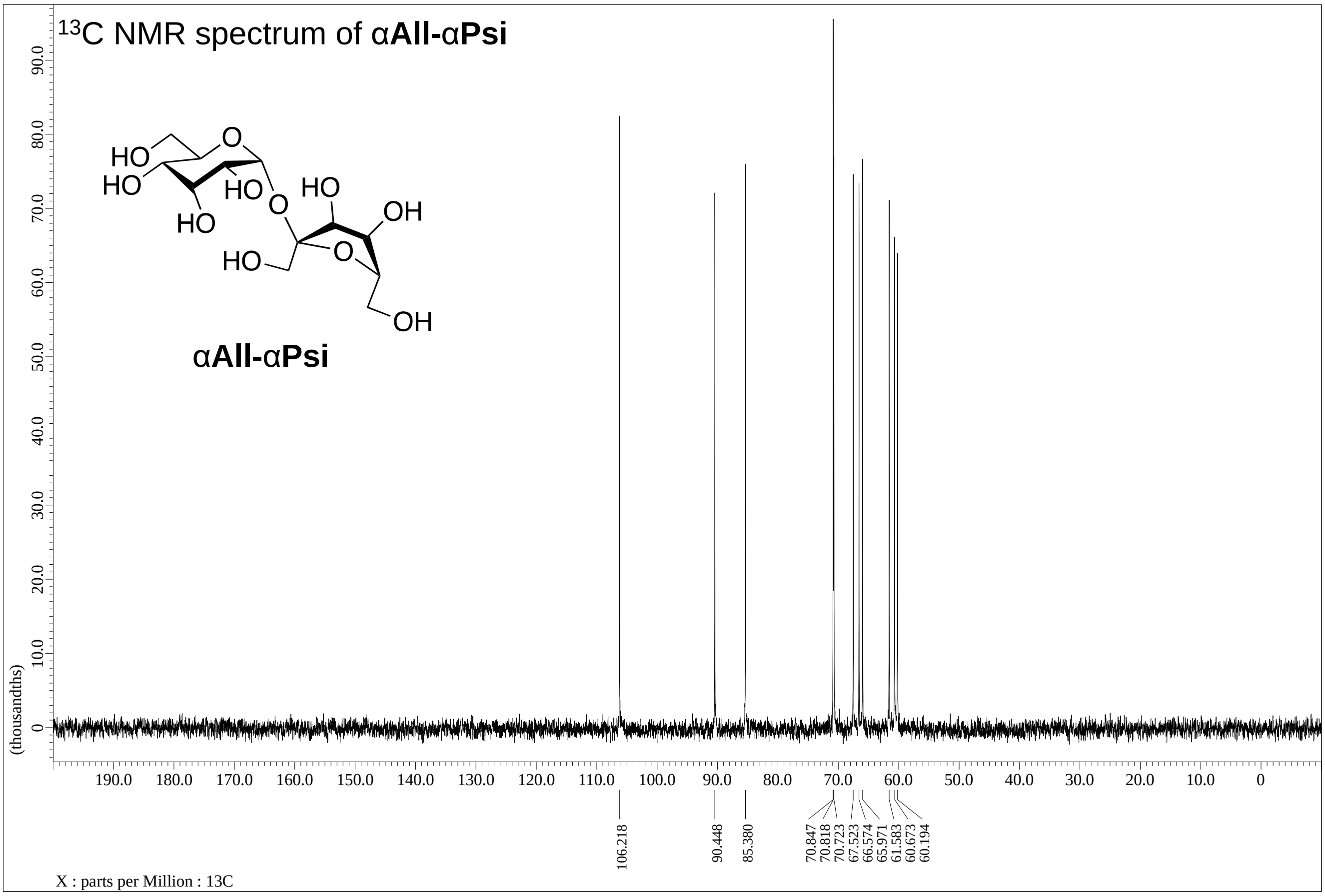
13C NMR spectrum of αAll-αPsi
αAll-αPsi
(thousandths)
X : parts per Million : 13C
106.218
90.448
85.380
70.847
70.818
70.723
67.523
66.574
65.971
61.583
60.673
60.194

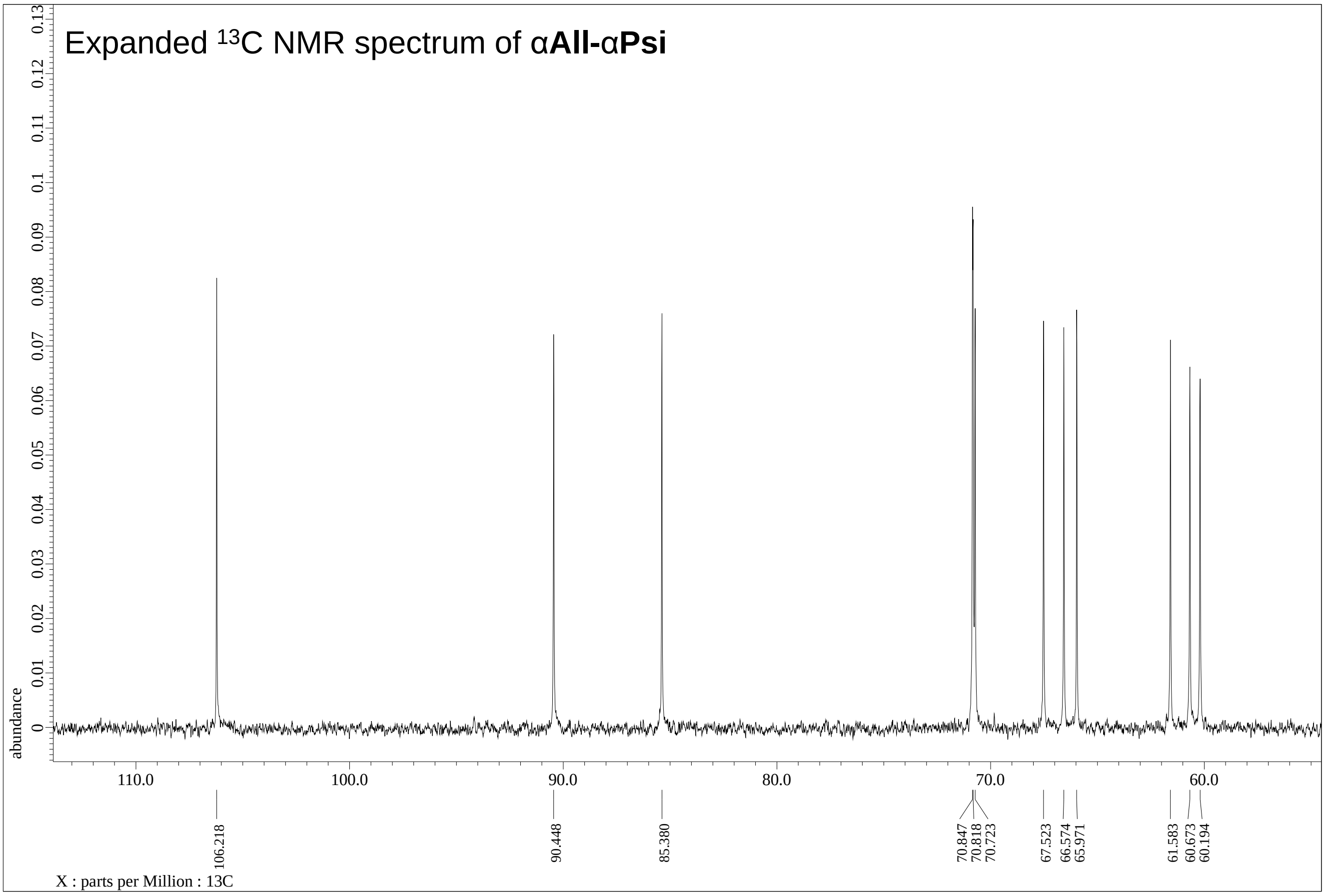
Expanded 13C NMR spectrum of αAll-αPsi
abundance
X : parts per Million : 13C
106.218
90.448
85.380
70.847
70.818
70.723
67.523
66.574
65.971
61.583
60.673
60.194
